# International guidelines to inform policy development to address client violence in South Africa: an ATA-document analysis

**DOI:** 10.1186/s12913-022-08196-8

**Published:** 2022-08-12

**Authors:** Lucé Pretorius, Alida G. Herbst

**Affiliations:** 1 Social Worker in Private Practice and Doctoral Student in the Community Psychosocial Research Entity (COMPRES), North-West University, Potchefstroom, South Africa; 2 Professor of Social Work and Researcher in the Community Psychosocial Research Entity (COMPRES), North-West University, Potchefstroom, South Africa

**Keywords:** Social workers, Client violence, Safety and protection, Workplace violence, Safeguarding initiatives, Policies

## Abstract

**Background:**

Research shows that the most typical response to client violence has been to implement policies that safeguard social workers at their workplaces. This article examined, through a document analysis, the international norms for the protection of social workers. The goal of the document analysis was to inform policy development in South Africa against client violence.

**Methods:**

The researchers found, selected, analysed, and synthesised 17 international policies, frameworks, protocols, guidelines, and legislative frameworks using the applied thematic analysis (ATA) approach. The data was analysed at three levels, and open coding yielded 18 codes.

**Results:**

The codes were refined into three main themes and subthemes related to protecting social workers from client violence: (1) employers inspired a culture of safety and security within the work-environment, (2) social workers prioritised their safety by using their clinical skills, and (3) actively implementing initiatives to ensure the safety of social workers.

**Conclusions:**

The research highlighted social work safety while providing services at an office, visiting sites, or traveling. Examining these practicalities provided valuable data that can inform policy development processes in different countries.

**Supplementary Information:**

The online version contains supplementary material available at 10.1186/s12913-022-08196-8.

## Background

The safety of social workers is a complicated but important issue [1]. According to the Department of Health and Human Services in the United States [2], this issue not only involves the prevention of the fear or actuality of physical violence in the workplace, but also encouraging psychological and emotional safety and resilience. Therefore, agencies / organisations / departments (hereafter referred to as AODs) must address all aspects of social worker safety in their policies and procedures so that they can foster a culture and atmosphere that promotes worker safety [3].

Pollack [4] expressed concern to the international social work community more than 10 years ago, stating that the time has come for the profession to adopt standards, conventions, and agreements to recommend ways to engage with violent clients. Sioco [5] examined the standards, conventions, and agreements of AODs to protect social workers against client violence and found evidence of three programmes in the United States of America. First, the Massachusetts Department of Children and Families (DCF) provided social workers with safety handbooks outlining their procedures, as well as training on how to manage violent situations with clients. Every social worker in this department was issued with a cell phone that would allow them to communicate with their superiors and law enforcement. In addition, in the event of a suspected violent client, this department implemented a system where at least two social workers were required when conducting a home visit. DCF workers were also required to notify law enforcement and seek a police escort to accompany them in the event of risks [5]. The second programme, *OK Connect*, by the Child Welfare League of America (CWLA) in Miami, Florida, focused on the protection and safety of social workers through technology. Each social worker was given a Samsung Blackjack cell phone or a Panasonic laptop that was linked to a GPS internet system that alerted management to their location. By clicking a button on their phone or laptop, social workers could also set off an alarm, alerting their supervisors and management when their safety was in jeopardy [5]. During the same period, the Philadelphia Department of Human Services trialled a programme in which 25 caseworkers were provided mobile devices to contact response services more easily in the event of an emergency [5].

The most typical response to client violence has been to adopt policies to protect social workers [6]. Since 2010, California, New Jersey, Washington, Vermont, and Kentucky have been among the states that have enacted safety requirements for social workers [2, 7]. Several states of the United States have also addressed worker safety through legislation and regulations aimed at preventing workplace violence and making workplaces safer for social workers. On three occasions, bills to improve social worker safety have been submitted to the United States Congress, but none have been passed.

In other regions of the world, there is less research on formal programmes, policies, and legislation. Even individual contributions and personal records are limited compared to those from the United States. On reviewing literature from South Africa, it became apparent that there are no policies, frameworks, protocols or guidelines to safeguard social workers from client violence. During qualitative interviews with 16 South African social workers (as part of a multi-phased research project titled *A policy framework to enhance the protection of South African social workers against client violence)* in 2021, no participant was able to identify any formal policy, framework, protocol or guideline to enhance the safety of social workers that has been implemented by a South African social work employer [8]. It became evident that formal programmes and policies about the protection of social workers in the line of duty are limited or non-existing.

By analysing existing international documentation, one can gain knowledge of policy content across time and space and learn how information and ideas about phenomena are presented in formal documents. In this article, the researcher examined international policies, frameworks, protocols and guidelines, by means of a document analysis, to create convergence and corroboration and inform the development of a policy framework to protect South African social workers against client violence.

## Methods

Document analysis involves an iterative process of superficial examination (skimming), thorough examination (reading), and interpretation [9]. As a result, it is a method for finding, selecting, analysing, and synthesising data from printed and electronic sources. The document analysis in this study was conducted using applied thematic analysis (ATA) as the methodology. ATA is specifically tailored to help researchers be organised and systematic in their planning and preparation for text-based qualitative analysis [10].

**Fig. 1:**
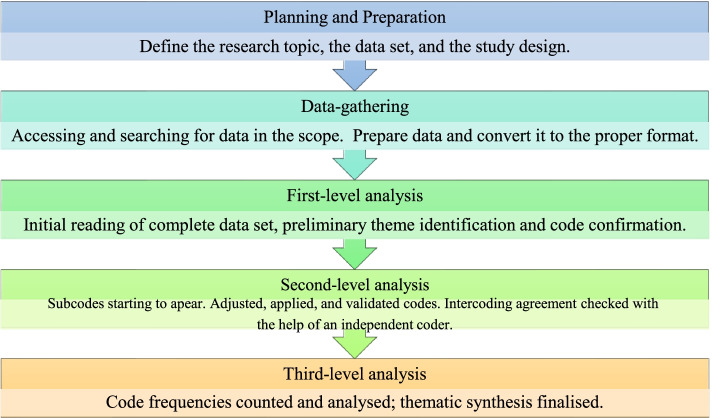
Application of ATA to document analysis [12]

According to Guest et al. [11], ATA also incorporates an inductive analysis framework and synthesises epistemological and methodological perspectives, including positivism, interpretivism, phenomenology, applied research, and grounded theory. As a result, the coding and subsequent analyses accommodated multiple analytical approaches and supported the researcher's exploratory approach. Furthermore, the focus on text contributed to the transparency and reproducibility of the study due to the use of valid, high-quality, and accessible data sources [11]. Figure 1 illustrates the application of the ATA to document analysis, with each phase outlined in the discussion that follows.

### Planning and preparation

Planning and preparation were the first steps of the ATA used for this document analysis. This involved defining the exact research question the document analysis would answer, as well as a preliminary scan of the available data. Tentative key words (social work*, client violence*, policy*) assisted the researcher to comb through resources of the National Association of Social Work (NASW) in the United States of America and define the following research question: *What international policies, frameworks, protocol and guidelines can inform the development of a policy framework to enhance the protection of South African social workers from client violence?*

To generate an audit trail of the ATA process, each choice and the rationale behind it was recorded in a single planning document. The researcher also started the development of a codebook to define and describe how data will be analysed and how the rules for counting the frequencies of occurrence of the codes would be applied [13].

### Data gathering

**Fig. 2:**
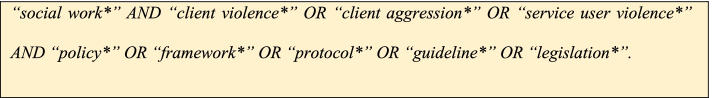
Boolean string for OneSearch

The data considered to be relevant to the study were gathered during the second phase of the document analysis. Due to the limited amount of data available, the researcher used NASW resources and the international policies, frameworks, procedures, guidelines, and legislation identified by the North-West University's (NWU) OneSearch engine, which reaches 252 databases. After consulting with a NWU librarian, the following Boolean search string, as illustrated in Fig. 2, was identified:

The final data set, consisting of 54 records, was uploaded to ATLAS.ti Qualitative Data Analysis Research Software. All documents were checked and rechecked to ensure accurate conversion to ATLAS.ti. Any documents that were more than ten years old were discarded. Since there were few resources for translating literature in other languages, searches were confined to articles in English. The following criteria were used to select studies:

- Documents outlining international policies, frameworks, procedures, and guidelines on client violence and/or client aggression directed at social workers, student social workers, counsellors, and therapists.
- Proposed policy and pending legislation to protect social workers, student social workers, counsellors, and therapists against client violence and/or hostility.

Studies were excluded based on the following criteria:

- International policies, frameworks, procedures, and guidelines concerning workplace hazards or components outside of the working environment and/or service areas.
- International policies, frameworks, procedures, and guidelines to protect social workers, student social workers, counsellors, and therapists from intra-organizational violence or violence among co-workers.

This resulted in a data set of 17 records totalling 454 pages for the first level analysis.

### First-level analysis

This subsequent phase centred on a comprehensive review and preliminary analysis of the complete data set's contents. A key-word-in-context search was used as the locus for concepts in the text, as well as a word-by-word analysis to uncover preliminary themes. These themes were converted to matching codes and entered into the codebook. They were labelled, briefly defined, supplied with instructions on when to use the code and provided with exclusion criteria or comments. With an increased understanding of the concepts and identification of related codes, the codebook's determinations were regularly reviewed. A continual analysis of the alignment between the codes and the research question was conducted throughout the review process, and codes were subsequently either affirmed or rejected based on their congruence.

### Second-level analysis

The second level of analysis required multiple repetitions of the process of code development, coding, systematic analysis, checking for discrepancies and code refining before important sub-themes could be codified. The number of texts to which codes were applied varied. A coded text segment could be a whole sentence, a part of a sentence, or an entire paragraph. Following the development of sub-themes, the codes were applied to an uncoded copy of the entire data set, whereafter the data were sent to an independent coder. The purpose of the independent coder was to improve coding reliability and offer checks on individual biases and variability in code definition and interpretation. The inter-coder agreement tool of ATLAS.ti's was used to calculate an inter-coder agreement coefficient [14, 15]. As this tool can measure the statistical inference of reliability (the extent of agreement or disagreement between the reliability of different coders), it was used for the second-level analysis. An overall intercoder agreement value of α = 0.775 was determined, indicating that 77.5% of the data were coded to a higher degree than chance. It follows that the coders agreed and the data were statistically connected and significant [16, 17]. Additionally, coding checks enhanced the precision of the codebook through iterative modification.

### Third-level analysis

In accordance with the rationale of the study and the research question, the third stage of analysis involved applying pre-determined counting criteria to the codes in the data set. Analysing the frequency and co-occurrence of the coded words or phrases in international policies, frameworks, protocols, and guidelines, allowed to quantify the data. This contributed to the discovery of themes in the text and further analysis. Each theme's significance was demonstrated by comparing the code frequencies within the thematic content and the relationships between the codes within the dataset. The results were presented in a series of charts and tables depicting the various themes and sub-themes (identified and coded) that can be used to inform South African policy.

The textual data were sourced for a comprehensive range of quotes suggestive of each theme and subtheme and were exported to a Microsoft Excel file to enhance prompt extraction for the document analysis report. The code frequencies and thematic content were analysed and synthesised to be presented as findings.

## Results and Discussion

Open coding yielded 18 codes organised into three themes and subthemes. Due to the interconnected nature of many of the codes, researchers had to be cautious when synthesising and analysing the data. Table 1 displays the codes that were identified, while Table 2 shows the themes and subthemes that emerged. Table 3 shows the frequency of the codes within the specified themes.

**Table 1:** Codes and code definitions

Code no	Code name	Code definitions
**1**	**Prevention and management of client violence**	**Client violence is clinically managed to prevent or lessen the perceived level of threat. This intervention is frequently at an administrative or organisational level**
1.1	Data management	Collecting, storing, and using current and historical data about client violence in a secure, efficient, and cost-effective manner
1.2	Policies	The strategy or philosophy of an organisation with respect to responding to client violence. This is preventative or administrative in nature, and it includes recommendations for policy inclusion. Precautions for field engagement and safety buddies are also included
1.3	Safety committees	The necessity, role, or need for a safety committee in a social work organisation to address client violence
1.4	Safety training	The necessity of social worker training and the types of training that should be considered
**2**	**Office safety**	**The process of ensuring that employees and visitors are safe while at work**
2.1	Arriving at work	Processes for ensuring safety from the moment the social worker entered the employment building's premises
2.2	Preparation for clients	When preparing for client meetings, the social worker should have procedures in place to ensure safety
2.3	Creating safe interview settings	Procedures and approaches that can be used to ensure client safety during office interviews
2.4	Security in the office	Physical methods that could help to improve overall office safety
**3**	**Field visits**	**Being safe while rendering services in the field. Includes all aspects from the time when the social worker leaves the office, until the time of their safe return**
3.1	Planning a visit	The tasks conducted by the social worker prior to conducting field visits
3.2	During a visit	The safety considerations that social workers should keep in mind from the time they enter the field until they leave. This may include aspects that some may regard as “common sense,” as well as some crisis intervention strategies
3.4	After a home visit	Safety precautions once the social worker leaves the service rendering area and returns to the office
**4**	**Transportation**	**While transporting a client between two points, safety risks must be considered. The condition of the vehicle is also important when considering these risks**
4.1	Transporting clients	Requirements of general safety when transporting clients, with a special focus on conditions relating to children
4.2	Assessment at pick-up	Factors to consider when a social worker picks up a client for transportation. There is also some safety advice to ensure that the transportation process is as safe as possible
4.3	Travelling to site	Precautions that the social worker should take while travelling to the service delivering site
4.4	Vehicle condition	The general safety conditions of the transportation vehicle are specified, safety checks of the vehicle are included
**5**	**After an incident**	**Anything that happens following a client violence incident is included in post-incident protocols, which includes, but is not limited to, investigations and support**
5.1	Reporting	Reporting is the act of disclosing details about a client violence occurrence. The motivation or practices supplied by organisations to improve this process fit into this category
5.2	Post-incident protocols	Anything that happens following a client violence incident is included in post-incident protocols, which includes, but is not limited to, investigations and support

**Table 2:** Themes, theme definitions, and relevant codes

No	Themes	Theme definitions	Codes (Refer to Table 1)
**Theme 1**	**Employers inspired a culture of safety and security in the work-environment**	**To protect social workers from client violence, employers provided guiding principles, attitudes, and key determinants to aid in the coordination of plans, goals, and policies**	1.1; 1.2; 1.3; 1.4; 2.3; 2.4; 4.2; 4.4; 5.1; 5.2
Subtheme 1 (a)	Protecting social workers from post-incident trauma	Following acts of client violence, employers implemented protocols to manage the incidents and to protect social workers from additional trauma	1.1; 1.2; 5.1; 5.2
Subtheme 1 (b)	Continuous training contributed to improved skills	Social workers were provided with ongoing training to empower them to deal with client violence	1.1; 1.3; 3.2; 5.2
**Theme 2**	**Social workers prioritised their safety by using their clinical skills**	**The importance of clinical social work skills in dealing with client violence is underlined**	**1.2; 1.4; 2.2; 2.3; 3.1; 4.3; 3.2; 3.3; 4.1; 4.2; 4.4**
Subtheme 2 (a)	A thorough risk assessment was encouraged	Social workers were motivated to undertake holistic risk assessments to identify circumstances where client violence may occur	1.1; 1.2; 2.2; 3.1; 4.3; 3.2; 4.1; 4.2; 4.4
Subtheme 2 (b)	Transparent communication between the social worker and the client	Clear communication is a crucial element in managing client violence	2.3; 3.1; 3.2
Subtheme 2 (c)	Social workers reflected on their own vulnerabilities	In terms of client violence, psychological readiness, self-awareness and study of vulnerabilities are effective preventative methods	2.1; 3.1; 4.3
**Theme 3**	**Actively implementing initiatives to ensure the safety of social workers**	**Initiatives that led to improved safety during office work, field visits and safe travelling**	**1.2; 1.3; 1.4; 2.1; 2.2; 2.3; 2.4; 3.1; 3.2; 3.3; 4.1; 4.2; 4.3; 4.4; 5.2**
Subtheme 3 (a)	Logistical strategies to ensure safety	Logistical solutions for supporting social workers in a time-efficient manner are investigated	2.3; 2.4; 3.1; 3.2
Subtheme 3 (b)	Safety strategies while travelling	Specific measures for assisting social workers while on the road are identified	2.1; 4.3; 3.2; 3.3; 4.2
Subtheme 3 (c)	Improve safekeeping by using new technology	Modern technology was used to help protect social workers from client violence	1.2; 2.4; 3.2; 4.2; 4.3
Subtheme 3 (d)	Ensure safety by involving trusted others	When the social workers thought they were in danger, they involved trusted others	2.3; 2.4; 3.1; 3.2; 4.2
Subtheme 3 (e)	Interaction between the organisation and the social worker	The overall safety of social workers is supported when there is communication between the social worker and the employer	2.3; 2.4; 3.1; 3.2; 3.3; 4.3;

**Table 3:** Code frequencies

No	Code Name	Theme 1	Theme 2	Theme 3	Total
1.1	Data management	23 (92,0%)	02 (8,0%)	-	25 (100,0%)
1.2	Policies	41 (89,1%)	04 (8,7%)	01 (2,2%)	46 (100,0%)
1.3	Safety committees	17 (89,5%)	-	02 (10,5%)	19 (100,0%)
1.4	Safety training	42 (54,5%)	31 (40,3%)	04 (5,2%)	77 (100,0%)
2.1	Arriving at work	-	06 (40,0%)	09 (60,0%)	15 (100,0%)
2.2	Preparation for clients	-	15 (93,8%)	01 (6,2%)	16 (100,0%)
2.3	Creating safe interview settings	02 (3,2%)	05 (7,9%)	56 (88,9%)	63 (100,0%)
2.4	Security in the office	10 (19,2%)	01 (2,0%)	41 (78,8%)	52 (100,0%)
3.1	Planning a visit	01 (1,1%)	38 (42,2%)	51 (56,7%)	90 (100,0%)
3.2	During a visit	01 (1,0%)	42 (43,3%)	54 (55,7%)	97 (100,0%)
3.3	After a visit	-	05 (29,4%)	12 (70,6%)	17 (100,0%)
4.1	Transporting clients	01 (2,6%)	15 (39,4%)	22 (58,0%)	38 (100,0%)
4.2	Assessment at pick-up	-	16 (61,5%)	10 (38,5%)	26 (100,0%)
4.3	Travelling to site	-	17 (44,7%)	21 (55,3%)	38 (100,0%)
4.4	Vehicle condition	01 (4,3%)	04 (16,6%)	19 (79,1%)	24 (100,0%)
5.1	Reporting	17 (100,0%)	-	-	17 (100,0%)
5.2	Post-incident protocols	41 (97,6%)	-	01 (2,4%)	42 (100,0%)

### Theme 1: Employers inspired a culture of safety and security in the work environment

**Fig. 3:**
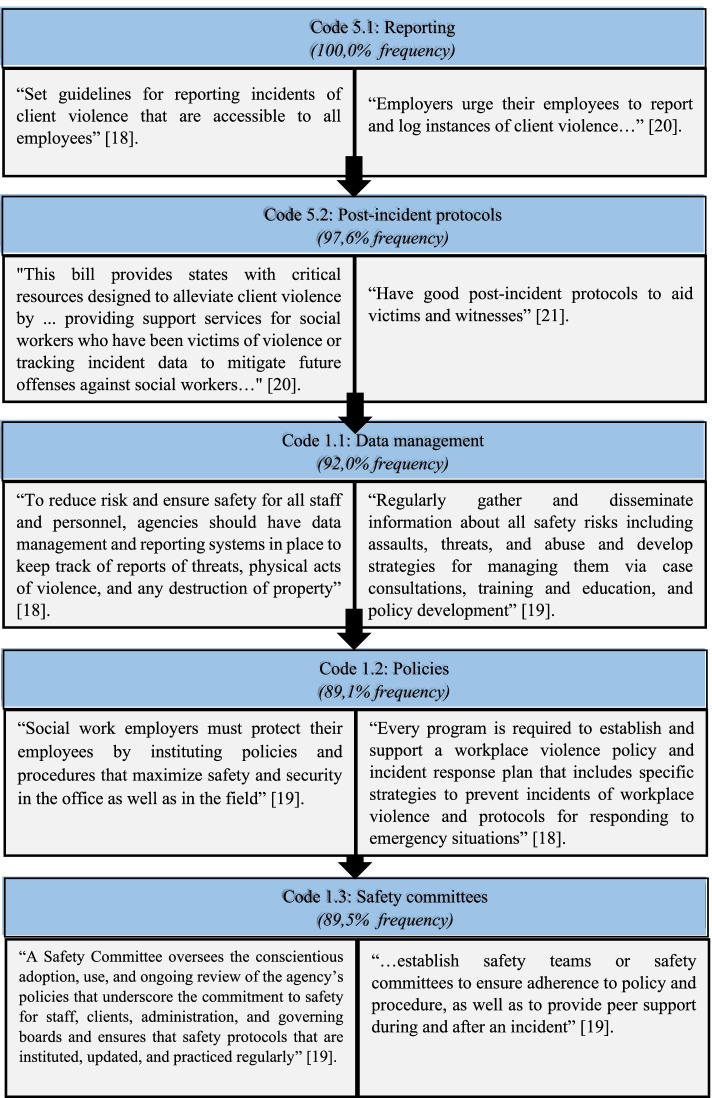
Codes and quotations related to Theme 1 [18–21]

This theme developed as overlapping guiding principles, attitudes and key determents that aided the coordination of policies and guidelines to safeguard social workers, were clustered. Significant codes included data management, policies, reporting, safety committees, safety training and post-incident protocols and all inspired a culture of safety and security in the work environment. Given the interconnectedness of these codes, Fig. 3 shows the five most frequent codes in relation to the main theme:

Table 4 explores the subthemes, followed by a comprehensive discussion of Theme 1 and its subthemes.

**Table 4:** Subtheme 1(a) and 1(b)

**Subtheme 1 (a):** Protecting social workers from post-incident trauma	
**Related codes**	**Supporting quotes**
Data management	“*To reduce risk and ensure safety for all staff and personnel, agencies should have data management and reporting systems in place to keep track of reports of threats, physical acts of violence, and any destruction of property*” [22]
Policies	*“A policy stating any act of workplace violence is prohibited and is cause for discipline measures*” [18] “*Providing prompt assistance to the employee*” [19]
Reporting	*“To reduce risk and ensure safety for all staff and personnel, agencies should have data management and reporting systems in place to keep track of reports of threats, physical acts of violence, and any destruction of property”* [19, 22]
Post-incident protocols	*“All workers, including supervisors and management, should be encouraged to discuss safety concerns with their staff and supervisors should offer time for their employees to hold conversations regarding safety*” [18] *“When responding to incidents, employers should provide immediate aid and support to any injured employees and conduct the measures needed to prohibit any other individuals from being injured*” [19]
**Subtheme 1(b):** Continuous training contributed to improved skills	
**Related codes**	**Supporting quotes**
Data management	*“Regularly gather and disseminate information about all safety risks including assaults, threats, and abuse and develop strategies for managing them *via* case consultations, training and education, and policy development”* [19]
Safety committees	*“…provides ongoing proactive risk assessment that identifies line staff at risk for violence, precarious settings and working conditions, as well as orientation and in-service training on practices that can reduce or minimize or eliminate factors associated with elevated risk*” [19]
During a visit	*“Utilize your learned clinical skills to deescalate the situation, remain calm, show respect, and never make promises you cannot keep*” [23] *“Keep it from escalating; use your attained skills to stay calm and listen attentively*” [24]
Post-incident protocols	*“This bill provides states with critical resources… facilitate safety training programs, provide support services for social workers who have been victims of violence, or track incident data to mitigate future offenses against social workers, among other important uses”* [20]

The analysis of policies and guidelines [18, 19] found that agencies should produce, cultivate and manage an organisational culture that promotes safety and security for their employees. Promoting a proactive approach to workplace violence prevention is one of the most effective strategies to maintain a safe workplace environment [18]. Safety policies and procedures can be developed in consultation with frontline workers and then disseminated to all employees or even members of the general public for review [18]. Following the completion of a safety policy, the employers and employees' oral and written commitments can support effective implementation [1, 19], whereafter a data-management system can be developed.

A data-management system (DMS) is a system or database set up by an employer to keep track of all instances of workplace violence (including client violence) [18, 19]. The purpose of a DMS is to reduce risk and promote the safety of all staff and personnel [18, 19, 22, 25]. A functional DMS can be used to detect protocol vulnerabilities or gaps that might have facilitated or contributed to, or failed to prevent, incidents of client violence. A DMS is only effective if data are recorded continuously and systems are updated on a regular basis [26]. Social workers must be encouraged and supported to report incidents of client violence. The NASW [19] states that data should be recorded on the type of incident, location, pervasiveness, and occurrence. If records are available, they must also include the names of all parties involved, the date, time, and location of the incident, a description of the event, and the extent of any injuries [18].

Themes identified during the document analysis process, emphasizes the significance of post-incident practices in the unfortunate event of client violence. Such procedures may include debriefing workers and witnesses as soon as possible after the occurrence [18, 19]. This should be followed by making support services available, such as counselling and referral to employee assistance programmes and other resources such as technical and legal support [18–20, 27, 28]. Assessment of medical need and providing medical treatment should be part of post-incident protocols. NASW [19] and Stowe [18] suggest the possibility of financial compensation. Employees may have to rely on their own internal resources for whatever they include in their protocol. The majority of the literature emphasises that after a client violence incident, employers ought to be vigilant with respect to their social workers' stress levels. The long-term effects of client violence can be mitigated by revising routines and addressing the caseload distribution of the affected social worker [1, 19].

Who is responsible for safety policies, DMS, and post-incident processes in the already overburdened South African social work context? The NASW [19] highlighted that safety committees should be established for all social work AODs. The purpose of these committees is primarily to prevent client violence and to ensure the application of policies and development of incident response methods [18]. They are often responsible for establishing, updating, and enforcing policies regarding safety and tracking all known and perceived threats in the workplace [5, 18]. In addition to risk assessments, the role provides orientation and in-service training on best practices, reducing or minimising risk factors for social workers, as well as identifying and investigating physical measures and technology contributing to and promoting safety [19]. This type of committee meets quarterly or monthly and records its activities [5, 18, 25].

### Theme 2: Social workers prioritised their safety by using their clinical skills

The NASW [29] describes clinical social work as the professional application of social work theory and methods for the diagnosis, treatment, and prevention of psychosocial dysfunction, disability, or impairment, including emotional, mental, and behavioural disorders. Referring to clinical skills, the following is noted: “*Drawing on knowledge of systems theory, person-in-environment orientation, psychodynamic theory, interpersonal dynamics, and family systems, clinical social workers shall be familiar with social, psychological, cultural, and health factors that influence the mental, emotional, and behavioural functioning of the client”* [29].

**Fig. 4:**
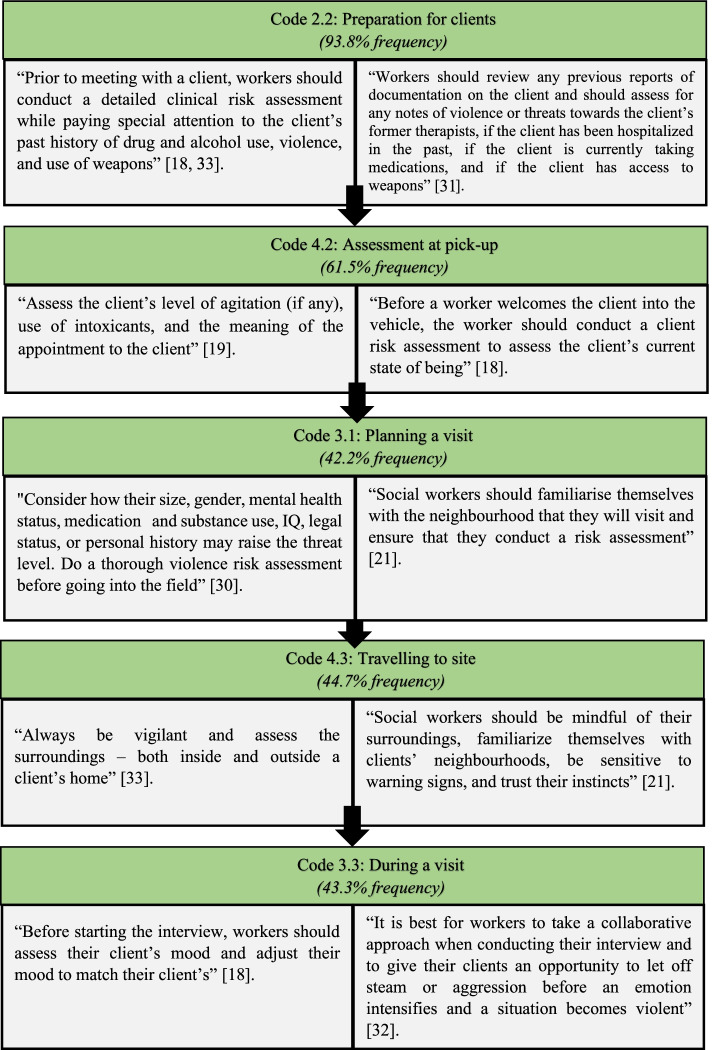
Codes and quotations related to Theme 2 [18, 19, 21, 30–33]

When overlapping clinical social work skills that can be used to address client violence were grouped together, Theme 2 and its related subthemes emerged. Policies, safety training, client preparation, creating safe interview settings, planning a visit, traveling to site, during a home visit, after a home visit, assessment at pick-up, transporting children, and vehicle condition, were all codes that were important in this theme. Based on the interconnectedness of these codes, Fig. 4 shows the five most frequent codes in relation to the main theme:

All subthemes are presented and explored further in Tables 5, 6 and 7. A concise discussion follows each table.

**Table 5:** Subtheme 2(a)

Subtheme 2 (a): A thorough risk assessment was encouraged	
**Related codes**	**Supporting quotes**
Policies	*“If the client is assessed to be unsafe to transport, or the vehicle is assessed to be unsafe to operate, agency policies should prohibit the social worker from transporting the client”* [19]
Planning a visit	“*It is important to familiarize yourself with the clients’ file prior to the home* visit” [30] *“Speaking to the client prior to the visit helps the worker retrieve important information regarding their client’s situation, any safety precautions that may need to be taken according to the client, or whether or not their client’s situation has changed*” [21]
During a visit	*“Throughout the visit, workers should be on the lookout for dangers and trust their gut feelings*” [18] *“If a student hears a heated argument from inside the house or apartment, the student may decide to re-schedule the visit or call to assess the situation before entering the dwelling”* [34]
Transporting children	*“Assessments of the child’s state of being should be conducted on an on-going basis throughout the trip*” [26] *“Workers should check to see if there are any items in the car that could be used as a weapon and if so, should remove them immediately”* [19]
Vehicle condition	*“…workers should determine if the car is in proper functioning order”* [18] *“If an agency car is being used, workers should take time to orient themselves with how to operate the car including how to turn on the high beams and emergency flashers”* [32]

**Table 6:** Subtheme 2(b)

Subtheme 2(b): Transparent communication between the social worker and the client	
**Related codes**	**Supporting quotes**
Creating safe interview settings	“*Set clear limits, on what you will not tolerate, i.e., intoxication, violence, 51A*” [26]
Planning a visit	“*Workers may wish to notify their client by phone that they will be visiting their home and advise them of the purpose of the visit, if possible*” [21] “*Try to schedule the appointment by telephone or letter so that the client will know to expect you and be prepared”* [31]
During a visit	*“When introducing themselves, workers should clearly state who they are, and why they are there*” [18]
Combined codes	“*All employees must wear their ID badges when on duty, both in the office and in the field”* [18]

**Table 7:** Subtheme 2(c)

Subtheme 2(c): Social workers reflected on their own vulnerabilities	
**Related codes**	**Supporting quotes**
Arriving at work	*“… refrain from talking to unknown persons when walking into their workplace”* [24]
Planning a visit	“*If you are highly allergic to certain domestic animals then you should take that into account before conducting home visits. Many clients live with cats, dogs and other pets”* [31] “*Lack of experience or appearing timid, vulnerable, lost, or confused can contribute to violence*” [19]
Travelling to site	“*Workers should refrain from providing those that are unfamiliar with their name, street address, and information regarding where they work. If they encounter anyone as they walk to their client’s home, workers should keep normal, confident eye contact*” [18] “*Limitations of cell phone coverage in areas that social workers may visit*” [19]

Field visits should be carefully planned to include a thorough risk assessment [18, 35]. Planning and risk assessment should consider client factors such as a history of violence, substance abuse, mental illness and the availability of weapons; environmental factors such as high risk events, neighbourhood risks, access and network coverage; worker vulnerabilities such as working alone, visible physical conditions and lack of experience; and factors pertaining to the type of work activities such as removal of a child in need of protection [19, 31, 36]. In preparation for an unexpected event to occur, it is recommended that workers design a safety plan detailing what to do in certain situations before embarking on a visit [31].

Newhill [32] warns that social workers often see home visits as a general concern and an attempt to help, but that clients may observe it as a threat and act accordingly. It can therefore be helpful to notify clients prior to home visits and inform them of the purpose of the visits [18, 19, 30, 31]. The Simmons School of Social Work [34] and Victor [31] state that social workers should continuously assess situations and rather terminate visits when they feel threatened. Quinn and Mason [26] point out that the following home visit scenarios should be rated as high risk: when the person with whom the appointment was scheduled is not present on arrival; when too many persons are on the premises; when too much activity is going on or when obvious weapons or substances can be seen.

Before embarking on any field visit, an inspection of vehicles should be part of the risk assessment to ensure that it is in proper functioning condition and safe to use [19, 26, 36, 37]. This includes ensuring a full tank of gas, enough water, working head- and taillights, a working horn and emergency safety equipment like jumper cables, road flares, and a spare tyre with a jack, a flashlight and a first aid kit [19, 21, 24].

When transporting clients or children, social workers should continuously assess if the client is displaying signs of aggression, if the client is under the influence of drugs, and if the client is in possession of a weapon [18, 19, 26]. In addition, social workers should ensure that the vehicle is free from any objects that can be used as weapons (for example, pens, pencils, magazines, books, handheld devices, hot beverages) before allowing clients into their vehicle [19]. If either the client is assessed to be unsafe to transport, or the vehicle conditions are assessed as unsafe to operate, AOD policies should prohibit the social worker from engaging. Law enforcement could be called to transport the client in such instances [18, 26].

Effective communication techniques are part of clinical skills, and transparent communication between social workers and their clients can lessen the risk of client violence. When a social worker contacts a client during the planning or preparation phase, it is important to identify him-/herself and explain the purpose for involvement to ensure that effective engagement can commence [21, 31]. This might help a client feel at ease with the process and establish a relationship with the social worker [26].

Social workers are urged to wear identity badges when they work in the office, out in the field, or when they are transporting clients [18, 19, 24]. When meeting a client for the first time, the social worker should again introduce him-/herself and describe the aim of the consultation [24, 26, 32, 36]. Clear communication practices are considered as mutually beneficial in considering the safety of social workers and clients.

Clinical qualities such as psychological preparedness and self-awareness are crucial, and social workers are urged to reflect on their own vulnerabilities. Despite open communication, documents included in this study advise against disclosing personal information to clients, such as addresses and phone numbers [18, 24]. Social workers should also be conscious of their surroundings and refuse unsolicited help from strangers [24].

It is critical to include previous experience with client violence, as well as any bias or stereotyping that leads to over- or underreaction to safety concerns, during the assessment and planning process [19]. Practical considerations such as allergies, the probability of contracting diseases, and even the appearance of social workers should all be part of self-awareness and introspection.

### Theme 3: Actively implementing initiatives to ensure the safety of social workers

**Fig. 5:**
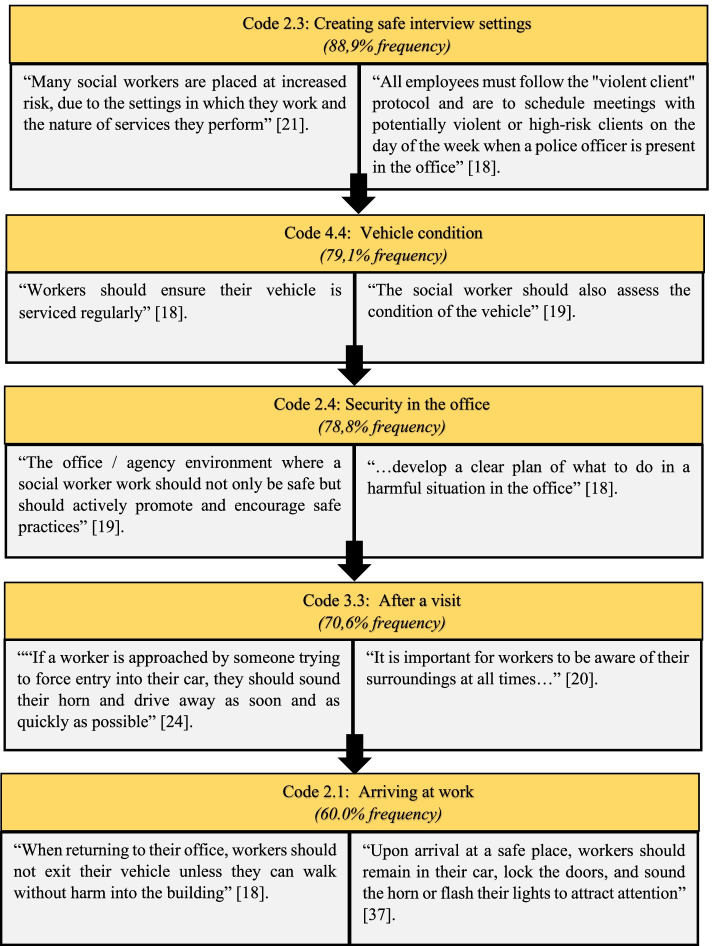
Codes and quotations related to Theme 3 [18–21, 24, 37]

This theme is highlighted in the research because it includes practical initiatives that can help safeguard social workers against client violence when at work, on field visits, and while traveling. Since all of the codes were part of this theme and its subthemes, Fig. 5 depicts the five most common codes in relation to the main theme, followed by each subtheme's unpacking.

Tables 8, 9, 10, 11 and 12 is used to extract subcodes relevant to Theme 3, followed by a description of each.

**Table 8:** Subtheme 3(a) related to Theme 3

Subtheme 3(a): Logistical strategies to ensure safety	
**Related codes**	**Supporting quotes**
Creating safe interview settings	*“Leave door open during session, if there are others around who could come to your aid*” [26] *“Ordinary objects can be easily used as a weapon by someone in an angry state”* [19]
Security in the office	*“Know the location of all safety aids and systems*” [21] *“Secure entrances to employees’ workspaces that are separate from public spaces*” [19]
Planning a visit	*“Before departing on a field visit, workers should ensure they are wearing appropriate clothes and shoes that allow for ample movement”* [18] *“If a visit outside of the office is required, and the potential for danger exists, workers may work to schedule their client meeting in a public place”* [31]
During a visit	*“Keep a clear path to the exit door”* [36] *“Do not enter an elevator with people who are suspicious-looking or make you feel uncomfortable in any way”* [31]

**Table 9:** Subtheme 3(b)

Subtheme 3(b): Safety strategies while travelling	
**Related codes**	**Supporting quotes**
Arriving at work	*“Visually check parking lot when you arrive at work”* [24] *“When exiting the vehicle and approaching the office building, workers should scan the environment and check the outside of the building for safety before entering*” [18]
Travelling to site	*“All personal items should be left in the car or trunk of the car and hidden from public eyesight during travel”* [36] “*Ask to see identification of anyone stopping to assist you*” [30]
During a visit	*“Keep keys with you and lock doors when no one is in car”* [26] *“During the interview, workers are encouraged to keep their car keys and a functioning cell phone on them in a place that allows those items to be reached easily*” [22]
After a visit	*“Once the visit has concluded, workers should prepare to approach their vehicle by having their keys ready to unlock their vehicle*” [21] *“Scan front and back seat and floor before getting in*” [18]
Transporting clients	*“Know the proper use and installation of a child safety seat that is appropriate for the child’s age and size”* [19] *“Clients should be seated behind the passenger seat”* [18]

**Table 10:** Subtheme 3(c)

Subtheme 3 (c): Improve safekeeping by using new technology	
**Related codes**	**Supporting quotes**
Policies	“*Each cell phone or laptop is connected to a GPS system online alerting management of their worker’s whereabouts at all times”* [18]
Security in the office	*“The ability to alarm others of an emergency or need for emergency assistance through the use of technology*” [18]
Travelling to site	**“***Mobile safety devices may incorporate GPS and/or audio/video recording…”* [19]
During a visit	**“***Keep GPS-enabled mobile phone applications activated at all times while in the field”* [19] *“In general, a cell phone is very useful for students doing home visits”* [30]
Transporting clients	*“Use a mobile phone app such as BSafe that by touch or voice activates an SOS alert, including your location and live GPS tracking*” [30]

**Table 11:** Subtheme 3(d)

Subtheme 3 (d): Ensure safety by involving trusted others	
**Related codes**	**Supporting quotes**
Creating safe interview settings	*“If a worker is concerned about the mood and actions their client is displaying, they should inform their co-workers or supervisor, request a colleague to sit in on the interview, or keep the door ajar to allow others to provide assistance if needed”* [18] *“Ask if anyone is available to sit in, at least until you assess the situation”* [26]
Security at the office	*“Establish an employee saferoom, and use a buddy system, a tag-team, or ALERT device”* [21] *“Signal a co-worker or supervisor that you need help…”* [24]
Planning a visit	*“When visiting a high-risk client, or a potentially violent client, it is crucial for workers to utilize a buddy system in order to be accompanied by a colleague during the visit”* [31] *“…the ability to go out as a team in a potential unsafe environment”* [23]
During a visit	*“Depending on the case and any confidentiality issues, you can possibly get a client’s trusted family member involved and conduct a joint home visit with that person”* [31] *“Implement a ‘buddy system’ in the event of an emergency which requires at least two workers to conduct the client visit together”* [18]
Transporting clients	*“Use a ‘buddy system’—that is, have a second social worker in the vehicle when transporting a client”* [19] *“If a colleague is available to accompany the worker, a buddy system should be utilized to ensure safety when transporting clients”* [18]

**Table 12:** Subtheme 3(e)

Subtheme 3 (e): Interaction between the organisation and the social worker	
**Related codes**	**Supporting quotes**
Creating safe interview settings	*“Once appointments are made, workers should share their schedule with their co-workers…”* [22] *“A social worker should never see a potentially dangerous client alone without someone else in the agency knowing about the client, the appointment time and the location of the appointment”* [30]
Security in the office	*“Be prepared with code words or phrases that alert your employer and colleagues to an emergency or a dangerous situation*” [34]
Planning a visit	*“Be sure to inform your supervisor and another colleague of your whereabouts”* [31] *“Any changes in appointment field visits should be reported to the worker’s supervisor or agency representative”* [19]
Travelling to site	*“Calling the office before entering a client’s home”* [18]
During a visit	*“Keep emergency contacts on speed dial”* [19] *“Agree on and use “code” words or phrases to help social workers convey the nature of threats to their managers or colleagues”* [19]
After a visit	*“Following each visit, the social workers should report back to their supervisor or designated agency representative when the meeting is concluded or as soon as it is safe to do so*” [19]

### A sense of safety in the office

According to Nordesjö [1], safe working conditions are insufficient if social workers refuse to accept responsibility for implementing the measures that have been offered. Social workers should be aware of the locations of all safety aids and systems and be trained on accessing and implementing them [21]. Apart from technological measures (which are explained in more detail further on), practical features such as well-lit hallways, visually open meeting places and secure entrances to employee workspaces can all help to create a sense of security in the workplace [19, 25].

### Creating secure interview environments

Interview rooms and offices where consultations with clients are held should have secure access and be kept separate from waiting areas and public spaces [18, 19]. It is, nonetheless, beneficial if the room is visible to other AOD employees [30]. Contemplate what is in the room, whether there are many exits, and where each individual might sit when deciding where the meeting should be conducted [34]. Furniture can be strategically placed to allow the social worker to sit closest to the entrance for easy exit while also facing the door to monitor access [18, 24, 25, 38]. Furthermore, Hardy [38] and the Simmons School of Social Work [34] urge AODs to leave adequate space in interview environments that no individual will feel trapped, which may escalate the risk of violence.

While many social workers prefer a closed office door during a client meeting to preserve client privacy, it is recommended that professionals leave the door partially or entirely open [22, 25]. This can indicate to the client in a nonverbal way that others are close and listening or observing, reducing the likelihood of violent behaviour. Another important strategy for social workers is to keep the presence of potential weapons, such as a fire extinguisher, moulded plastic chairs, office décor, staplers, paper weights, or sharp items, such as a letter opener or pair of scissors, to a minimum [18, 19, 22, 24, 25, 30, 37, 38].

### Planning for site visits

It is critical to plan ahead of time to ensure that logistical procedures are in place to keep social workers safe. Social workers should determine whether client interviews can be done in the office, or if a home visit is required. If the risk of danger appears to be severe, the social worker may also try to arrange for the meeting to take place in a public place [31]. If a home visit is necessary, it should be scheduled as early in the day as possible to ensure that social workers could get assistance in emergency situations [22]. In addition, social workers should avoid arranging too many home visits in one day to protect their own vulnerability [32].

Wearing appropriate attire makes it easier to move around and makes social workers less likely to be targeted [30]. When working in the field, it is essential to wear suitable clothing and shoes, considering social workers are frequently required to walk, stand, or climb stairs [21, 24, 31]. These clothing may be semi-formal in nature, yet still project a professional image. Jewellery, lanyards, keychains around necks, ties, or earrings should be considered a safety hazard as clients can easily grab them and cut off social workers' breathing [21, 26, 36, 38].

### Protecting social workers at clients' residences

Literature recommends several strategies to protect social workers at clients’ residences, as these are client’s comfort zones. Social workers should take care to respect clients from the time of arrival by not slamming vehicle doors or strolling on client lawns, but rather knocking or using doorbells appropriately [24, 31]. Although client privacy is emphasised, it is good if social workers enter the home through a door that is visible from the street and position themselves between the client and the exit [18, 21, 24, 31, 36]. If given a choice, social workers should avoid consulting with the client in the kitchen where possible weapons (knives and boiling water) are kept, the bedroom where clients are most comfortable, or any secluded, unlit spaces such as the basement [18, 24, 36].

Stowe [18] and Taylor [37] recommend that the social worker waits to be invited to sit down and then choose a seat that is inviting and non-confrontational. It is best to sit in a chair with a straight back since it's easier to get up in an emergency [24]. The social worker can scan the home for others present and keep an eye on the front door if anyone enters the home [18]. If there are too many significant others present, or if weapons or substances are prevalent, social workers should end the interview and leave the client's home immediately [26]. It is important that social workers never put themselves in situations that are dangerous to them.

During the interview, some clients could have blaring televisions or radios, and Stowe [18] recommends asking them to turn it off. If a client has pets in their home, social workers are allowed to request that the pets be restrained during the visit [31]. If a dog approaches a social worker, they should stand tall and not move, allow the dog to approach them, avoid facing their back to the dog, use loud, strong commands, protect their neck and face, and try to give the dog something to chew or play with [18, 36].

Several strategies have been identified to protect social workers while travelling. Aside from assessing the vehicle's condition, literature suggests that valuables and personal goods should not be displayed when visiting sites [18, 21, 34, 36, 37]. Instead, it is recommended to keep them secured in the trunk and to carry a modest amount of cash, keys, and a cell phone in a waist pack, a compact cross-body bag, or on the individual’s person [26]. When travelling, social workers should be mindful of their surroundings and travel in well-lit, easily accessible areas. When arriving at a destination, it is recommended that vehicles be parked in the direction of departure and in a manner that provides for simple access and that prevents the possibility of being trapped or blocked in by other vehicles [18, 21, 22, 29, 37].

While a social worker conducts a consultation, their car keys should be easily accessible and when the consultation is concluded, workers should approach their vehicles with their keys ready to unlock [21, 35, 37]. Stowe [18] advises that the social worker should inspect the front seat, back seat and floor, before getting into the vehicle. If workers perceive they are being followed while traveling, they should immediately go to the nearest police or fire station. An alternative option is an open gas station or business and to contact the police department [18, 24]. Workers should remain in their vehicles, lock the doors, and sound the horn or flash their lights to attract attention [37]. It is allowed to ask personal identification from anyone who offers to help [21].

Similar strategies are applicable when arriving back at the office. Social workers are advised to remain in their vehicles, visually scan the parking lot for unidentified vehicles or individuals and record the license plate number of any unusual vehicles [18]. Additionally, social workers should scan the area and inspect the outside of the building for safety hazards before approaching the office building [24].

Other safety strategies during travel may include safety measures for the specific client population: when transporting a young child, child safety locks should be activated and age-appropriate car seats should be used; when transporting an adult, clients should sit behind the passenger seat [18, 19, 26]. It is emphasised that social workers should at all times adhere to the state’s regulatory requirements during the client transportation process.

Literature emphasises the use of modern technology to protect social workers against client violence. If an AOD’s policies allow for it, technology can be used at the office, on field visits, and when traveling. Assuming funds permit, all office entrances can be fitted with coded access, and AOD reception rooms can be equipped with fortified glass to safeguard personnel [18]. Security cameras or metal detector screenings are examples of other secure entry and access possibilities [19]. The majority of the research, however, advocates for alarm systems [1, 19, 30] or panic buttons that can notify others to a safety threat [18, 19, 22, 23, 37]. The panic button is frequently disguised or actuated via key fobs or mobile devices and it can be linked to public safety departments (police, emergency rooms, fire departments) [20, 38].

According to the NASW [19], personal safety equipment that includes silent panic buttons, a GPS, or audio/video monitoring, can be advantageous when traveling. When conducting field visits, pre-programmed smartphones with GPS-enabled applications can also be useful [19, 26, 30, 31].

The National Association of Social Workers introduced the concept of a *safety buddy*. A safety buddy is someone who collaborates with the social worker to preserve safety and has readily available resources (phone numbers, escape routes) in the event of an emergency. Safety buddies are present during consultations, but their role is to observe for indicators of violence so that they can intervene if necessary [25]. These buddies could be co-workers, employees from other AODs involved in the case, law enforcement officers, or even clients. Colleagues or team members are the most practical to implement, and social workers are encouraged to pair up and conduct home visits on the same day [22, 24, 26, 31, 36, 38]. In some instances, a client can serve as a safety partner and provide guidance on local safety concerns [21]. Individual AODs may have policies that clearly stipulate the roles and responsibilities of the safety buddies.

Clear communication practices between the social worker and the employer can ensure that someone will check in and follow up in event of an emergency, or if a social worker does not return when expected [37]. It requires the social worker to provide their supervisor or colleague with a schedule (consisting of home visit addresses and appointment times), estimated time of arrival and departure times (duration of visits), vehicle information (license plate number, make, model, colour) and information on how to reach them [19, 22, 24, 31, 32, 35, 36]. A sign-in/sign-out system may be beneficial for the tracking task.

Stowe [18] and Hardy [38] recommend calling the office before entering a client's house and notifying them as soon as the consultation is completed; these protocols established by the AOD should always be followed. Any changes in scheduling should be communicated to the appointed AOD representative, if possible [19].

## Summary and Conclusion

This document analysis involved an extensive and time-consuming research procedure, resulting in an extensive data set, but the ATA was found a useful approach. It provided a comprehensive yet flexible structure that includes reflexivity, minimising potential bias, enhancing rigor, transparency, and replicability, and strengthening the processes through which researchers gained theoretical insights. The ATA established a good understanding how client violence is addressed on a global scale by identifying three main themes and a number of subsequent subthemes.

Documents that have been identified to form part of the analysis, provided important pointers for employers on how to instil a safety culture in the workplace, how social workers can use their clinical abilities to contribute to their safety, and specific measures to enhance and maintain safety while providing services in the office, visiting sites, or traveling. Examining these practicalities provided valuable data that can inform policy development processes to enhance and maintain the safety of South African social workers.

### Limitations and Recommendations

There are limitations to any scientific study, and this one is no exception. One limitation is the potential of 'biased selectivity.' The National Association of Social Workers in the United States was involved in the study's design since the inception. The NASW provided a large number of the documents used in this investigation. The available (chosen) documents in an AOD environment are likely to be linked with AOD policies and procedures, as well as the agenda of the AOD's principals. It should also be highlighted that some of the materials examined were prepared for reasons other than research; they were created without regard for a research aim and may not be scientific in nature. However, the guidelines supplied by social work AOD on a global scale is usually provided in this form and therefore became a critical aspect of the study's objective.

The research yielded information on how social workers can be protected from client violence and the findings can be used to inform policy development in South Africa. At the macro, mezzo, and micro levels of service delivery, the government can adopt policy measures to protect social workers from client violence and implement ways to limit, diminish, and eliminate client violence. The SACSSP should take note of the data collected and assist in the development of a reporting system for social workers who have been exposed to client violence occurrences. Training institutions should also take action by providing educational courses to provide social workers with the knowledge and skills they need. Finally, international and national social work researchers should continue to focus on client violence research, whether as individuals or as interdisciplinary partners.

## Supplementary Information

**Additional file 1.** ATA data analysis AM. 

**Additional file 2.** Data extraction and analysis.

**Additional file 3.** Finelized codebook. 

**Additional file 4.** List of included literature.  

**Additional file 5.** Research plan and ATA process followed 1.

**Additional file 6.** Research plan and ATA process followed 2.

## Data Availability

All data generated or analysed during this study are included in this published article [and its supplementary information files].

## Abbreviations

AOD: Agencies / organisations / departments
ATA: Applied thematic analysis (ATA)
CWLA: Child Welfare League of America
DCF: Department of Children and Families
DMS: Data-management system
NASW: National Association of Social Work
NWU: North-West University
