# Supplementary material for: International guidelines to inform policy development to address client violence in South Africa: an ATA-document analysis
Source: BMC Health Serv Res. 2022 Aug 12;22:1025. doi: 10.1186/s12913-022-08196-8 (PMC9373364; doi:10.1186/s12913-022-08196-8)
Supplement: Supplementary file 1 — Additional file 1. ATA data analysis AM. [file 12913_2022_8196_MOESM1_ESM.pdf]

Client Violence and Social Worker Safety

Lunchtime Series Webinar

These notes have been taken from a Webinar presented by Dr. Newhill that you, as a NASW member, may take for free: http://www.socialworkers.org/ce/online/lunchtime/lcourses/home.aspx. NASW, OH chapter has also added comments in this overview obtained from findings from formal and informal data collection in regards to workplace safety over the past two years.

About the Lunchtime Series

These live monthly hour long teleconferences are exclusively for NASW members. The Lunchtime Series highlights the latest social work issues being addressed by NASW. Besides a live teleconference, members can access the transcripts and audio/pod cast component of the teleconference 24/7 through NASW's Web site. Members may also earn continuing education units (CEUs) upon successful completion of an online test following each teleconference.

The Lunchtimes Series Webinar presenter on Client Violence and Social Worker Safety is Dr. Newhill, associate professor at the University of Pittsburgh, who has over 10 years of psychiatric emergency and inpatient practice experience. Dr. Newhill has conducted research on risk assessment and social worker safety and is the author of the **Client Violence in Social Work Practice,** Prevention, Intervention and Research. She currently chairs the NASW mental health specialty practice section.

In her workshop Dr. Newhill stated: "Preventing client violence is one of the best things that we can do for our clients because as (Andrew Slayable), psychiatric emergency psychiatrist commented one time, once a client strikes you, from that point on, that client is going to be labeled a violent client. Many clinicians won't be willing to work with them." (1) Prevention + management. – w violence affects how clients are treated / help they get.

As we, the NASW-OH Chapter, have heard from social workers there is an increasing concern about violence in the workplace, Dr. Newhill supports this perception stating that she has found a number of indicators that support the perception that there is a rise in physical and verbal violence across settings. - types of violence

What are the causes?

Potential factors discussed by Dr. Newhill are the following:

- We as a society perpetuate violence, where significant societal problems, such as unemployment, poverty, and lack of health and mental health services support violent reactions to those who may become hopeless and angry. emotions that precede violence.

social workers are in a position of power.

- Possibly our role as a social worker, where we have significant power over the client especially in situations where we have a job that involves "interpreting government regulations and mandates and dispensing resources that clients desperately need and sometimes are not able to provide." What about the power we have over parental rights or personal rights and making decisions that distress and anger the client. (decisions on behalf of indv.)
- There are "political issues and policy shifts that have created conditions that increasingly place social workers at risks. For example, as our economy has tanked and our government has cut back on certain types of institutional support that we use to rely on and our clients use to rely on, the number of those needing public assistance and other social services have increased. Budget cuts in our agencies, the ensuing understaffing of social services and rising case loads have also led to increased vulnerability for social workers." (political issues + policy shift; high instance of people)
- A number of indicators also suggest that the settings of violence are varied; there still remain some settings that have a higher risk but don't assume that you are safe just because it has not happened in your workplace. We can no longer assume it only occurs outside of the office, in the home or community of the client. In Ohio we have heard of safety issues from those serving children to the aged, from agency based to community based service providers. (varied settings; 1.6 uncertainty on what settings are safe; 2. Home visits)
- With our current economy and the significant cuts in resources we are seeing social workers carry significant client loads at a rate where the social worker no longer knows their client. Organizations are limiting supports for workers such as providing panic buttons, safety courses, and the ability to go out as a team in a potential unsafe environment are just a few of the impacts. (1.5; support)

An alarming finding from Dr. Newhill was the number of agencies "using male social workers as sort of an informal security force but without providing them with adequate training or hazard pay".

Where to begin to address this issue:

The first place Dr. Newhill suggests to start is with information such as understanding risk factors for violent behavior.

- (1.6) Understand that client violence toward social workers is not a rare event
- (2) The risk varies according to where one works - area
- Male social workers are at a significantly greater risk of experiencing client violence than female social workers - gender of SW
- Experiencing an incident of client violence exacts an emotional toll on the social worker involved (1.6)

- Take a systems approach;
  - Recognize that violence is not a static individual attribute, rather, violence is an attribute of individuals ***within*** certain situations and environmental contexts, i.e. the person-in-the-environment;
  - Always interpret the client's emotional status and behavior within the context of his/her social/environmental system.

violence varies / across contexts

- client focused approach

profile of SW –

- ***Demographic Risk Factors***
  - Young Age
  - Male Gender
- ***Clinical Risk Factors***
  - High Risk Psychiatric Symptoms (delusions, hallucinations, violent fantasies)
  - Personality Features (anger, emotion dysregulation, impulsivity)
  - Personality Disorder (antisocial, borderline)
  - Substance Abuse (especially alcohol)
- ***Biological Risk Factors***
  - Low Intelligence Quotient (IQ)
  - Neurological Impairment
- ***History of violence;***
- ***Social and family history*** (early exposure to violence);
  - Experiencing severe abuse by a parent or other caretaker or being a witness to domestic violence;
  - Being severely neglected or rejected by parent/caretaker;
  - Parental psychiatric illness and/or drug or alcohol abuse;
  - Tacit parental approval of cruelty toward other people or animals.
- ***Work history*** (economic instability, unemployment);
- ***History of psychiatric treatment and/or hospitalization***, especially if involuntary;
- Level and quality of social support

profile of client (1.1)

- Peer pressure from peers who endorse violence
- Influence of popular culture
- Means for violence
- Accessibility of the potential victim

If you have not had training on how to handle a potentially violent client, seek out that training – which may be in a workshop or through the literature. Newhill suggests the following: utilize your clinical skills to deescalate the situation, remain calm, show respect, and never make promises you cannot keep. Above all listen and seek to understand.

training

Everyday Self Defense℠

Personal Safety Tip Sheet for Human Service Workers

I would put this as a new code as ~~suggested~~.

The Basics (1) (1.5)

internal, physiological, psychological.

- Be aware of your surroundings and your feelings.
- Trust your instincts.
- Be assertive (verbally and non-verbally) to protect your safety.
- Take a deep breath to help you remain calm and in control.
- If threatened, assess the situation and decide on a course of action.
- Think about acting, bluffing, or faking compliance to buy time.
- Raise your voice to distract a perpetrator or draw attention to yourself. Scream, yell or talk loudly.
- For de-escalation, use a calm, low voice.
- Use the element of surprise and react quickly if you need to escape.
- If you are unable to escape harm, do what you need to do to survive.
- Use evasion, pushing and deflection as escape techniques. If you have no choice, use your fingers, fists, palms, elbows, knees, and feet as weapons.
- Respect and believe in yourself.

In Your Client's Home (3) (3.3)

- Make sure you understand that you are on their turf – a natural safety dilemma.
- When you schedule a visit, if possible, let the client be your safety partner by letting them advise you about any concerns in their area. Ask them to watch for you as you arrive and when you leave.
- Drive past the home and around the area. Step back and observe the home. Look for its' hiding places, vulnerable points, blocked exits and escape routes.
- Listen at the door before knocking and stand to the side before the door is opened.
- As you enter the home, notice the layout, exits and phones. Position yourself for an easy exit should you need to leave quickly. Avoid the kitchen and other tight quarters.
- Wear comfortable shoes and clothes that allow you to move easily.
- Keep your purse locked in the trunk unless you really need it. Keep keys, a little money, and your phone in your pockets, a small cross-body bag or waist pack (on your person).
- Play "what if" games to mentally prepare for various situations (threats by a client, suggestive comments, stranger threats, and out-of-control family or neighbors).
- Look around and think of what objects could be used as weapons (by you or against you).
- If anything looks or smells out of the ordinary in or around the dwelling, or if you feel uneasy about your situation, leave and call your supervisor or the police/sheriff. Go with your gut!
- Travel with a cell phone. Keep it on and programmed to call 911 for help in any emergency or threatening situation. Two-way radios may be needed in rural settings.
- Take a "buddy" or law enforcement with you on potentially dangerous home visits. Have a safety plan.
- MOST IMPORTANTLY, KNOW YOUR CLIENT! Consider how their size, gender, mental health status, medication and substance use, IQ, legal status, or personal history may raise the threat level. Do a thorough violence risk assessment before going into the field.

On the Job (1) (1.6)

- Tune in to your surroundings and be aware of possible safety threats.
- Use confident body language and verbal skills.
- Know the location of all safety aids and systems.
- Avoid impolite behavior. Be calm, courteous and gracious. Listen more than you talk.
- Identify and avoid potentially dangerous locations or situations, if possible.
- Dress sensibly to allow for comfort and ease of movement.
- Anticipate how you would respond to threatening events and stay alert.
- Attend training on conflict resolution, personal safety, teamwork and stress reduction.
- Know your job's safety plans, grievance procedures, harassment policies and employee rights.
- Know predictors of imminent violence (abusive language, threats, violent history) and that violence rarely strikes without warning.
- Establish an employee safe-room, and use a buddy system, a tag-team, or ALERT device.
- Keep your distance (safe space) from strangers and be aware of boundaries with clients.
- Avoid discussing plans and personal information within earshot of people you don't know.
- Assess possible weapons (books, scissors, clip board, keys, pen, umbrella) you may have at your disposal, or that could be used against you.
- Seek peer support. Ask peers to share their successful reactions to threatening incidents.
- Have good post-incident protocols to aid victims and witnesses.

In the Car (1)

- Make certain your car has gas, water, a spare tire with jack, a working horn, spare change, a flashlight, jumper cables and a first aid kit.
- Ask to see identification of anyone stopping to assist you. Law enforcement too!
- If you have a flat tire and cannot change it, call for help or try to keep driving slowly until you reach a safe stopping point.
- Park for a quick exit and avoid getting blocked in. Use extra caution in parking garages. Scan the garage as you enter and spot the exits.
- Have your car keys in hand as you approach your car. Use the remote panic button if needed.
- If stranded and you accept assistance, pretend that someone else will soon be arriving. Stay alert and prepared to not be victim of a "Good Samaritan" ploy.
- Car hijacking is life-threatening. Consider turning on the flashers, pressing the horn, stopping suddenly, getting out and running.
- If someone approaches your car to force entry, lay on the horn and drive off quickly.
- If you have your windows open, be aware of what's going on around you.
- Keep car doors locked while in or away from your vehicle.
- Scan the area as you approach your parked car, looking around it and checking the floor/back seat.
- If being forced into your car, throw away the keys (distracting the attacker) and run.
- Be careful about what you leave on your seats or dashboard. Avoid valuables or items that include your personal information.

COPYRIGHT 2016 BY JANET NELSON, MSW, LCSW, LLC – ALL RIGHTS RESERVED – MORE INFO AT www.everydayselfdefense.com

Personal Safety in Clinical Practice

Phil Quinn, Ph.D., Director SSMH, EAP Program

Ray Mason, Director SSMH, Metro-Suburban Outreach

(1) - Awareness on the job. - 1.6

We must remain continually aware of safety issues in our daily clinical interactions

Some Statistics

Risk factors: mental health profession. (on the job.

- 2006 study of 5000 workers surveyed indicated that 55% said they faced safety issues on the job.
- 2002 survey of 800 workers 19% had been victims of violence and 63% had been threatened.
- Another study-50% of psychiatrists reported an act or threat of violence in past year.
- Mental Health profession- 2nd highest rate for risk of violence.

Areas of Personal Safety

- In your clinic office.
- In your outreach to the community (i.e. home-based).
- In your interactions with your clients.
- In your documentation (legal).
- In transportation.
- In your own home.

Observe and Plan

- Set up of your office.
- Do a safety assessment.
- Pay attention to signs of danger.
- Don't become complacent.

awareness, planning.

Knowledge

- Review any documentation
  1. Is there any history of violence?
  2. Have there been issues with previous therapists?
  3. Is drug or alcohol abuse an issue?
  4. Have there been psychiatric hospitalizations?
  5. Is client medicated?

1 1.1 docs

Initial Evaluation

- Past violence – is best predictor
- Drug and alcohol use and abuse
- Explore history with other helpers
- Access to weapons
- Psychiatric hospitalizations and history

face-to-face

Observe Client

- Do they appear clear?
- Are they pacing or over anxious?
- Are they mumbling or swearing?
- Any other signs of agitation or confusion?

Client Appears Impaired

1. Alert a colleague of your concerns
2. Ask if anyone is available to sit in, at least until you assess the situation.
3. Leave door open during session, if there are others around who could come to your aid.

Use Your Judgment

- If you see a potentially dangerous situation and you can't feel safe use your judgment and cancel.

Home Based Outreach

- Learn about the area before you go.
- Go with a team member.
- Make first appointments for early in day. Day-light hours are more safe until you know the area and the client.
- Be specific about appointment time.
- Make sure car has gas and is in good working order.

Outreach Cont:

- (4.) Park car in lighted area or accessible area.
- Dress appropriately-no jewelry.
- Make sure cell phone is working and pre-programmed.
- Call to alert family you are on the way.
- Carry a flash-light.

(1) 2 (3) (3.1)

Outreach

- Be observant of building or house.
- Listen before you enter house or apartment.
- Introduce yourself clearly.

(1) 2 (3) 3.1.

When to be concerned:

- Street lights are out or area appears too dark.
- Can't park close enough to feel comfortable.
- Gathering of strangers or teens that cause concern.
- Your instinct tells you it may not be safe.
- ALWAYS PAY ATTENTION

During the visit:

- Remain constantly aware.
- If person or persons you made visit with are not available, LEAVE
- Be clear about who you are and purpose. Don't allow for confusion.
- Too much activity, other people, party LEAVE
- Unsafe items, weapons, drugs-LEAVE
- Control where meeting takes place.

Transportation

- Keep keys with you and lock doors when no one is in car.
- Prior-Greet client assess risk.
- Seating-behind passenger seat.
- Drive in right hand lane.
- Use traditional 10 and 2 position.

Transportation

- Continually remain aware of mental status of client.
- Discuss concerns with supervisor prior to trip.
- If client is agitated or aggressive-do not transport-use police or ambulance.

Legal

- Keep records up to date
- Make sure they are legible
- Record everything (particularly unusual)
- Consult with others about difficult situations-record that you consulted

Ongoing, office or home

- Always be respectful.
- Set clear limits, on what you will not tolerate (intoxication, violence, 51A)
- Explain consequences
- Don't get lax in your vigilance
- Be observant
- Don't violate or bend your own rules

When Escalation Occurs

- Remember violence is often associated with fear or hurt.
- Stay calm and self-assured.
- Speak in a quiet, slow manner.
- Don't personalize the situation.
- Encourage client to be seated. If they stand you stand also.

0 1.5 1.6

Defusing Skills

- Listen, reflect their anger for them
- Remain non-defensive and supportive
- Acknowledge the anger
- Apologize
- Agree with the truth
- Invite criticism
- Allow extra space

0 1.6

What Action Steps

- Be firm – but not challenging
- Use your clinical skills
- Get to yes
- State your desire to help
- Be clear and direct

1.
1.5

Action steps:

- Offer choices
- Distract – offer candy, gum, coke
- Suggest time out or break
- Leave or call for help

1.
1.5

Things Not Advised

- Don't challenge or demand "calm down"
- Don't touch
- Don't give ultimatum
- Don't block exit
- Don't get into staring contest
- Don't show fear
- Do not turn your back on the person

If Attacked

- Yell for help.
- Defend yourself.
- Use object- phone lamp etc.
- Grab onto client-hang on them.
- Use knee to groin- twist fingers etc.
- Use any means to get free and get help.

Tips

Some Common Sense

- Don't see client in isolation.
- Pay attention to your own feelings.
- Use supervision.
- End session if you have real concerns.

(1) (1.6)

A Final Note

- Remember most of our work is safe. A majority of the clients and families we provide treatment to are not a threat to our safety.
- Safety assessments and action plans are important simply because they promote awareness and reduce fear to allow us to better serve our clients.

Thank You

- Questions / Comments

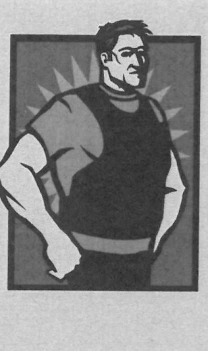

WORKER SAFETY

By
Orlando Cuadrado, MSW
and
Michael Smith

Goal

Develop skills in working effectively and safely in stressful and potentially dangerous situations

Definition of situations that impact safety of social workers.

Objectives:

- Identify potential safety issues
- Recognize three levels of awareness necessary when working in potentially dangerous situations
- Relate the four phases of crisis
- Develop skills to utilize in crisis situations

Objectives:

- Relate some techniques utilized to avoid dog bites ① 1.5
- Describe the process of reporting a safety incident ⑤ 5.1

Do you see what I See?

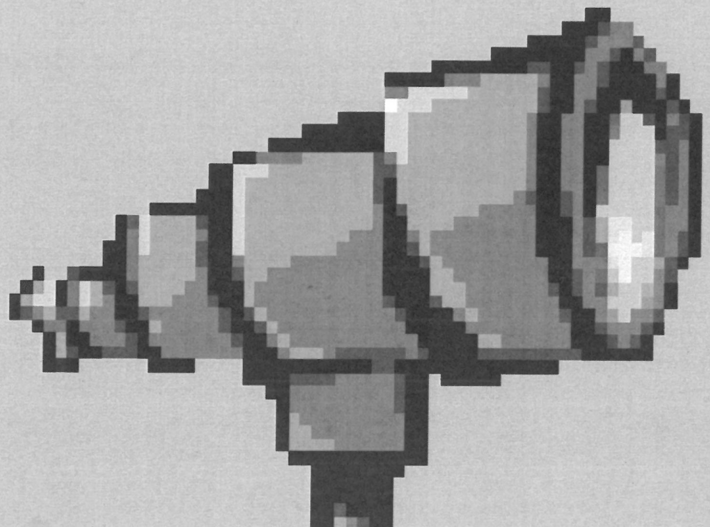

Reality Check

Workers that practice good casework skills can diffuse most crises and deliver services without jeopardizing the worker or the client's safety

(1) (1.5)

Values

- of a SW?

not sure where this belongs

- Strength Based
- Dignity
- Respect

Crisis Intervention Theory:

Dealing with potentially dangerous people ①

(1.5)

- What is crisis?
- Behaviorally- a change in the order, equilibrium, or the status quo.
- Dynamically- we need to understand that some clients have never learned or developed effective, rational, or constructive coping skills.

* This means a crisis is more likely to escalate

3 Levels of Awareness

skill / coping mechanism / prevention technique.

①

all awarenesses adressed here can be interpreted as [prevention & management of client violence] ①

specifically under the psychological component.

(1.6) 1. Self-Awareness

- What am I feeling?
- Be selective about personal disclosures
- What are their overall needs, and;
- What is the best response I can give?

3 Levels of Awareness

- Should you wear jewelry?
- Should you wear high heels?
- Should you carry a purse?
- Should I "buddy up"?

3 Levels of Awareness

1.6

- **Transference** – implies a psychological component to dealing with clients. \ SW should be aware of this

  The reproduction of positive or negative feelings that were at one time associated with another person or a past experience that are now (unconsciously) transferred to a new individual (typically someone who is in charge or an authority figure).

3 Levels of Awareness

- Counter-Transference - psychological component.

(1.6)

The same dynamic only in reverse, it occurs in the authority figure towards the client. client <=> SW

3 Levels of Awareness

2. Environmental Awareness (1)

Before you leave the office you should fill out a field safety form:

(1.2.) administrative way to cope w. CV + ensure safety

- Name and address of clients you are visiting
- The specific directions you are taking
- Rural setting
- Estimated time of arrival and departure
- The nature of your visit
- Your cell number

3 Levels of Awareness

- Is it a high crime area?
- If there are any safety concerns with the neighborhood plan early a.m. appointments.

3 Levels of Awareness

* Transportation could also be a code as it relates to transporting clients, but also making sure your own transportation is adequate

transportation (4)

When selecting a car to use in the field...

- Is it in good driving condition? (4.3)
- Does it have enough gas?
- The license plate and make, model, color of the vehicle you are driving
- Place any valuables out of sight.
- Park in well lit visible areas. Travelling to site. + (3.3)
- Don't get out of the car if you don't feel safe.

3 Levels of Awareness

3. Client Awareness

– Background about the client.

(1.1)

Is this case high risk?

- History of violence (DV,etc.)
- Substance abuse history
- Guns in the home
- Past criminal history
- Mental illness
- Possible removal

3 Levels of Awareness

In the field.....

(3.3)

- Establish the goal of the visit
- Keep a clear path to the exit door
- Avoid the clients bedroom unless absolutely necessary
- Always respect their personal space

3 Levels of Awareness

unclear_ data

Four different levels of space:

1. Intimate space- 0 to 1 arm
2. Personal space- 1 to 2 arm
3. Social space- 4 to 12 feet
4. Public space- 12 feet and more

3 Levels of Awareness

out of context. needs verb. clarification ?

- Lice
- Communicable diseases/illnesses
- Dogs
- Sexual threat and innuendo

Canine Considerations

* not pertaining to threat by other humans but the threat of an animal...

Canine Considerations

3.3

- Dogs bite!
- Getting Bit Hurts!
- Any dog can bite!
- Look for signs of a dog before entering the yard
- Don't surprise a dog…. whistle, tap, call out

Canine Approach Techniques

- Take off Sunglasses
- Stand Still – Let the Dog Approach
- Stand Sideways
- Speak Softly
- Don't Stare at the Dogs Eyes
- Observe the Dogs "Body Language"

3.3 ①

Canine Protection Techniques

- Don't Turn Your Back
- Use Firm Commands
- Give the Dog Something Other Than Your Hand to Bite
- Don't Run.......

(unless you think you can win the race!)

3.3 ①

Defense Against Dog Attack

- Maintain Self-Defense Stance
- Yell
- Shield Your Neck & Face
- Feed the Dog Your Notebook
- Vulnerable Parts of a Dog…..
  (nose, throat, chest & ribs)

① 3.3

THE FOUR PHASES OF CRISIS

↳ equips sw with knowledge about how a crisis can unfold.

↳ draws on ideas of consciousness / awareness.

(1.G)

Anxiety Phase

- Autonomic Body Responses
- Speech
- Motor Activity

(1) (1.G)

Anxiety Phase

- Worker Skills During This Phase
  - Culture
  - Empathy
  - Building rapport
  - Active listening
  - Be Supportive

Verbal/Non-verbal Communication

- Tone
- Volume
- Cadence

1.6. 2.3. +

CRISIS INTERVENTION TECHNIQUES

- Identify yourself and agency
- Explain your reason for being there
- Explain your responsibility to investigate
- Inform the parents of their rights
- Let the clients know you understand their feelings

1 3.3. 2.3

Defensive Phase

3.3. 2.3

They are beginning to lose control.

- Focus on feelings and behavior.
- Process Messages differently
- Your affective delivery is more important than the content
- They begin "pushing buttons"
- They challenge your authority

① how to spot someone who is triggered

Defensive Phase

3.3. 2.3.

Investigator takes control of the potentially escalating situation by setting limits

?

- S.O.D.A.S ? Unclear — data
- Proxemics
- Kinesics

①

Defensive Phase

1.1.

Personal Space Boundary Factors

- Culture
- Size
- Relationship
- Gender
- History

↳ requires knowledge / profile about the client / situation.

CRISIS INTERVENTION TECHNIQUES

1 3.3. 2.3.

- Project a calm assured feeling that you will see the situation through to a peaceful end no matter what happens
- Don't continue to push the client for specific answers, if the questions tend to increase anger
- Don't corner the client physically or psychologically

CRISIS INTERVENTION TECHNIQUES

DON'T.....

- Appear afraid or unsure of yourself
- Appear bossy, arrogant, nor assume an "I don't give a damn" attitude
- Don't become defensive
- Don't take clients anger as a personal attack

(1) 3.3. 2.3.

Aggression Phase

The stage in which the person will discharge their tension.

(1) Worker Skills During This Phase

- Observation
  - Is there a dramatic change in behavior?
  - Hyperactivity?

3.3. 2.3.

Aggression Phase

common sense?

- Shortness of breath
- Posture, clenching of jaw or fists
- Fixed stare

Aggression Phase

- Terminating the interview
  - Get away ASAP
  - Take actions to ensure your safety (Discuss policy)
  - Terminate in a way that will not embarrass the client

① 2·3 + 3·3.

Tension Reduction Phase

Characterized by a noticeable decrease in the tension level.

Intervention Strategies:

- Process the incident

Video Exercise

When should I bring the Five-O?

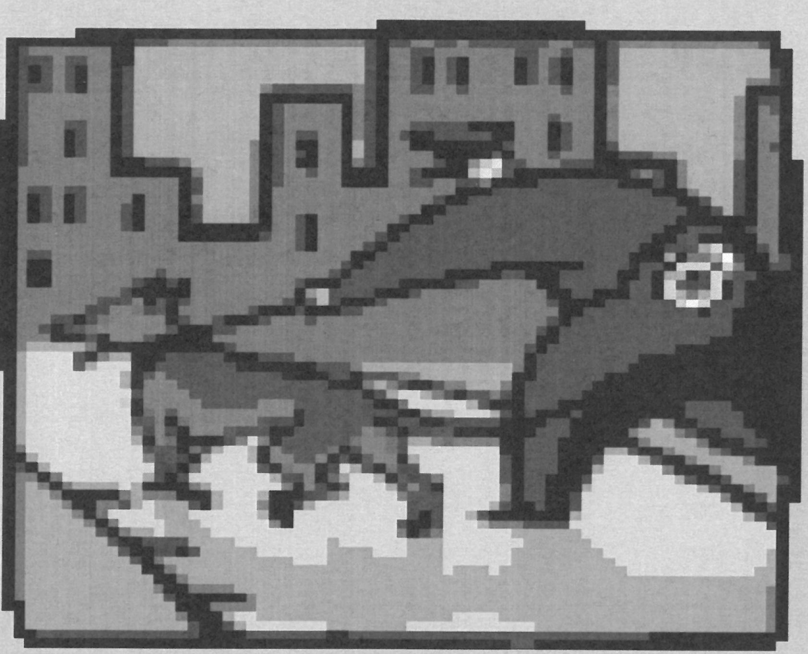

THE END

Lawmakers introduce bipartisan bill to improve social worker safety

Nov 18, 2019

WASHINGTON, D.C. – The National Association of Social Workers applauds a bipartisan group of House and Senate lawmakers for introducing The Protecting Social Workers and Health Professionals from Workplace Violence Act of 2019 (H.R. 5138 /S.2880), legislation that is a crucial step forward in curbing an epidemic of violence against social workers and other helping professions. The original sponsors of the bill include Sen. Kyrsten Sinema (D-AZ), who is a social worker, Sen. Lisa Murkowski (R-AK), Rep. Julia Brownley (D-CA) and Elise Stefanik (R-NY).

The legislation will provide grants to states to help employers improve facility safety, provide safety training to staff and purchase safety equipment. It also allows for support services, counseling and additional resources for staff who have experienced violence in the workplace.

"As a social worker, I've seen firsthand the critical roles that nurses, social workers, and other caseworkers serve in Arizona's communities. Strengthening workplace safety shows our appreciation for social workers and health professionals and will help recruit and retain qualified specialists to serve vulnerable Arizona families," Sen. Sinema said.

"Unfortunately, violence in the workplace is on the rise in America, with social workers and healthcare professionals facing disproportionate and alarming levels. These men and women have dedicated their lives to helping others, sometimes in dangerous and high-risk situations. We must ensure they have access to the necessary tools, training, and technology to keep themselves and others safe, as well as appropriate services to support employees that experience a violent or traumatic event," said Sen. Murkowski. "Recognizing the need to address the safety and risk factors associated with these careers, I'm proud to help lead bipartisan legislation that will help states, as well as Tribes, provide critical safety measures to social workers, health workers, and human services professionals."

Social workers are on the front lines in providing mental health and other services in a host of settings, including in people's homes. However, they are often victims of violence when they are on the job – and the problem is getting worse.

More than 75 percent of social workers report that they have experienced violence at work. In the health care and social assistance sectors, 13 percent of days away from work were the result of violence in 2013, and this rate has increased in recent years, according to the Bureau of Labor Statistics.

Examples of workplace violence include direct physical assaults with or without weapons, written or verbal threats, physical or verbal harassment, and homicide.

"Violence at work not only hurts social workers but hurts the clients they serve. Safe environments are healing environments" stated Dr. McClain. "We appreciate the leadership of our congressional champions in promoting workplace safety through this much-needed legislation."

The National Association of Social Workers (NASW), in Washington, DC, is the largest membership organization of professional social workers. It promotes, develops, and protects the practice of social work and social workers. NASW also seeks to enhance the well-being of individuals, families, and communities through its advocacy.

NASW CEO Angelo McClain urges Congress pass "Workplace Violence Prevention for Health Care and Social Service Workers Act"

Feb 26, 2019

Thank you, Chairwoman Adams and Ranking Member Byrne, and Members of the Subcommittee, for the opportunity to testify regarding the workplace violence challenges faced by social workers, health, and social service workers and the need for prevention and protection. I am also pleased to support and address the importance of the "Workplace Violence Prevention for Health Care and Social Service Workers Act" (H.R. 1309). I am Dr. Angelo McClain, Chief Executive Officer of the National Association of Social Workers (NASW). Our organization, which was founded in 1955, is the largest association of professional social workers in the nation with over 115,000 members and 55 chapters. Part of NASW's mission is to promote, develop, and protect the practice of social work and social workers. There are over 600,000 social workers in the United States, and they are the nation's largest provider of mental health services.

The National Association of Social Workers is proud to support the Workplace Violence Prevention for Health Care and Social Service Workers Act. This bill is a crucial step in reducing the staggering number of preventable physical and psychological assaults on social workers and other health and social services professionals. Developing a standard that anticipates the risks associated with the practice of social work is critical to preventing violence in those settings.

As the committee seeks to comprehensively address this important safety issue, NASW also urges you to consider the soon-to-be reintroduced "Social Worker Safety Act of 2019". This legislation would establish a Social Worker Safety Grant Program within the Department of Health and Human Services to aid state efforts to improve workplace safety measures for social workers. This bill provides states with critical resources designed to alleviate workplace violence threats by allowing grant money to be used to purchase safety equipment, make facility improvements, facilitate safety training programs, provide support services for social workers who have been victims of violence, or track incident data to mitigate future offenses against social workers, among other important uses. This bill was first introduced in 2007 as the "Teri Zenner Social Work Safety Act" and was named after a social worker in Kansas who was tragically stabbed to death by a 17-year old client in 2004. NASW also urges you to consider another soon-to-be reintroduced bill, the "Dorothy I. Height and Whitney M. Young, Jr. Social Work Reinvestment Act". This legislation would establish a Social Work Reinvestment Commission to provide independent counsel to Congress and the Secretary of Health and Human Services on policy issues related to recruitment, retention, research and reinvestment in the profession of social work, and for other purposes. A key focus of the Commission's efforts would be to improve social worker safety, and the bill also authorizes grants to assist entities in carrying out a workplace improvement program.

I urge Congress to take up and pass the "Social Worker Safety Act", the "Dorothy I. Height and Whitney M. Young, Jr. Social Work Reinvestment Act" and H.R. 1309, as these complementary measures will lead to safer workplaces for those performing important services that put them in high-risk and potentially dangerous situations. Trauma and violence must no longer be accepted as part of the workplace for health care and social service workers.

On a daily basis, social workers in a variety of settings are in harm's way. Our profession works in home care agencies, hospitals, child guidance centers, family services agencies, schools, mental health clinics and case management agencies, to name just a few settings. These are jobs that often require work beyond the agency walls where the risk of threats and violence are more prevalent. However, even within agencies we have had reports of incidents of violence against social workers. For this and other reasons, social work is among the top 10 most dangerous professions. Social workers and health professionals are twice as likely to face job-related violence as compared to other occupations. Between 2011-2013, there were 23,000 workplace assaults, and nearly 75% of these were in healthcare and social service settings (OSHA, 2016). In 2013, 1,100 social workers were injured as a result of workplace violence, according to the Bureau of Labor Statistics

(BLS). Unfortunately, these staggering statistics do not capture the substantial number of unreported assaults, which, according to one survey, are as high as 85% of all assaults (AFGE, 2016). In a 2003 survey of 1,600 social workers, 58% of the 1,129 respondents said that they had experienced at least one violent incident in their career (Newhill, 2003). In 2004, NASW partnered with the Center for Health Workforce Studies, University of Albany, to conduct a national safety study of 10,000 licensed social workers. 44% of the respondents reported facing personal safety issues in their primary employment setting and 30% felt that their employers did not adequately address safety issues. Many social workers are employed by public agencies and are placed at increased risk due to the settings in which they work and the nature of the services they perform (NASW, 2004). Additionally, preventing workplace violence is a key success factor in reducing clinician and staff burnout and increasing retention.

I would like to highlight the unique and significant risk child welfare workers face. Violence against these workers is prevalent for several reasons. Child welfare clients are not receiving services voluntarily, and their families often have other volatile issues, such as domestic violence and substance abuse. Further, child welfare workers often make home visits in struggling neighborhoods. Child welfare workers spend a large percentage of their time in communities and, as a result, may experience a higher risk of harm. Additionally, these child welfare workers tend to prioritize physical and emotional safety of the child leaving child welfare workers more vulnerable to becoming targets of violence themselves. Finally, many state governments currently are suffering from budget cuts; therefore, adequate funds to properly train and protect public workers are not prioritized. The Social Worker Safety Act aims to provide states with these much-needed resources.

Increased rates of violence in society, deinstitutionalization, and greater enforcement around child custody have heighted the inherent risk to social worker safety. These professionals enter dangerous situations regularly, and, unlike police or probation officers, they often enter these situations without any form of self-protection. In their day-to-day work, they encounter volatile situations that can quickly escalate to physical violence. Their clients include individuals who have violent histories, may be psychiatrically unstable, or experiencing extreme emotional stress. Because of the nature of social work, these professionals are also often involved in high-conflict situations, such as child custody disputes, removal of children from parents, and work with probationers and court-involved individuals. Social workers serve vulnerable populations and those with serious, chronic physical and mental conditions. As a critical workforce serving underserved populations that have often been disconnected from health and mental health care, social workers are at times subject to unpredictable situations and environments.

I want to illustrate some of the tragedies social workers have experienced due to workplace violence. In Congressman Courtney's home district in Connecticut, a social worker was murdered by a client in 1998 as she was entering her agency. This social worker posthumously received the Connecticut NASW chapter Social Worker of the Year Award. In 2008, there were two fatalities. New York social worker Brenda Yeager was beaten and suffocated while visiting a client family home and Diruhi Mattian was killed in Massachusetts during a home visit with a client. In 2009, Retired Commander Charles Keith Springle, a Navy social worker, was shot and killed, along with four other troops, by another service member seeking counseling services at Camp Liberty in Baghdad. In 2011, Stephanie Moulton, a social worker in Massachusetts, was killed by a client with mental illness at a group home. In 2012, Stephanie Ross, a caseworker in Tampa, was stabbed to death by a client. In 2015, Lara Sobel, a social worker in Vermont, was killed in her workplace parking lot by a client who had recently lost custody of a child. In 2018, Pamela Knight died while working to protect children in her capacity as an investigator for the Illinois Department of Children and Family Services. In addition to these tragic fatalities, there are a staggering number of assaults that are often under reported. Every day social workers across the nation provide a wide range of services in increasingly complex environments. Workplace violence against social workers is an occupational risk hazard that is preventable and needs to be addressed systemically at all levels of society.

As a licensed and practicing social worker over the past 30 plus years, and as former Commissioner of the Massachusetts Department of Children and Families from 2007 to 2013, I have been directly and indirectly involved in numerous workplace violence situations, including numerous assaults, both

physical and verbal, on social workers and other health professionals. I have never been physically assaulted, but I have been threatened with physical harm on a number of occasions with guns, knives and other weapons. I also have experienced verbal assaults. During the first hour of my first day on the job as a social worker, I went on a home visit to see a family regarding a potential abuse situation. Upon entering the home, the mother closed the front door and within a nanosecond got a butcher knife and held it up to my face. She threatened "If you get us in trouble, I will hunt you down in a dark alley and kill you". Due to a combination of my size, street smarts, social work training, and ability to defuse situations, I was able to move this confrontation to a constructive interaction and avoid any physical harm. Just a few years later, I went on a home visit with another family. The mother was sitting on the front stoop and we began discussing the possible removal of her children. Within minutes, I found myself surrounded by a crowd of neighbors, one of whom was carrying a gun. I told the group to disperse, and they did, but this could have had a tragic ending. I share these experiences to put a human face on the urgent need for action, and to underscore how very many of us in the social work profession encounter actual or threatened violence in the workplace.

The Massachusetts health and human services community has been repeatedly stunned by the deaths of their own (Stephanie Moulton in 2011 and Diruhi Mattian in 2008), as well as other harms to social workers in the state. In 2005, a 10-year veteran of the Massachusetts Department of Social Services retired due to a traumatizing experience of being stalked by a teenage client. Describing her decision to leave her job, she said "I doubt myself now. I always went into every home with an open mind. I don't know if I trust myself to be fair after this. My babies have to come first. I can't put them at risk." She was tormented at the thought of leaving the profession she once loved. "Most of these kids have been abandoned by adults," she says. "I never thought I would end my career walking away from them, too." In 2009, Massachusetts Governor Patrick issued Executive Order 511 to address health and safety protections for commonwealth employees because public workers are not covered by federal Occupational Health and Safety Administration (OSHA) standards and rules. That order established the Massachusetts Employee Safety and Health Advisory Committee, which was tasked with examining the safety of state workers and making recommendations to reduce workplace injuries and illnesses. In 2014, the Committee issued a report based on a 2010-2012 study showing that violent assaults, among others, caused the most injuries to state workers in Massachusetts. Approximately 3,000 Massachusetts state workers experienced job-related injuries serious enough to require time off from work, and four workers lost their lives during that time period. Notably, the most at-risk state employees were health and human services workers, corrections officers, and transportation workers.

While I was Commissioner in 2013, Governor Patrick signed the Social Work Safety in the Workforce bill, which requires all direct services providers receiving funding from the state's Executive Office of Health and Human Services to provide workplace violence prevention and crisis response plans. This legislation, and the resulting regulations, which took effect in 2015, have been critically important in improving the safety of social workers and reducing staff burnout and improving employee retention in Massachusetts.

Recognizing the urgent need to address safety and risk factors associated with social work practice, NASW has long supported the development of policies and procedures designed to eliminate violence in the various workplace settings in which social workers practice. NASW also supports the conduct of research to document the extent of the problem and develop effective systemic solutions. In addition to NASW's dedicated advocacy in support of the Social Worker Safety Act, the organization in 2012 developed "Guidelines for Social Worker Safety in the Workplace" which are a crucial resource to communities, private and public agencies, and local, state, and federal policymakers committed to creating a safer work environment for social workers and related professionals. A copy of these guidelines is attached. These standards are based on the safety policy that was developed in Massachusetts during my tenure as Commissioner and address both primary trauma (e.g. physical and/or verbal assault) and secondary trauma (e.g., post-traumatic stress disorder, etc.). NASW safety guidelines include many of the tactics outlined in H.R. 1309, such as the use of safety technology (e.g. mobile panic buttons, security cameras), "buddy" systems for off-site client visits, comprehensive risk assessments of both

clients and work settings, incident reporting and logging practices, and annual training.

NASW has a variety of other resources available to employers and others aimed at recognizing the risks social workers face, identifying high-hazard work environments, and protecting social workers from these risks. This includes a publication, Security Risk, which outlines strategies and tactics around safety. Notably, Integra Health, which was involved in the case regarding the murder of Stephanie Ross in Florida, relied upon NASW's guidelines and resources in designing training for its service coordinators as part of its post-incident corrective actions.

Although policies such as those in Massachusetts and NASW's guidelines around safety have been mission-critical in protecting social workers, a strong OSHA standard is essential. It is essential that Congress, through H.R. 1309, impose a workplace violence prevention standard that is mandatory for covered workplaces and affects many public employees, a significant number of whom are social workers. NASW strongly supports all the recommendations for the OSHA guidelines. They are feasible and effective for protecting social service workers. NASW strongly supported OSHA's use of the General Duty Clause as a way to enforce safe working conditions absent a standard. But the General Duty Clause is burdensome, under attack (in Integra) and OSHA therefore needs a standard. The work practice and environmental controls required in each workplace violence prevention plan will save lives and help decrease the disproportionate number of incidents social workers experience. Congress should also enact the Social Worker Safety Act of 2019, which will provide needed resources so state employers can similarly work to provide safer workplaces for these vital providers. Finally, NASW urges Congress to take further action to address workplace violence by passing the Dorothy I. Height and Whitney M. Young, Jr. Social Work Reinvestment Act to provide additional resources and continued focus on this issue.

National Social Work Month is just a few days away. During March, NASW will, through our "Elevate Social Work" campaign, raise awareness about the incredibly important role of social workers in this nation. Congress must act now to pass H.R. 1309, the "Social Worker Safety Act", and the "Dorothy I. Height and Whitney M. Young, Jr. Social Work Reinvestment Act" to provide critically needed protection for the 600,000+ professionals who have committed their lives and careers to helping others, despite low pay, little recognition and, increasingly, dangerous working conditions.

Thank you again for the opportunity to testify, and I look forward to answering any questions you may have now or in the future.

The National Association of Social Workers (NASW), in Washington, DC, is the largest membership organization of professional social workers. It promotes, develops, and protects the practice of social work and social workers. NASW also seeks to enhance the well-being of individuals, families, and communities through its advocacy.

Safety Policy and Procedures

The Simmons School of Social Work Field Education Department has adopted the following policy and procedures regarding the safety of SSW student interns in the field. This policy has been created to recognize that violence in the lives of clients can create potential dangers to professional social workers and students engaged in the study of the profession. While social workers and interns may be more aware of these issues in urban areas, we believe issues of safety are relevant in all communities and settings. We urge SSW students to become familiar with this policy regardless of the location of your internship placement.

I. Policy

a) The School of Social Work is responsible for providing all students and faculty advisors with general written information about safety in field placement.

b) The SSW expects each agency to be responsible for orienting student interns to the safety policies and procedures of that agency and setting. Such orientation should include, but not be limited to, discussion of safety issues in the community, within the agency building(s), with particular clients prone to violent behavior, and about clients who may be sharing living quarters with persons prone to violent behavior, to the extent that such information is known. Security of personal belongings of the SSW intern while at the agency should be also covered. Procedures for the student(s) to follow in the event of a safety or security problem should be reviewed at the beginning of the placement with ample opportunity for questions and discussion as needed.

c) SSW Students should not be required to engage or to remain in assignments or at placements in which they feel physically at risk. SSW urges all agencies to make the same accommodations to ensure students' safety as they make for their own agency staff and in some situations the agency may need to make even greater accommodation for a student. If a student's concerns about safety interfere in whole or in part with the learning process, the faculty advisor should be contacted by the field instructor to facilitate prompt exploration of the concerns and to seek a mutually satisfying resolution.

II. Procedures

a) If an incident occurs in which a student is personally threatened or hurt it is the student's responsibility to notify the field instructor immediately. The field instructor, agency contact person, or agency director should then contact the Director of Field Education immediately to discuss what actions the agency and Simmons SSW should take to ensure the student's physical and emotional well-being in the wake of the incident and on a going-forward basis.

b) The Director of Field Education will document the incident and the steps taken to address it and will meet with the student and faculty advisor to assess the student's readiness to return to the field, and any other issues relevant to the situation. The Director of Field Education may alert the Dean or Associate Dean of the SSW about incidents and concerns on an as-needed basis.

III. Safety Guidelines for Students in the Field

a) Agency Protocol

It is important for students to know the Agency's safety and security protocol for office and home visits with clients prior to the start of the placement. If the agency does not have safety and security policies and/or procedures the field instructor and student should review and discuss any issues related to safety and security in the setting. SSW students are urged to bring their questions and concerns to the field instructor. Regular communication particularly about safety concerns is strongly encouraged.

The following are guidelines and suggestions that may be helpful to students, field instructors, and faculty advisors as they consider the particular safety issues in their settings. Specific steps taken by students or agency personnel will obviously have to be determined by the individual situation, the nature of the setting, etc.

b) Security of Belongings

All students in the field are expected to have a secure place to keep coats, handbags, cell phones, laptops, and other belongings while at placement. It is preferable that the space be one that can be locked, and could be in a desk drawer or filing cabinet. Students should not leave handbags and other personal articles visible and unattended, even in an office with the door closed.

Valuables should not be brought to placement settings. Items of value should not be left in cars, and should not be placed out of view just prior to leaving a vehicle. Neither the agency nor Simmons can be responsible for lost, stolen or damaged personal items.

c) Safety Issues Related to Working with Clients

When working with clients, it is important to remember that the treatment process often makes people feel vulnerable and may challenge their usual coping mechanisms. With some people, this can contribute to problems with impulse control, and can raise issues of safety for the client, the social worker, the student intern and others.

There may be times when students work with individuals who have difficulty with reality testing, dealing with overwhelming emotions, and controlling their anger. Some of them may be prone to violence and may possess a weapon. Other clients may be intoxicated, high on drugs, in withdrawal, or may have other medical or neurological disorders. Again, we urge all SSW students to consult with your agency field instructors to prepare adequately for handling of specific situations that are potentially difficult or threatening, such as medical emergencies, suicide or homicide risks, potential abuse of others, and the presence of weapons.

d) Safety Guidelines for Office Meetings

If a student will be meeting with a client with whom the student does not feel safe, it is important for the student to discuss the situation promptly and fully with the agency field instructor. Based on the outcome of this discussion, there may be a decision that a student will not see the client or see the client under specific circumstances or controls. However, if it is decided that a student will see the client, several points should be considered. A client's mental status should be assessed. When considering location of the meeting, it might be helpful to think about what is in the room, whether there is more than one exit, and where each person might sit. It may also be helpful to think about whether to include someone else in the meeting, and what to wear. When discussing the time of the appointment, it can be helpful to think about whether or not many people are around at the time being considered for the meeting. It is also important to discuss the plan for backup and assistance in the event that the client becomes agitated. A student should never see a potentially dangerous client alone without someone else in the agency knowing about the client, the appointment time and the location of the appointment. Students should keep supervisors informed about their schedules at the agencies at all times.

e) Safety Guidelines for Travel by Car

When a student is traveling by car to an agency or to home visits, it is advisable to know where he or she is going, and to look at a map before driving to unfamiliar areas. In general, remember to be alert, and to lock doors and close windows. The student should tell someone where he or she is going and the expected amount of time she/he will be away from the office. The agency should have your cell phone number or other information on how to contact you in the event of an emergency.

f) Safety Guidelines for Travel by Foot or Public Transportation

When traveling by foot or public transportation, it is advisable that students know where they are going and the route by which they will travel. Students are encouraged to carry the least amount of valuables with them as possible. Money, license, keys, and other essentials might be carried in a pocket. If a handbag carried under the arm is grabbed, it is best to let go of it. It is helpful to dress in comfortable clothes that are loose fitting, and to wear sturdy, flat walking shoes. It is also helpful to be alert, and to walk with a purpose, as if one has a clear destination. One should be aware of people in the immediate area.

g) Safety Guidelines for Home Visits

It is important to familiarize yourself with the clients' file prior to the home visit. If there is a question of safety, plan accordingly with field instructors. It might be decided that meeting at a neutral place or going with another worker is the appropriate plan. Someone at the agency should always know the student's itinerary It is helpful to stay alert and to think about what to wear, which room to meet in, and where to sit. If a student ever feels threatened at any point during the interview, they are encouraged to err on the side of caution and appropriately terminate the visit. If clients seem to feel threatened by the student entering their dwelling, the student should desist and not force the issue. If a student hears a heated argument from inside the house or apartment, the student may decide to re-schedule the visit or call to assess the situation before entering the dwelling. A student should never see a potentially dangerous client alone without someone else in the agency knowing about it. In general, a cell phone is very useful for students doing home visits.

h) Health Safety

Students should be alerted to the existence of biohazards. They should receive training and information about how to protect themselves from infectious diseases.

IV. Post Incident Protocol

If an incident occurs in which a student is personally threatened or hurt, the field instructor, agency contact person, or agency director should contact the

Director of Field Education immediately to discuss what actions the agency and School should take to ensure the student's physical and emotional well-being.

The Director of Field Education will document the incident and the steps taken to address it, and will meet with the student and faculty advisor. Together, they will assess the student's readiness to return to the field and any other issues relevant to the situation.

Simmons SSW thanks Boston University School of Social Work for sharing their safety policy which was used in developing the SSW safety policy.

APRIL 2012

leadership ladders:

STEPS TO A GREAT CAREER IN SOCIAL WORK

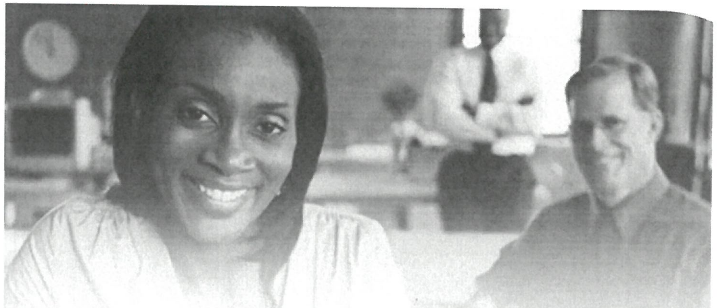

organizing for office safety

The door to safety swing[illegible]he hinges of common sense.

~Author Unknown

Safety is often on the minds of social workers. A significant portion of a social workers' job can require work in the community, in addition to working in office space. Social workers can be confronted with potential safety issues such as managing angry or mentally unstable clients and working alone during evening and nighttime hours. Job-related violence affects not only the professionals who experience it, but also their families, their clients and their communities (Kelly, 2010).

Social workers often receive training regarding managing safety issues while in the community, however, they should also be aware and prepared to ensure their safety as well as possible when they are in a familiar setting such as their office. Being prepared and using basic techniques can go a long way in providing for a safe work environment. Consider these approaches to increase office safety:

» **POSITION OFFICE FURNITURE STRATEGICALLY.** When meeting with clients in an office, strategic positioning of the office furniture can assist the social worker in the event of a dangerous situation. Creating a clear path for the social worker to exit the office is one such strategy. In many agencies, the social worker's desk is positioned so that the client is closest to the door. Altering this arrangement to position the social worker closest to the door will enable the social worker to leave the office more easily if the social worker feels unsafe. Another strategy is to position the social worker's chair facing the door. This will help social workers easily see who enters their office and can prevent "surprise visits" from people entering the space without their knowledge.

NASW CENTER FOR WORKFORCE STUDIES

NASW
National Association of Social Workers

National Association of Social Workers
750 First Street NE, Suite 700
Washington, DC 20002-4241

» **KEEP THE OFFICE DOOR OPEN.** It can be customary to close the door when meeting with a client to ensure privacy and confidentiality. However, keeping the door fully or partially open can nonverbally communicate to the client that others are nearby and are listening or watching. Consulting with a supervisor may be necessary in assessing the appropriateness of this approach especially with regard to confidentiality requirements. This can be a helpful preventive technique when working with a client who may be angry, verbally abusive or aggressive.

» **BE AWARE OF POSSIBLE "WEAPONS" IN THE ENVIRONMENT.** Ordinary objects such as a fire extinguisher, stapler, paper weight, and sharp items, such as a letter opener or pair of scissors, can be easily used as a weapon by someone in an angry state. Limiting the presence of these potential weapons or restricting access to them, such as placing them in a drawer, can enhance safety.

» **PARTNER WITH A SAFETY BUDDY.** A safety buddy is someone who collaborates to maintain safety, such as a co-worker or supervisor. A safety buddy can be used to look for signs of danger when a social worker is meeting with a client and call for help if necessary. The safety buddy should have a plan of what to do if a dangerous situation occurs and should have the needed resources readily available, such as a phone, phone numbers, escape route, etc.

» **BE THOUGHTFUL ABOUT AND COMMUNICATE CONCERNS AHEAD OF TIME.** Preparation for safety takes place before a situation occurs. Assess safety issues before each meeting as the first step in organizing for safety. Communicate concerns about safety issues and confirm that they are adequately addressed before the meeting. A record of this communication, prior to a potentially dangerous incident, can prove to be of great assistance during and after an emergency.

Be familiar with the agency's safety protocols and take an active role in helping the agency ensure safety for its workers.

» **TRUST YOURSELF.** There should be no need to take risks regarding personal safety in the workplace. Be familiar with the agency's safety protocols and take an active role in helping the agency ensure safety for its workers. Also, it is very important to trust your instincts in potentially dangerous situations. If a social worker feels unsafe, for any reason, it is important to solicit support, regardless of the results of a safety assessment or the client's history. With regard to safety, it is better to err on the side of caution, rather than face situations under-prepared. Feeling safe and secure when working with clients will ultimately benefit the client, as well as the social worker.

RESOURCES

National Association of Social Workers Center for Workforce Studies provides information on the social work workforce. This information includes helpful resources to enhance professional skills.
www.socialworkers.org

National Association of Social Workers' collaboration with the Center for Health Workforce Studies, University at Albany conducted a benchmark national study of 10,000 licensed social workers. The information presented in this fact sheet is based on this 2004 study and its findings.
http://workforce.socialworkers.org/whatsnew/safety.pdf

REFERENCES

Kelly, James J. (2010). The Urgency of Social Worker Safety. *NASW News* Volume 55, No. 9. Retrieved from www.socialworkers.org/pubs/news/2010/10/social-worker-safety.asp

©2012 National Association of Social Workers. All Rights Reserved.

Safety Tips for Home Visits From a Veteran NYC Social Worker

Pascale Victor, LMSW

Pascale Victor, author of Field Work with an Open Heart: Portraits that Unlock the Door to Your Clients' Secret Lives, is a licensed social worker with a degree from the Columbia University School of Social Work in New York City. Her extensive experience encompasses both direct clinical work and social work administration. She also has experience providing short-term therapy to adolescents, adults, and families, serving as a bridge between her clients and community-based organizations for continued long-term mental health treatment. She was formerly employed as a hospital social worker where she worked closely with psychiatrists in order to provide social service intervention to the youth who were brought to the pediatric emergency room as a result of mental health or emotional/behavioral problems. From 2002 to the present Pascale Victor has been employed as a social worker for the New York City Housing Authority (NYCHA). There she provides social service intervention to the residents of NYCHA: youth, adults, elderly, as well as families. Responding to emergency situations and providing intervention in crises are part of her responsibilities.

Ms Victor has compiled a list of safety tips for all social workers whose jobs, like hers, include field work. She believes some of them will also be helpful to other social work professionals who do not do field work.

Doing Home Visits? Err on the Side of Caution and follow these Safety Tips

1) For an initial home visit, try to schedule the appointment by telephone or letter so that the client will know to expect you and be prepared. If you speak to the client ahead of time, you may be able to get vital background information or an update on their current situation, which may have changed.

2) Whenever possible, conduct home visits accompanied by colleagues or employees from other agencies who are also working on the same case. If you are a woman about to conduct a home visit that is potentially unsafe, you may request that a male colleague accompany you. For example, I have a male co-worker who is 6'4" and wears sunglasses and an earpiece, so he looks like a secret service agent. He can definitely be intimidating, which is why I request his "bodyguard services" for some cases.

3) Depending on the nature of the case, some clients can come to an office, rather than have you meet them in their homes.

4) Always carry a charged cellular telephone.

5) Request a joint home visit with a police officer if you think the situation could become extremely dangerous.

6) Be sure to inform your supervisor and another colleague of your whereabouts.

7) Know where the exits are in a home and in building hallways.

8) Do not enter an elevator with people who are suspicious-looking or make you feel uncomfortable in any way. If you are feeling nervous, pretend that you are using your cellular telephone and cannot get on the elevator. When riding an elevator with someone who frightens you, immediately press the button of the next floor so that you can get off.

9) If you feel unsafe during an interview and believe you are or might be in danger, you should immediately end the interview and leave—run if necessary!

10) Depending on the case and any confidentiality issues, you can possibly get a client's trusted family member involved and conduct a joint home visit with that person.

11) Always be vigilant and assess the surroundings—both inside and outside a client's home.

12) Never stand too close to an apartment door. Clients often open their doors and allow their dogs to run out and jump on you. Request that the client put the dog or other pet in another room. It is also possible that a client could try and harm you, so stay back. It is rare but it is always better to be safe than sorry.

13) Always remember to keep your cool. Never show a client that you are scared. Always remain professional and if the situation gets out of control or dangerous—leave. Remember that you are the professional and are there to help the client. If you show that you are scared, the client might try to take advantage of the situation by being manipulative.

3.3.

14) Do not allow clients to play on your sympathy and good nature to get what they want. Stay firm and do your job to the best of your ability. Never allow a client to sway you in any way that is not for the good of the case. If you make a decision against your better judgment and only follow the client's wishes, the client may become very upset and refuse to be cooperative if you make contrary decisions later. A client may even "turn against you" and become belligerent and hostile.

1.6.

15) Do not get too comfortable and let your guard down with clients. Remember that you are providing a service for them—they are not your friends.

16) Depending on the case, it may be possible to meet in a public place such as a nearby park, community center, senior center, coffee shop, etc.

- Field visits
↳ not a home visit nor an office visit.

ensuring safety ~~to~~ during home visits.

17) Educate your clients about how to get rid of bedbugs with a professional pest service. Bedbugs often hide in living room furniture and bedrooms. Field workers who deal with bedbugs are most definitely at risk.

18) Wash your hands regularly. If you are out all day and are constantly touching door knobs, shaking hands and utilizing public transportation, it is easy to catch germs and spread them. Keep a hand sanitizer or wipes in your coat or bag.

19) If you are highly allergic to certain domestic animals then you should take that into account before conducting home visits. Many clients live with cats, dogs and other pets. If being around a particular animal triggers an allergic reaction, necessary precautions need to be taken into account ahead of time. In some cases you may need to see an allergist for guidance.

3.1

20) Always wear comfortable clothes and shoes while working in the field since you will be regularly walking, standing and climbing stairs.

- practical tips.

For more information, or to contact Ms. Victor, please visit www.pascalevictor.com

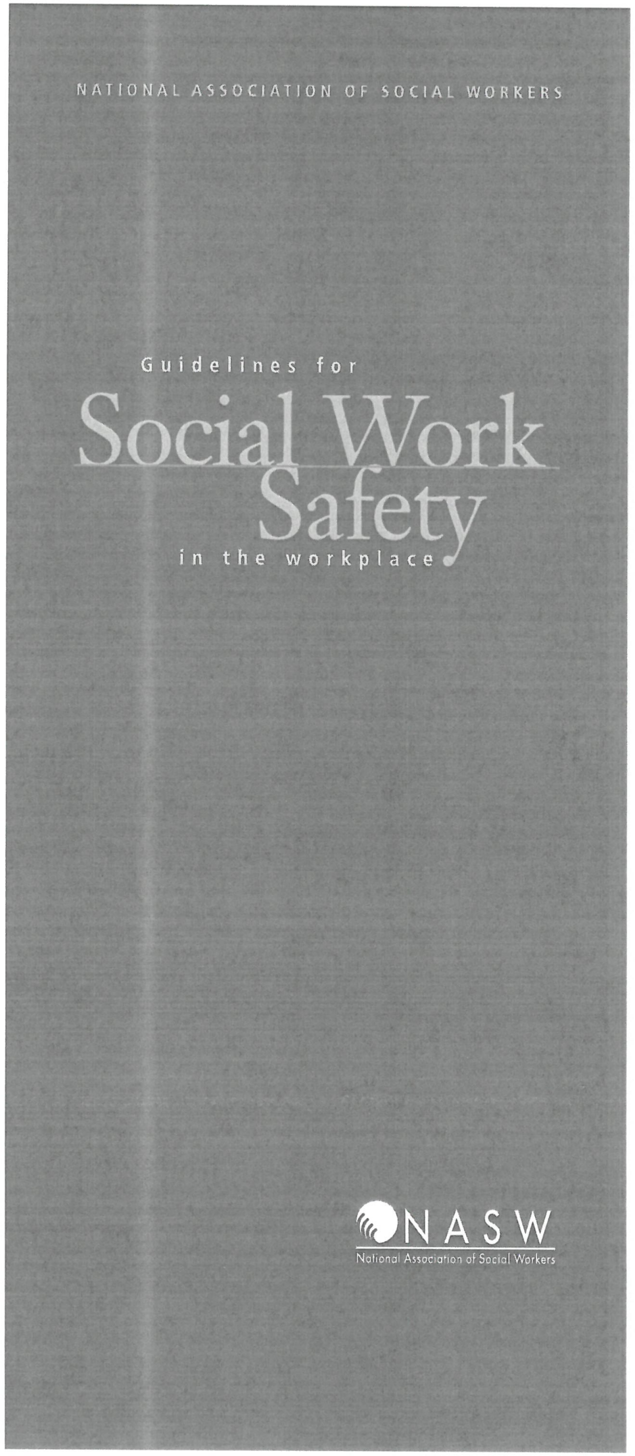
NATIONAL ASSOCIATION OF SOCIAL WORKERS

Guidelines for

Social Work Safety

in the workplace

NASW
National Association of Social Workers

- A core document that informed your codes?

About the Association

The National Association of Social Workers (NASW) is the largest membership organization of professional social workers in the United States with over 140,000 members. The mission of NASW is to enhance the professional growth and development of its members, to create and maintain professional standards, and to advance sound social policies.

Guidelines for Social Work Safety in the workplace

National Association of Social Workers
Jeane W. Anastas, PhD, LMSW
President

Elizabeth J. Clark, PhD, ACSW, MPH
Chief Executive Officer

Social Worker Safety in the Workplace Expert Panel
Beryl Domingo
Samuel A. Hickman, ACSW, LCSW
Shari Munch, PhD, LCSW
Christina E. Newhill, PhD, LCSW
Bekki Ow-Århus, LICSW, ACSW, DCSW
Eva Skolnik-Acker, LICSW
Earl "Skip" Stuck
Charles Dee Wilson, MSW

NASW Staff
Tracy R. Whitaker, DSW, ACSW
Kamilah Omari, LMSW-C&M, ACSW

©2013 National Association of Social Workers.
All Rights Reserved.

Contents

5 Introduction
6 Guiding Principles
6 Acknowledgment of the Context of Social Work Practice
7 Social Workers' Rights to Report Safety Concerns
7 Application of Universal Safety Precautions
8 Goals of the Guidelines
9 Guidelines for Social Worker Safety in the Workplace
9 Standard 1. Organizational Culture of Safety and Security
11 Standard 2. Prevention
12 Standard 3. Office Safety
13 Standard 4. Use of Safety Technology
15 Standard 5. Use of Mobile Phones
16 Standard 6. Risk Assessment for Field Visits
19 Standard 7. Transporting Clients
20 Standard 8. Comprehensive Reporting Practices
21 Standard 9. Post-Incident Reporting and Response
22 Standard 10. Safety Training
23 Standard 11. Student Safety
24 References

Acknowledgments

The development of these guidelines was inspired and informed by the work of the NASW Massachusetts Chapter Safety Task Force. We also gratefully acknowledge the work of NHS Security Management Service in England.

Introduction

A major tenet of the National Association of Social Workers' (NASW) threefold mission is to promote, develop, and protect the practice of social work and social workers. In alignment with that mission, NASW establishes professional standards and guidelines to support quality social work practice.

Social workers provide services in an increasingly complex, dynamic social environment and have a broadening client base. The profession's primary mission is "to enhance human well-being and help meet the basic human needs of all people, with particular attention to the needs and empowerment of people who are vulnerable, oppressed and living in poverty" (NASW, 2008, p. 1). Within the past decade, the United States experienced a severe recession and related social changes that have led to increases in the number of people unable to meet their basic needs without assistance from public and private agencies. These social and economic changes have created pressures in social work practice settings as more people seek social work services from agencies that have often experienced budget cuts and sometimes lack adequate resources to meet the needs of people desperate for help.

DEF of SW

Based on diverse environments, SW need safety protocols.

- vulnerable

pressure from larger structures

Unfortunately, the number and variety of people to whom social workers provide services and the variety of settings in which these services are provided have contributed to an increasingly unpredictable, and often unsafe, environment for social work practice. Social workers have been the targets of verbal and physical assaults

a large component

- settings. present danger to SW.

in agencies as well as during field visits with clients. Tragically, some social workers have also been permanently injured or have lost their lives "in the line of duty."

- morbidity.

Establishing safety guidelines for the profession is timely as the profession is expected to grow by 25 percent before 2020 (U.S. Department of Labor, Bureau of Labor Statistics, 2012). These guidelines are important to the retention and recruitment of a professional social work force. Moreover, NASW guidelines may be a helpful resource to communities; private and public agencies; and local, state, and federal policymakers invested in creating a safer work environment for social workers.

(1) Guiding Principles

The development of these guidelines is informed by the following principles:

Acknowledgment of the Context of Social Work Practice

These guidelines address safety and risk factors associated with social work practice, but they should not be interpreted to infer that social work is an inherently or unusually dangerous profession. Social workers acknowledge and understand that interaction with clients is a cornerstone of many practice settings. Most clients and families that social workers serve do not present threats or pose danger. In cases where threats are present, the majority of social workers find that their employers address these issues appropriately (Whitaker, Weismiller, & Clark, 2006). There are, however, social work

settings (for example, child welfare, adult protective services, mental health, criminal justice, domestic violence shelters) where social workers may face increased risks of violence. These guidelines are meant to support social workers in practice but are not meant to stereotype or denigrate client populations who receive services from social workers.

Social Workers' Rights to Report Safety Concerns

Social workers have the right to work in safe environments and to advocate for safe working conditions. Social workers who report concerns regarding their personal safety, or who request assistance in assuring their safety, should not fear retaliation, blame, or questioning of their competency from their supervisors or colleagues. — (5.1)

Application of Universal Safety Precautions

Social workers should routinely practice universal safety precautions in their work. Violence can and does occur in every economic, social, gender, and racial group. To avoid stereotyping particular groups of people and to promote safety, social workers should practice safety assessment and risk reduction with all clients and in all settings. A thorough understanding of the risk factors (individual/clinical, environmental, and historical) associated with elevated risk for violence can inform safety assessments. Social workers should also be aware of the potential that their personal information on the Internet, particularly social networking sites, can be accessed by anyone. Universal safety precautions also include the establishment of safety plans as a matter of routine planning. The adoption of universal safety precautions

profile-

applies to home + office visits. (2.4) + (3.1)

should not preclude agencies from establishing particular safety precautions when social workers are asked to perform dangerous tasks. In those situations, agencies should establish specific policies (for example, law enforcement accompanies social workers when involuntarily removing a child from parents or an incapacitated adult from a home) to reduce the risk of harm to social workers.

Goals of the Guidelines

These guidelines address safety within the context of social work practice. Ideally, these guidelines can stimulate the development of agency policies and practices to enhance social worker safety. In addition, social workers can use these guidelines to assess agency culture of safety and to advance professional and personal well-being. As well, schools of social work can better assure that their curriculums and field placement practices are in line with the goals of a safer profession. The specific goals of the guidelines are

- to inform social workers, policymakers, employers, and the public about the importance of social worker safety in agency and field work
- to provide a basis for the development of social work undergraduate, graduate, and field placement practice; and agency in-service programs, continuing education materials, and tools related to social work safety
- to advocate for social workers' rights to work environments that promote safety
- to support the exploration of technology that enhances social worker safety

- to encourage social workers to participate in the development and refinement of public policy that addresses social worker safety through licensing, regulation, and resources
- to encourage social workers to participate in the development, refinement, and integration of best practices in promoting social worker safety.

Guidelines for Social Worker Safety in the Workplace

Standard 1. Organizational Culture of Safety and Security

Agencies that employ social workers should establish and maintain an organizational culture that promotes safety and security for their staff.

Interpretation

Social workers should be able to practice in environments free from physical, verbal, and psychological violence and threats of violence. Workplace safety demands diligence from organizational leadership at every step of the safety continuum—from violence prevention and organizational responses to violent acts to providing resources and supports to social workers who experience acts of violence. Therefore, agencies that employ social workers should demonstrate their ability to address issues of safety for their staff. Social work employers must protect their employees by instituting policies and procedures that maximize safety and security in the office as well as in the field. A culture of safety includes the following:

1.2
- Safety Policies that
  - provide an oral and written commitment by agency leadership to promote the safety of all staff, including support, paraprofessional, and professional staff
  - govern the management of dangerous (or hostile and violent) behavior in the workplace (including clients, coworkers, and supervisors)
  - establish safety teams or safety committees to ensure adherence to policy and procedure, as well as to provide peer support during and after an incident.
- A Safety Committee that
  - oversees the conscientious adoption, use, and ongoing review of the agency's policies that underscore the commitment to safety for staff, clients, administration, and governing boards
  - ensures that safety protocols that are instituted, updated, and practiced regularly
  - identifies and investigates physical measures and technology that contribute to and promote the safety of social work staff
  - provides ongoing proactive risk assessment that identifies line staff at risk for violence, precarious settings and working conditions, as well as orientation and in-service training on practices that can reduce or minimize or eliminate factors associated with elevated risk.

1.4

1.1 – Data Management and Reporting – 1.3.

- Data Management and Reporting Activities that
  - develop and implement an incident reporting system to document and track instances of threats, acts of violence, and damage to property. The reporting system

should allow for analysis of data on type of incident, location, pervasiveness, and occurrence. 1.3

  - create a mechanism for reporting and collecting data on an ongoing basis on incidents of assaults, threats, and abuse that can be analyzed to inform the agency about the incidence and prevalence of violence to guide the development of safety protocols and allocation of resources. 1.3. 1.1
  - regularly gather and disseminate information about all safety risks including assaults, threats, and abuse and develop strategies for managing them via case consultations, training and education, and policy development. 1.1

Standard 2. Prevention (1)

The goal of organizations that employ social workers should be to create a culture of safety that adopts a proactive preventative approach to violence management and risk. DEFINITION

Interpretation

1.1 Prevention activities use available information to minimize the risk of future incidents of violence. Therefore, a preventative approach involves analyzing and understanding past incidents and determining actions that can circumvent or avoid their reoccurrence. In assessing past incidents, the following factors should be considered:

might be a code? on its own

- type of incident (for example, verbal threat/abuse, intimidation, attempted or actual physical assault, property damage, stalking)
- severity of the incident (costs to the well-being of people or organizations)

- physical health (bodily physical harm)
- mental health (psychological and emotional harm/toll/impact)
- financial (monetary costs to property, people, or organization)

- staff, clients, and witnesses involved in or witnessing the incident
- weaknesses/breaches of protocol or gaps in protocol or policies that facilitated/contributed to, or did not deter, the incident (procedural, environmental, errors in assessment or misunderstanding of the safety protocol)
- orientation and training needs of staff for risk reduction and safety promotion
- assessment of current safety measures and policies and gaps in protocols/procedures.

Standard 3. Office Safety

Social workers' office environments should promote safety for social workers and their clients.

Interpretation

The office/agency environment where social workers work should not only be safe, but should actively promote and encourage safe practices. These practices can include, but are not limited to

- working spaces that allow for social workers to exit easily in potentially violent situations
- access to alarm systems that can alert others to a safety risk or breach
- access to visually open meeting spaces ("Risk Rooms") or presence of another staff team member when meeting with a client who may be verbally abusive or aggressive

- restricted access to objects that may be used as weapons (for example, stapler, paper weights, scissors, molded plastic chairs or office décor that can be picked up and thrown, and so forth)
- secure entry and access (for example, monitored, restricted access security guard, metal detector screening, bulletproof glass)
- well-lit hallways that lead to employees' workspaces
- secure entrances to employees' workspaces that are separate from public spaces.

Standard 4. Use of Safety Technology

Organizations that employ social workers should use technology appropriately and effectively to minimize risk.

Interpretation

Risk assessments may highlight the need to introduce technology to minimize risk of harm to social workers. Although technology is not a solution in and of itself, it can be a helpful tool in establishing and maintaining a culture of safety. It is important that staff understand that technology will not prevent incidents from occurring. Technology is not a substitute for safety planning.

As with all technological tools, consideration should be given to their limitations as well as the legal and ethical implications of their usage. Successful use of technology relies on a comprehensive and inclusive approach to identifying an appropriate system(s), and a commitment by all staff to use it appropriately. Technology can augment, but not replace, other important and critical requirements in these

guidelines. Reliance on technology alone can promote a false sense of security.

The introduction of safety technology must be accompanied by clear protocols and training regarding the limits and proper usage, by whom, and under what circumstances. Technological tools to consider include the following:

- Internal alert systems that can be activated from panic buttons in offices
- Internal alert systems that can be activated from key fobs or other mobile devices
- Panic buttons that are linked to public safety departments (police, emergency rooms, fire departments)
- Mobile safety devices that may incorporate GPS and/or audio/video recording
- Personal safety devices (for example, silent panic buttons, identification card holder with audio monitoring)
- Security cameras where appropriate.

Clients and staff must be informed about the use of safety technology, particularly if audio/video recording is involved. Although clients need not be informed about the location of internal alert systems, the knowledge that safety technology is in use may deter violent behavior. However, under no circumstances should technology be used to intimidate or coerce clients or staff. Safety technology should not be used to "spy" on staff or to provide undetected supervision without staff members' knowledge.

Standard 5. Use of Mobile Phones

Social workers should be provided with mobile phones to promote their safety in the field.

Interpretation

Mobile phones have become a necessity for communication outside the office. Wherever possible, social workers should use agency phones, rather than personal phones, to reduce exposure of their personal information. Although excellent tools for communication, a mobile phone can also potentially escalate an aggressive situation, and the social worker should therefore use it in a sensitive and sensible manner. To increase the safety provided by a mobile phone, social workers should

- keep the mobile phone fully charged, have a replacement battery on hand for emergencies, and keep a phone charger in the car
- be familiar with limitations of cell phone coverage in areas where they may visit
- ensure that they know how to use the phone properly before going into the field
- keep emergency contacts on speed dial
- keep GPS-enabled mobile phone applications activated at all times while in the field
- agree on and use "code" words or phrases to help social workers convey the nature of threats to their managers or colleagues
- remember to not use handheld mobile phones while driving
- send text messages of knowledge of circumstances, instead of calling, but not while driving
- use the phone discreetly, so as not to inadvertently escalate a potentially volatile situation and to avoid becoming a possible target for robbery.

Standard 6. Risk Assessment for Field Visits

Social workers should assess and take steps to reduce their risk for violence prior to each field visit. (3) + (1)

Interpretation

Social workers who make visits to clients in the field may be subjected to a range of safety risks. Prior to each field visit, social workers should conduct a risk assessment that includes the following: DEF.

3.1

- **Assessment of environmental factors**
  - Does the worker have a complete and exact address of the visit, to avoid appearing lost or confused?
  - Does the neighborhood pose risks for violence?
  - Is the visit scheduled at a time of day that is more risky than other times?
  - Are there other factors that may pose a risk for violence or danger (weather or disaster conditions, extreme heat or cold, icy roads)?
  - Have any events occurred in the neighborhood within the last 48 hours that might increase risk (for example, homicides, abductions, robberies, drug raids,)?
  - Does the area have reduced reception for mobile devices (for example, tunnels, rural areas)?
  - Will identification of the social worker's agency (for example, vehicle logo) increase risk?
  - Are there groups or individuals in the path to the home or near the location of the visit?
- **Assessment of client's living space**
  - Does accessing the space require the use of an elevator or flights of stairs?

3.1

also involves a familiarity with the living spaces / background

  - Are common spaces well-lit and clean?
  - Are exits easily accessible?
  - Who is likely to be in the client's home during the visit?
    - children
    - parents
    - other relatives or friends
    - pets, including guard dogs
  - Is/are the client, family members, or friends of the client known to engage in criminal or dangerous activities in the home?
  - Is there an increased risk of disease, infection, or pests in the home environment?
  - Is the family known to have weapons? 1.1
- **Assessment of proposed work activities** 1.1 —
  - Will the social worker engage in high-risk activities during the visit (for example, removing a child, notifying of reduction in benefits, terminating parental rights, executing a civil commitment procedure, helping a domestic violence victim to a safe house, delivering other potentially unwelcome information)?
- **Assessment of increased risk due to client's condition** 1.1
  - Does the client have an active substance abuse problem, particularly with alcohol?
  - Does the client have a mental illness or personality disorder, particularly if untreated?
  - Does the client have a history of or frequent violence or threatening behavior?
  - Does the client have a communicable disease?
- **Assessment of worker vulnerability** 1.1
  - Working alone
  - Visible physical conditions that may increase vulnerability (pregnancy, disabilities, use of cane or walking aid)

- Lack of experience
- Appearing timid, vulnerable, lost, or confused
- Lax attitude and/or overconfidence
- Worker bias or stereotyping that causes over- or underreaction to safety threats
- Attire (wearing jewelry and other valuables, high-heeled shoes, and so forth) that adds to vulnerability
- Accessories (political buttons, religious jewelry) that may trigger reactions
- Appearance (for example, tattoos, body piercings) that cannot be covered and that might attract/increase attention
- Lack of a safety plan

- Assessment of condition of emergency equipment that may be needed
  - Vehicle in good repair and working condition
  - Mobile device fully charged
  - Two-way radio working
  - Emergency telephone numbers available.
- Discussion of the issue of safety with the client, formally as a mutual safety contract or informally as a discussion of mutual safety

Social workers should be well trained in the use of their agency's risk assessment instrument and supervised to ensure consistency in practice. When a risk is identified, a safety plan must be created and adapted as necessary throughout the case planning and a plan of action to reduce or minimize that risk implemented. When the risk is determined to be too great or is unable to be minimized through the use of normal precautions, the social worker should have the opportunity, with management support, to state his or her concerns and develop an alternative plan until the risk is minimized by changed conditions or when appropriate support is available. The appropriate support may include being

additional safety precautions.

accompanied by a colleague or supervisor, being accompanied by law enforcement, changing the day or time of the visit, changing the site of the visit to a safer venue, or postponing the visit.

- vehicle checks
- client checks.
- surroundings check.

Standard 7. Transporting Clients

Social workers should acknowledge particular safety concerns when transporting clients.

Interpretation

When transporting clients is an expectation of the job, employers should ensure that policies and practices are in place to protect both social workers and clients. At the time of pick up, the social worker should assess

- the client's level of agitation (if any), use of intoxicants, and the meaning of the appointment to the client
- the possibility that the client has a weapon
- their own perception of a safety risk.

The social worker should also assess the condition of the vehicle:

- Is the interior of the vehicle free from potential weapons (for example, pens, pencils, magazines, books, handheld devices, hot beverages)?
- Is the vehicle in good working condition (for example, ample gas, working brakes, headlights/tail lights)?
- Is the vehicle equipped with proper safety equipment in case of an emergency (for example, flares, battery cables, spare tire)?

4.2

When transporting a child, the social worker should

- engage the child safety locks in the vehicle
- know the proper use and installation of a child safety seat that is appropriate for the child's age and size
- use a "buddy system"—that is, have a second social worker in the vehicle when transporting a client.

If the client is assessed to be unsafe to transport, or the vehicle is assessed to be unsafe to operate, agency policies should prohibit the social worker from transporting the client.

1.3

Standard 8. Comprehensive Reporting Practices

Social workers should engage in comprehensive reporting practices regarding field visits.

Interpretation

Social workers who are in the field should ensure that their in-office setting (supervisor, manager, coworker) is aware of their whereabouts and plans. This information should be kept in a secure location, accessible only to staff who need it. Prior to each field visit, social workers should

- provide addresses of visit and appointment times in the order they are scheduled
- provide information about the clients being visited
- indicate the length of each visit (estimated arrival and departure times)
- provide information about the vehicle they will use (license number, make, model, color)
- report change of plans to their supervisor or designated agency representative (for example, if a visit or appointment is canceled or delayed)
- provide information on how to reach them (for example, cell phone)
- carry agency identification cards at all times

5.

Following each visit, the social workers should report should report back to their supervisor or designated agency representative when the meeting is concluded or as soon as it is safe to do so. Social workers should also report completion and progress toward the next scheduled appointment or close of business. Agencies should develop and strictly adhere to systems that support verification of worker safety in the field and establish that social workers have safely completed their visits. If a social worker misses an appointment, these systems should be activated to track the social worker and ensure her or his safety. If normal contact cannot be made with the social worker in the field, agencies should develop policies about when law enforcement is called.

5

Standard 9. Post-Incident Reporting and Response

Employers of social workers should develop protocols that follow an incidence of violence or abuse.

Interpretation

DEF.

Despite an agency's best intentions, every incidence of violence or abuse cannot be anticipated or avoided. However, employers of social workers have an obligation to develop policies and protocols following a violent or abusive incident that seek to ameliorate the current victim's condition and to avoid future

incidents. The protocols can include the following:

- Providing prompt assistance to the employee
- Assessing medical need and obtaining medical assistance
- Debriefing with staff and witnesses
- Completing an incident report that details the incident, where and when the incident occurred, who was present/involved, a description of injuries, factors contributing to the event and whether or not medical services were advised and/or accessed.
- Developing a safety plan in response to the incident
- Intervening with other clients and observers of the incident
- Addressing the caseload distribution of the affected social worker
- Offering technical and legal assistance as needed
- Providing financial compensation for damage to property
- Offering voluntary referrals to Employee Assistance program services
- Following up on safety plan
- Implementing a quality assurance review of policies and procedures

Standard 10. Safety Training

Social workers should participate in annual training (or case supervision as needed) that develops and maintains their ability to practice safely.

Interpretation

Safety training can include skill building in risk assessment, risk management, risk reduction, a

previously constructed Safety Plan of Action that includes exit strategies, verbal de-escalation techniques, effective strategies for clinical interventions with violent or potentially violent clients, and nonviolent self-defense and the impact of secondary trauma. Safety training can also include the use of safety technology devices and advocating for self-care to effectively manage secondary trauma in the workplace.

Standard 11. Student Safety

Social workers need to be prepared for safe social work practice during their student years.

Interpretation

As practicum experiences are an important part of the social work curriculum, schools of social work are responsible for ensuring that social work students are educated about concepts and techniques related to safety as well as supervised in safe environments. Social work safety should be part of the curriculum/training of field practicum instructors. Schools should place students in settings with sound safety policies and procedures that should be reviewed with students in school and in the placement setting. Professional safety should be part of the school and agency orientations. Students who have safety concerns about their placements must be provided with supports until the safety concerns have abated. If the concerns cannot be adequately addressed, the student must be offered an alternative placement.

References

National Association of Social Workers. (2008). *Code of ethics of the National Association of Social Workers*. Washington, DC: Author.

U.S. Department of Labor, Bureau of Labor Statistics (2012). *Occupational outlook handbook*. Retrieved from www.bls.gov/ooh/community-and-social-service/social-workers.htm

Whitaker, T., Weismiller, T., & Clark, E. (2006). *Assuring the sufficiency of a frontline workforce: A national study of licensed social workers* [Executive summary]. Washington, DC: National Association of Social Workers.

NASW
National Association of Social Workers

NATIONAL ASSOCIATION
OF SOCIAL WORKERS
750 First Street, NE
Suite 700
Washington, DC 20002-4241
202.408.8600
SocialWorkers.org

Social Worker Safety: Tips and Training for Social Workers

February 16, 2021

View all blog posts underArticles | View all blog posts underOnline Master of Social Work

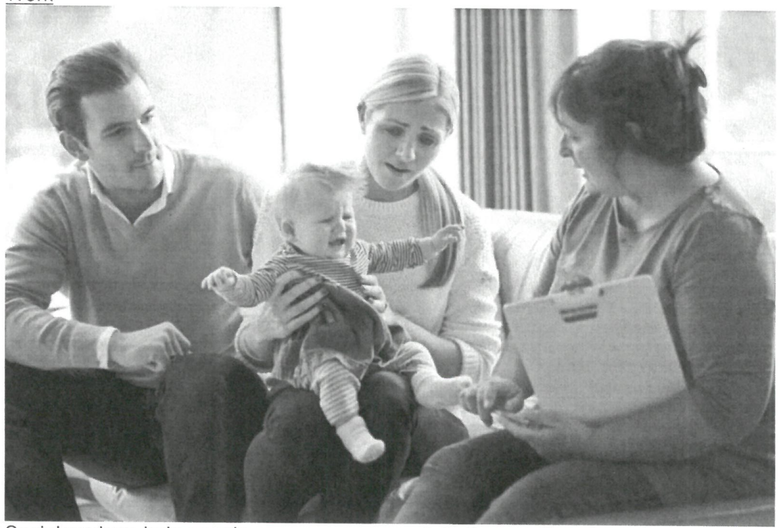

Social workers help people overcome personal challenges, from homelessness and poverty to domestic violence and children's behavioral issues. Although the career can be deeply rewarding, social workers can be at risk of physical and emotional violence, threats, and verbal abuse in their work.

Def of SW

Knowing the potential risks involved in the profession is essential. Equally important is an understanding of the laws and regulations designed to protect social workers. For example, a key aim of the Protecting Social Workers and Health Professionals from Workplace Violence Act of 2019 is to fund safer workplace measures for social workers. Promoting social worker safety in the workplace plays a critical role in attracting individuals to be part of a rewarding career that helps society's most vulnerable.

Why Is Social Work a Potentially Dangerous Profession?

Social workers must remain levelheaded even in high-stress encounters, such as emotionally charged situations, to minimize conflict. However, sometimes difficult circumstances can lead to physical acts of violence against a social worker. The National Association of Social Workers (NASW) reports that social workers and related occupations are "nearly five times as likely to suffer a serious workplace violence injury than workers in other sectors."

Is social work dangerous? It can be. Consider examples of potentially hazardous scenarios.

Removing a Child from an Unsafe Environment

Social workers may be involved in cases of physical and mental abuse, neglect, or mistreatment of children. After conducting investigations and interviews with family members, they must assess whether a home environment endangers a child's health and welfare. If an environment is determined to be unsafe, a social worker may recommend that a child be separated from the family. Parents may react with violence, insults, or threats upon hearing that decision.

Working with Individuals with Mental Illness

Although most people with mental health issues are nonviolent, some may react to what they see as a social worker's interference with physical threats or verbal abuse.

Visiting Clients in Risky Environments

Vulnerability driven by socioeconomic concerns can create potentially dangerous situations for social workers. For example, social workers may need to visit clients alone in at-risk neighborhoods. Social workers may also work in environments such as prisons and juvenile detention facilities where they can be exposed to violence.

Keeping People Healthy

A key part of a social worker's responsibility is to support people with their health care issues. From helping individuals navigate the complex health system to providing lifesaving information during outbreaks such as the COVID-19 pandemic, social workers regularly interact face-to-face with clients. In their efforts to help keep people safe, social workers may place themselves in danger of contracting transmittable diseases.

What Types of Violence Do Social Workers Encounter?

Social workers may face physical assault, including getting kicked, punched, or being attacked with a weapon. Additional acts of violence endangering social workers' safety include attempted assault, property damage, and threats. Verbal abuse may include being made fun of, getting cursed at, or receiving relentless insults.

Social workers working with children and families can experience a higher rate of violence than those in other fields. Child welfare caseworkers have to make difficult decisions about children's safety, potentially putting them at risk. For example, parents with a history of domestic abuse may react violently to a social worker's assessment. Abuse can also come from teenagers with a history of violence, putting social workers at risk of being hurt physically, psychologically, and emotionally.

Safety Tips for Social Workers

The laws that protect social workers from various types of violence combined with pre-visit planning and strategies to de-escalate dangerous situations can help social workers' safety.

Pre-visit Planning

Social workers should inform supervisors before meeting with clients. It may be necessary to alert security or law enforcement professionals for clients known for their unpredictability and violent history. Additional pre-visit plans include the following:

- Ensure that clients know your arrival time and be specific about the purpose of your visit to prevent them from being surprised or startled.

- Get to know the neighborhood of the home you plan to visit. Before arriving at your appointment, use Google Maps to familiarize yourself with the area, including locating the nearest police station.
- Provide clear information to your employer about your whereabouts. Share details about who you'll be visiting, specifying the time and the planned length of your visit.
- Be prepared with code words or phrases that alert your employer and colleagues to an emergency or a dangerous situation.
- Use a mobile phone app such as BSafe that by touch or voice activates an SOS alert, including your location and live GPS tracking.

De-escalation Tips

Even with the best-conceived plans, a sudden conflict may arise. Strategies for de-escalating stressful, confrontational, or violent scenarios may not control others' reactions, but can lead to a peaceful resolution. Social workers are focused on empowering individuals to overcome some of life's biggest challenges. With patience, a calm demeanor, active listening, and compassion, social workers can also help clients respond to a crisis mindfully. Below are examples of strategies that can be used to de-escalate tense encounters with clients:

- **Stay calm and listen actively.** Sometimes clients just need to be reminded that social workers are there to help.
- **Demonstrate empathy and be nonjudgmental.** Clients under immense pressure and with traumatic experiences may not always respond reasonably. Nonetheless, instead of pointing out flaws in their reactions, understand where they come from rather than judging them, and show grace under pressure.
- **Keep a safe distance.** In a high-pressure situation, the space between two people at odds gets smaller. By creating space, you not only protect yourself but also improve the chances of avoiding an escalation.
- **Be aware of body language.** Kind and empathetic words can be useful in de-escalating a situation. By matching your words' thoughtfulness with your voice, body language, and facial expressions, you help diffuse defensiveness.

The Importance of Personal Safety Training for Social Workers

Social workers make difficult decisions, such as denying benefits that may impact a client's financial well-being. This can lead to physical acts of violence or verbal abuse against them. Personal safety training for social workers can help social workers prevent and address the challenges. The following sections contain examples of safety tips for social workers.

Building Resilience

Social workers receive training to help them handle emotionally charged situations. However, stress can take a serious toll. According to a study in the journal *Child Abuse & Neglect*, the consequences for child protection workers can be psychological, manifested as fear or nightmares; organizational, causing low motivation or sick leave; and clinical, which can result in avoidance and emotional detachment from clients. Problem- and emotion-focused coping strategies are "significant predictors of resilience," according to a study in the journal *International Social Work*. Problem-focused coping strategies help alleviate stressors, while emotion-focused coping strategies help individuals manage their emotions. The study found that education programs can help social workers manage stress more effectively using these coping skills.

Social Worker Safety Policies and Training

Agencies and organizations that hire social workers can implement policies, procedures, and systems to promote safety. For example, agencies can provide easy access to alarm systems that alert colleagues of a safety risk. Other strategies include requiring open meeting spaces visible to more than one other person in the office and restricting access to objects that can be used as weapons.

In addition to providing safe environments for social workers, workplace safety training adds another layer to social worker safety. Personal safety training helps social workers understand and prepare for dangerous scenarios.

Training programs can cover communication-related concepts, such as speaking calmly with a clear and direct approach. Often, clients who become violent are angry at the situation, not the social workers, and social workers who react without becoming defensive are more likely to successfully navigate an encounter. Training may emphasize that telling a client to calm down may trigger a negative response, so a calm demeanor is more likely to successfully de-escalate a situation.

Risk assessment is another critical part of safety training. For example, situational awareness can include assessing a client's history, including with previous social workers. Other areas covered in training may consist of engaging in crisis communication, identifying rage and triggers, and treating violent clients.

Social Worker Home Visit Safety Strategies

Social workers should approach adults and children differently. Rapport building is vital to establishing trust with parents. The strategies for establishing rapport include the following:

- Approaching adults with an open mind
- Finding out what's important to adults
- Listening to an adult's explanation of the situation without correcting them or being argumentative
- Asking open-ended questions in which adults are allowed to offer their perspectives

Straightforward communication, setting visitation expectations, and describing their role in the family dynamic help clarify the process's ambiguities. Social workers must also provide parents with a sense of control by inviting them to participate in planning and scheduling.

Building trust is also at the heart of a social worker's approach to children, especially those who have faced violence, abuse, or neglect in the home. Children experiencing adversity such as abusive or incarcerated parents or families struggling with substance abuse may have feelings of anger, shame, and trauma. Social workers must validate children's emotions and encourage them to share their feelings in an open, honest environment, offering:

- Clear and honest interactions
- Respect
- Opportunities to share concerns and wishes
- Clear options and choices, which reduce anxiety and opposition when children are asked questions

Another vital aspect of social worker home visit safety strategies is cultural competence. Successful social workers are committed to cultural understanding and respecting the diversity of their clients. This understanding helps social workers earn their clients' trust, resulting in improved outcomes.

Consider additional safety strategies and techniques when visiting a client at home.

Dress Appropriately with Minimal Jewelry

Wearing proper attire facilitates ease of movement, and minimal jewelry makes social workers less of a potential target. Placing valuables in the car's trunk before driving to a client's home means not attracting attention.

Be Mindful of Pets

Social workers should call ahead of an appointment to check if a family has pets. If a social worker is allergic, alternative plans can be made. In some homes, pets can also be victims of violence, abuse, or neglect, increasing the chances that they can be dangerous.

Keep a First-Aid Kit in Your Car

A first-aid kit can be useful if a social worker is physically harmed during a visit. In the case of a bruise, cut, or injury, this handy health resource can help a social worker care for themselves and avoid infection or further injury.

Trust Instincts When Sensing Danger

Social workers should be mindful of their surroundings, familiarize themselves with clients' neighborhoods, be sensitive to warning signs, and trust their instincts.

Ask Permission to Hold or Handle a Child

Parents often feel vulnerable during visits, and feeling a lack of control may make them aggressive toward a social worker. A social worker should never assume that parents are OK with others holding a child, but should always ask permission.

Keep Personal Information Private During a Home Visit

Part of building rapport includes sharing information that makes others feel comfortable, but it's never a good idea for a social worker to share personal information that can put them and their loved ones at risk. Social workers should ensure that their personal information is kept private.

Carefully Approaching Important Situations

Throughout the U.S., communities, especially in rural areas and inner cities, lack access to critical social services and health resources. Social workers work in schools, hospitals, prisons, government agencies, and nonprofits to help individuals, families, and communities overcome adversity.

While social work is rewarding, it is potentially dangerous. The Centers for Disease Control and Prevention (CDC) reports that of the 20,790 workers who experienced traumatic workplace violence in 2018, 73% of them worked in health care and social assistance roles. Social worker safety is worth considering when pursuing a career in the field.

Ohio University's online Master of Social Work program prepares graduates to understand how social needs and policies affect populations. It also prepares them with a comprehensive awareness of the impact of social work values and ethics. Graduates receive real-life experience in helping individuals from rural and marginalized communities overcome life's biggest challenges. Learn more about how Ohio University can help you pursue a meaningful career as a social worker.

Recommended Readings

Career Spotlight: Marriage and Family Therapist
The Complicated Role of Alcohol in Crimes of Abuse and Domestic Violence
Social Workers Reducing the Impact of Poverty

SOURCES:

BBC, "Social Work: 'I Had a Parent Screaming Foul Names at Me'"
BSafe, Features
Centers for Disease Control and Prevention, Occupational Violence
Child Welfare Information Gateway, Domestic Violence and Worker Safety
Crisis Prevention Institute, "How to Stay Safe During Home Visits"
National Association of Social Workers, Infectious Diseases
National Association of Social Workers, Social Work Safety
National Association of Social Workers, Social Worker Safety
National Association of Social Workers, Massachusetts Chapter, Workplace Safety
SAGE Journals, "Do Stress and Coping Influence Resilience in Social Work Students? A Longitudinal and Comparative Study from India"
ScienceDirect, "Violence Against Child Protection Workers: A Study of Workers' Experiences, Attributions, and Coping Strategies"
Social Work Today, "Violent Crime and Social Worker Safety"
U.S. Bureau of Labor Statistics, Social Workers
Washington State Department of Social and Health Services, Cultural Competence

JANUARY 2020

NASW National Association of Social Workers

ISSUE BRIEF

Protecting Social Workers and Health Professionals from Workplace Violence Act of 2019 (S. 2880/H.R. 5138)

BACKGROUND

Tragic incidences of violence against social workers and health professionals in the workplace are increasing. Between 2011-2013, there were 23,000 workplace assaults, and nearly 75% of these were in healthcare and social service settings.[1] In 2018, the Bureau of Labor Statistics found that health and social service workers were nearly five times as likely to suffer a serious workplace violence injury than workers in other sectors.[2] Social workers and health care professionals provide essential mental health and health services to individuals and groups in a wide variety of settings such as hospitals, home care agencies, child welfare departments, community-based clinics, and schools. Social workers face unique vulnerabilities at work, as they typically provide services outside the four walls of an office, such as in client homes and community-based settings.

Far too many social workers and health professionals have lost their lives to workplace violence. The alarming statistics do not capture the substantial number of unreported assaults, which, according to one survey, are as high as 85% of all assaults.[3] A 2004 national study by the National Association of Social Workers of 10,000 licensed social workers found that 44% of the respondents reported facing personal safety issues in their primary employment setting and 30% felt that their employers did not adequately address safety issues.[4]

Addressing this growing epidemic of workplace violence is a key success factor in reducing provider burnout and increasing retention. Promoting workplace safety is also critical to ensuring a sufficient mental health and health workforce. This issue is especially acute in the many geographic areas where there is a severe shortage of qualified mental health providers or health providers. As our nation strives to build a skilled workforce to meet client and patient needs in shortage areas, promoting workplace safety will go a long way in attracting social workers and health care professionals to be a part of this workforce. Finally, preventing workplace violence is also essential in providing a healing environment for clients. When clients witness violence in these settings, it can set back treatment months, if not years.

POLICY SOLUTION

The bipartisan *Protecting Social Workers and Health Professionals from Workplace Violence Act of 2019 (S. 2880/H.R. 5138)*, will promote safer working conditions by establishing a grant program within the Department of Health and Human Services to fund the implementation of workplace safety measures. The grants may be used by States, Indian Tribes, Tribal organizations, and urban Indian organizations to deploy safety equipment (such as security cameras and GPS locators), make facility improvements, implement safety training programs, and provide support services for professionals who have been victims of violence. The bill provides $10,000,000 per year, to be awarded over 5 years.

This legislation was introduced by Senator Kyrsten Sinema (D-AZ) – who is also a social worker – and Senator Lisa Murkowski (R-AK), as well as Representatives, Julia Brownley (D-CA-26) and Elise Stefanik (R-NY-21).

---

[1] Occupational Safety and Health Administration, US Department of Labor. (2016). *Guidelines for Preventing Workplace Violence for Healthcare and Social Service Workers.* Retrieved from www.osha.gov/Publications/osha3148.pdf

[2] Bureau of Labor Statistics, U.S. Department of Labor. *Occupational Outlook Handbook, Social Workers.* Retrieved from www.bls.gov/ooh/community-and-social-service/social-workers.htm (visited January 03, 2020).

[3] American Federation of Government Employees. (2016). *Violence Against Health & Social Service Workers.* Retrieved from www.afge.org/article/violence-against-health-care--social-service-workers-must-end/

[4] Whitaker, T., Weismiller, T., & Clark, E. (2006). *Assuring the sufficiency of a frontline workforce: Executive summary.* Washington, DC: National Association of Social Workers. Retrieved from www.socialworkers.org/LinkClick.aspx?fileticket=ESTCCZA4HAE%3d&portalid=0

For more information contact:
Dina Kastner, MSS, MLSP
dkastner.nasw@socialworkers.org » 202.336.8218

SYRACUSE UNIVERSITY
COLLEGE OF HUMAN ECOLOGY
SCHOOL OF SOCIAL WORK

SOCIAL WORKER SAFETY TIPS

(1)

2.1

WORK ARRIVAL:

informing caleagues + planning.

- Organize before leaving home
- Visually check parking lot when you arrive
- Are suspicious vehicles or persons in the lot?
  - Obtain plate number if possible
- Vary your parking
- Do not park next to van or truck
- Do not accept unsolicited offers of assistance
- Do not unlock the door to admit stranger
- Sound your horn if menacing stranger remains around your vehicle
- Cellular phone – call 911 assistance
- Be alert and aware of surroundings
- Visually check building when arrive
- Have key or swipe card ready
- Do not converse with strangers

3.1

PLAN FOR THE VISIT

- Notify office of destination with the name, address, phone number, time of visit, and reason for the visit
- Notify client your are coming and purpose of the visit / meeting
- Obtain specific directions
- Have a street guide / map in car
- Bring cellular phone if available
- Call office before entering home
- Establish office procedure if you do not call in
- Try to conduct home visit in pairs, if possible
- Wear clothes and shoes that provide freedom of movement

3.2

TRAVELING TO SITE:

(4) Transporting / travelling.

- Do not keep things out in open in car
- Lock purse / valuables in car prior to leaving for visit
- Keep car in working order with at least ½ tank of gas
- Drive with doors locked
- Park in well-lit, visible area and lock the car doors (do not park in the driveway or directly in front of the house)
- Observe the premises for suspicious activity; listen for threatening sounds
- Listen to your body language, if you feel unsafe at anytime, LEAVE
- Do not slam car doors
- Do not walk on lawns
- Knock and use doorbell
- Give eyes a chance to adjust to light (before entering)
- Carry as little as possible into home
- Return to car with key ready, check front and back seat and floor before getting in
- If you are being followed, do not drive home; drive to nearest police or fire station and honk your horn or drive to open gas station or business where you can safely call the police
  - Do not leave the car unless you can walk into the building safely
- If possible, have a cellular phone in your car for emergencies; it may save your life!
- Always carry small flashlight with you (in purse or car)

(3) 3.3.

DURING THE VISIT:

- Visually check others present during visit
- Assess person's emotional state
- Avoid sitting in the kitchen (many possible weapons; boiling water, knives)
- Always wait to be invited to sit
- Sit in straight-backed chair (easier to rise from chair in a hurry)
- Be aware of all possible exits in the house
- If there are dogs in house, ask client to remove them due to your allergies
- Ask to turn the TV off, as "I" have trouble hearing
- Restate the goal of the visit
- Maintain a respectful and courteous attitude
- Respect the individual's personal space (maintain an appropriate distance)
- Keep a clear path to the door
- Avoid positioning self so that you become trapped if needed to make quick exit
- If situation escalates, LEAVE
- Avoid giving out personal information, such as address or phone number
- Visually check the surrounding area or parking area when leaving
- ALWAYS carry car keys in same place where they are readily accessible

(2.) 2.4.

OFFICE VISITS:

- Plan an emergency escape route
- Beware of how to gain help and to call law enforcement
- Know if 911 can be called directly or if other numbers must be called first
- Arrange furniture in office to prevent entrapment (sit where you have quickest access to the doorway)
- Avoid working alone in office after regular working hours; notify someone you are working late and keep all exterior doors locked
- Avoid seeing clients alone after hours
- Have co-worker attend meeting when interviewing possibly threatening clients or if you feel your personal safety might be threatened
- Keep desk and office clear of objects that could be used as a weapon (store letter opener in desk drawer)
- Avoid giving personal information during interview
- Keep your automobile locked at all times

(1)

DEFUSING TECHNIQUES:

1.5

- Keep it from escalating; try to stay calm and listen attentively
- Avoid sudden movements
- Avoid confrontation
- Maintain eye contact and personal space
- Keep situation in your control
- Use calm tone when speaking
- Do not argue with person
- Signal a co-worker or supervisor that you need help (try not to let angry client see this, as it may escalate situation)

Information collected from:

Joe McAnally,
Montana Department of Public Health and Human Services;

AMRIC Associates Limited,
Investigation and Security Consulting;

De-Escalating Volatile Situations Seminar presented by Carolyn Miller

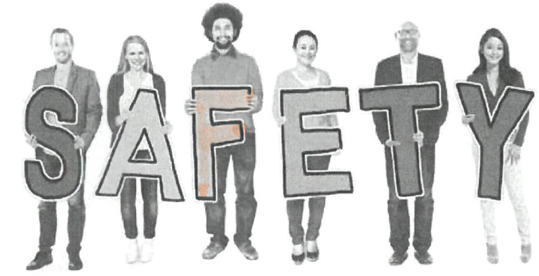

Safety Awareness for Social Workers: Tips Learned Along the Way

by Veronica Hardy, Ph.D., LCSW, CCTP

It was the 1990s, a time when cell phones barely existed and I had just transitioned from using a typewriter to a word processor. It was my second field placement in the graduate social work program. One of my responsibilities was engaging in home visits with adolescent males who were court committed to a residential facility for delinquent behaviors. During that first home visit in Philadelphia, PA, my field supervisor gave me a few tips while we were pulling into a parking space. He told me to avoid placing my bag on the floor, to view my surroundings upon entering the home, and never to sit with my back to the door. I was to always position myself in a way that I could get out in case of an emergency or threatening situation. These are tips that I practiced for each successive home visit.

This field placement eventually became a job offer when I graduated. Over my time there, I received more tips. For example, I was taught to "fit in" with my surroundings in the way I dressed. I was told to avoid walking with a noticeable briefcase that would signal I was an outsider there for official business. I was encouraged to sign out the company car rather than using my own, so the agency would know my whereabouts. If the car did not return by the end of the workday, that was a signal that something was wrong.

A couple of years later, I moved to another residential facility as a social worker/therapist. The children were ages six through 13. They were there for multiple reasons, such as serious behavioral concerns, mental health diagnoses, or traumatic family events. I had the role of providing individual counseling, case management, and home visits to help facilitate reintegration. I enjoyed working with this age group and learned more tips for safety along the way. I was again encouraged to use the agency vehicle. This agency had cell phones—the original bulky gray versions—that we could sign out. We were also encouraged to pair up with a colleague and conduct home visits on the same day. This way, we would have support. These tips were great for a novice master's level social worker.

Over the years, I kept collecting and practicing tips—even those that would enhance my safety within my office space. These included never "boxing yourself in" the office, meaning I should never block myself from the door with a desk or other objects. I learned to keep my desk clear of objects that could be grabbed by another person and used as a weapon against me, such as scissors. Another tip was to avoid wearing a lanyard keychain around my neck, as someone could easily grab it and cut off my breathing. In addition, I was advised to request panic buttons on office phones to enhance workplace safety. The list of tips kept growing, and each had a valuable role as I practiced social work.

When I reflect back, I realize that the knowledge of my field supervisor and colleagues strengthened my safety awareness while engaging in social work practice. As social workers, we are encouraged to practice self-care methods, and I have found safety practices to be an important self-care component. Now that I am a social work educator, I have taken steps to prepare students through integrating safety topics into their classes. This has included discussing experiences that other social workers have had regarding violence in the office, in parking lots, or during home visits. Although rare, it is important for students to know what has happened, what could happen, and ways to decrease the chances of experiencing harmful events.

One semester, a student even jokingly asked me, "Dr. Hardy, are you trying to get us to leave the program?" This was followed by a "thank you" for sharing such information so they could be prepared for circumstances that they had not previously considered.

Another semester, I had the opportunity to invite a self defense expert to a social work practice class. The students were very engaged as he taught them multiple techniques for transitioning out of a threatening situation, including ways to de-escalate by using their words. It was great to see students in their first semester of an MSW program gain knowledge about safety measures in the early phase of their education. This knowledge could then be used in their upcoming field placements and careers in social work.

I tend to encourage students to self-educate, ask questions, and read articles outside of class requirements. Their semesters are often busy with balancing course work, family, employment, and other life responsibilities. I empathize with this through my own educational transitions and question, "What would have prompted me to learn more about social worker safety throughout my education?" More than likely, it would have taken one of my professors prompting me to learn about the topic through sharing social worker safety experiences. Considering this question has been an influential factor toward integrating this topic into courses I facilitate, posting information on the bulletin boards in the social work department, and using the announcement feature of online course systems to provide news and practice updates.

I have also found that one of the most valuable sources of information is the National Association of Social Workers website. In 2013, NASW published a document titled "Guidelines for Social Worker Safety in the Workplace." This document communicates several of the same tips I have learned throughout my career and is a very succinct compilation.

Social work is a promising and respectable profession. It is necessary to also realize the unpredictability of circumstances. Both novice and seasoned social work professionals should continue gaining and communicating knowledge about safety in practice. This is yet another way to continue strengthening the profession and valuing those who fulfill such an important role in our society.

Resources

National Association of Social Workers. (2013). Guidelines for social worker safety in the workplace. Retrieved from *http://www.naswdc.org/practice/naswstandards/safetystandards2013.pdf.*

NASW. (2014). Social work safety. Retrieved from *http://www.naswdc.org/pressroom/events/safety1006/default.asp.*

Veronica Hardy, Ph.D., LCSW, is a certified clinical trauma professional and assistant professor of social work at the University of North Carolina at Pembroke. She has focused on mental health among various populations while providing services through residential treatment facilities, group homes, and private practice. She is a recipient of the Outstanding Teaching Award at UNC Pembroke, which recognizes faculty who are distinctive in all areas associated with teaching. Dr. Hardy is an anti-human trafficking advocate.

Social Worker on the Shelf

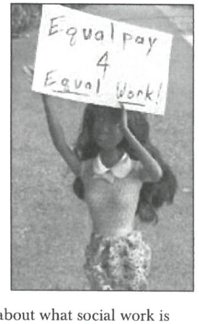

According to her Instagram account, Social Worker on the Shelf is sent by the state board elves every December to magically show the skills/knowledge/ethics of a competent social worker.

"Linda" started as an idea I had one day after class talking with my wife. I started her in response to the "Elf on the Shelf" trend. I wanted a way to distract my students from finals and to do something kind of silly to reinforce some of the things we had discussed all semester. Linda actually turned into something a little more meaningful. First of all, she has been a catalyst for discussion on social media between people who may not have the same values as social workers. Second, she has helped educate people, both social workers and non-social workers, about what social work is and what we do. I think Linda will become a mainstay in December for years to come.

Read more about the Social Worker on the Shelf project on *THE NEW SOCIAL WORKER's* website at: *http://www.socialworker.com/feature-articles/technology-articles/social-worker-on-the-shelf-what-barbie-taught-me-about-feminism/*

Stephen Baldridge, Ph.D., LMSW
Abilene Christian University

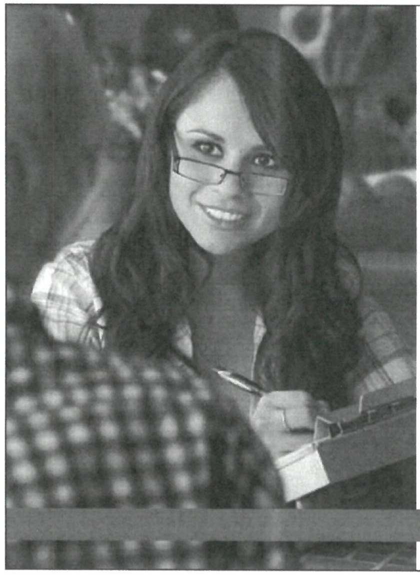

100% ONLINE MASTER OF SCIENCE IN
SOCIAL WORK
Educate. Empower.

Specializations (optional) available online:
» Psychosocial Oncology
» Gerontology
» Mental Health
» Alcohol and Drug Counseling
» Military Social Work

UNIVERSITY OF LOUISVILLE. KENT SCHOOL OF SOCIAL WORK

NOW ENROLLING FOR FALL 2016
APPLY TODAY!

Connect with us: uofl.me/newsocialworker
For program availability in your state visit uofl.me/sarstates

Copyright of New Social Worker is the property of White Hat Communications and its content may not be copied or emailed to multiple sites or posted to a listserv without the copyright holder's express written permission. However, users may print, download, or email articles for individual use.

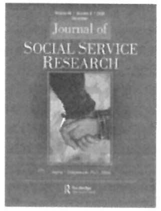

Journal of Social Service Research

ISSN: 0148-8376 (Print) 1540-7314 (Online) Journal homepage: https://www.tandfonline.com/loi/wssr20

Instrumental, Interpersonal or Holistic: Social Work Managers' Conceptions of Safety in the Psychosocial Work Environment

Kettil Nordesjö

To cite this article: Kettil Nordesjö (2020) Instrumental, Interpersonal or Holistic: Social Work Managers' Conceptions of Safety in the Psychosocial Work Environment, Journal of Social Service Research, 46:6, 789-800, DOI: 10.1080/01488376.2019.1658690

To link to this article: https://doi.org/10.1080/01488376.2019.1658690

© 2019 The Author(s). Published with license by Taylor & Francis Group, LLC.

Published online: 12 Sep 2019.

Submit your article to this journal

Article views: 1195

View related articles

View Crossmark data

Full Terms & Conditions of access and use can be found at https://www.tandfonline.com/action/journalInformation?journalCode=wssr20

JOURNAL OF SOCIAL SERVICE RESEARCH
2020, VOL. 46, NO. 6, 789–800
https://doi.org/10.1080/01488376.2019.1658690

Routledge
Taylor & Francis Group

OPEN ACCESS | Check for updates

Instrumental, Interpersonal or Holistic: Social Work Managers' Conceptions of Safety in the Psychosocial Work Environment

Kettil Nordesjö

Centre for Work Life and Evaluation Studies, Malmö University, Malmö, Sweden

ABSTRACT

Social work managers are accountable for risks and safety in the psychosocial work environment. This article aims to understand how social work managers deal with safety in the psychosocial work environment in social service organizations with potentially conflicting logics of regulation and security, by answering the questions: How do social work managers conceptualize safety in the psychosocial work environment?; What are the implications of different conceptions of safety in the psychosocial work environment for social work management? Through a qualitative phenomenographic analysis of semi-structured interviews with 27 managers in the Swedish social services, three conceptions were found: an instrumental, interpersonal and holistic conception. As each conception encompasses the former and thus increases the level of comprehensiveness, tensions between the logics of regulation and security increases. Managers with a comprehensive conception must therefore reflect on the way regulations for safety may conflict with social relationships. Implications for social work management are the need to discuss how safety management relates to social work professionalism, and the self-regulation due to the integration of safety thinking in social work professionalization. Future research could investigate how the conceptions relate to managerial and professional practice and how different parts of the social services conceptualize safety.

KEYWORDS
social work management; safety management; psychosocial work environment; phenomenography

Introduction

Any explanation of the nature of social work is dependent on a close reading of risk as a significant feature of modern societies (Webb, 2006). Today, social work organizations are required to deal with risks in different areas. One area that has gained a lot of attention in recent years is the psychosocial work environment, where social work managers, in particular, are accountable for dealing with issues of excessive caseloads, moral stress and clients' threats on a daily basis.

This article investigates how social work managers in the social services deal with safety in the psychosocial work environment to handle such risks, in the presence of two different logics (Webb, 2006). In the first logic, *the logic of regulation*, social work managers enforce and implement external pressures of auditing and standardization, which regulate risk through tasks and procedures that are fundamental for an organization to operate, and correspond to the societal and political pressures of human service organizations (Garrow & Hasenfeld, 2010). On the other hand, in the second logic, *the logic of security*, social work managers must provide safety for their employees by establishing values and norms of health and safety in the workplace (Dollard & Bakker, 2010). Supportive management is important for social workers' retention (Frost, Hojer, Campanini, Sicora, & Kullburg, 2018), trust between managers and employees is fundamental (Törner, Eklöf, Larsman, & Pousette, 2013) and implementation of safety measures is best carried out bottom-up through the involvement of employees (Wikman & Rickfors, 2018).

CONTACT Kettil Nordesjö kettil.nordesjo@mau.se Linkedin: www.linkedin.com/in/kettilnordesjo Centre for Work Life and Evaluation Studies, Malmö University, Malmö 205 06, Sweden.

© 2019 The Author(s). Published with license by Taylor & Francis Group, LLC.
This is an Open Access article distributed under the terms of the Creative Commons Attribution-NonCommercial-NoDerivatives License (http://creativecommons.org/licenses/by-nc-nd/4.0/), which permits non-commercial re-use, distribution, and reproduction in any medium, provided the original work is properly cited, and is not altered, transformed, or built upon in any way.

Social work managers need to acknowledge potential tensions between these logics, since they may be in conflict with each other. Regulating the psychosocial work environment according to a logic of regulation may create calculation and suspicion and hinder trust in accordance with the logic of security, which is difficult to reestablish when violated (cf. Webb, 2006). This resonates with the increase of proceduralism and bureaucratization of social work practice that may lead to a more top-down controlling approach, which, impedes bottom-up participation, constrains the relationship between social workers and clients, and almost always result in more procedures to regulate practice (Jones, 2010). Similarly, risk management procedures may leave relational aspects of social work practice under-emphasized and under-theorized (Broadhurst, Hall, Wastell, White, & Pithouse, 2010). Also, focusing on conforming to external rules and routines for safety may affect social work practice by hindering flexibility and innovation, increasing a fear of making errors and creating distrust between managers and employees (Jerak-Zuiderent, 2012; Lawler, 2015; Törner et al., 2013). How social work managers deal with safety in the psychosocial work environment in an organization with two different logics may thus have implications for social work practice as well as the relationship with clients. In the remainder of this introduction, literature relevant to the management of risks and safety in social work is discussed. The introduction ends with the article's aim and research questions.

Literature Review

The management of safety in social work implicitly relates to risks. The traditional quantitative risk analysis assumes an objective and true level of risk that can be assessed through standardized techniques. But what is seen as dangerous and an acceptable risk in the psychosocial work environment might vary between contexts and is influenced by social processes and cultural patterns (Antonsen, 2009). For example, risks can be relevant to self-identity as a part of the social work profession (Kemshall, 2010) where one has to "take it". Safety, on the other hand, refers to a situation where a statistical risk is deemed acceptable, a feeling of security and control, and constitutes a practice that aims at reducing the likelihood of hazardous events (Antonsen, 2009).

While risk management refers to the calculation of risks in a logic of regulation, research on safety climate stresses that it is impossible to make rules for every situation. Therefore, it is better to create a climate where people feel confident to assess situations and act accordingly in order to follow and improve both rules, and to enhance their skills to cope with problematic situations (Neal & Griffin, 2002). A "good" safety climate is thus not only about making people aware of rules and procedures, but about making them acknowledging new problems of safety and taking initiatives to handle such problems that have not yet been addressed in rules and procedures (Tholén, Pousette, & Törner, 2013). This contrast between compliance and participatory safety behavior resonates with several dichotomies in the safety literature (eg. from safety-I to safety-II, Hollnagel, 2014). Wikman and Rickfors (2018) argue that safety work may either be implemented top-down from managers to employees through rules and standards (stability) or implemented bottom-up (adaptability). Where stability is built on individual conformity to rules and routines and is undermined by improvization, adaptability presupposes employees' varying ways of performing tasks as the foundation of safety work. Still, it is not a question of either/or; organizations with unpredictable events and non-routine tasks need to be decentralized in order to adapt to local challenges, but still require some sort of centralization to be managed as a system (Wikman & Rickfors, 2018). The recent construct, psychosocial safety climate (PSC), is an example of proactive structures for safety in the work environment, created bottom-up with the participation of employees (Dollard & Bakker, 2010). Such initiatives must be promoted through stable management support in order to be translated from an espoused to an enacted safety climate (Yulita, Dollard, & Idris, 2017). In sum, the safety literature generally argues for a bottom-up and adaptability perspective in the management of safety in the workplace. Still, a top-down and stability perspective through management support is acknowledged to ensure a systemic approach in organizations.

The two perspectives of the safety literature resonate with the character of social work management, where managers are forced to deal with both organizational and professional ideals and tasks. A results-based, task-oriented leadership needs to be balanced with a process and people-oriented leadership where professionals are supported (Schmid, 2010). There are thus two sides to social work management. On the one hand, management is broadly associated with efficiency, regulation, bureaucratic processes and administrative duties (Shanks, 2016). Consequently, while social work practitioners value professional individualized judgement for its flexibility and responsiveness to individual factors, social work managers value predictable knowledge for its consistency and accountability (cf. Hafford-Letchfield & Lawler, 2013; Kemshall, 2000; Webb, 2006). On the other hand, social work managers will not necessarily show loyalty and commitment to the organization rather than the profession. They often have a social work background, see themselves as social workers rather than managers (Evans, 2011), and are as much recruited and valued by their employees for their social work competence as for their managerial skills (Shanks, 2016). Like their employees, they have discretionary space and may be considered street-level bureaucrats with "a certain leeway in defining the organizational conditions of policy work achieved by street-level workers" (Hupe, Hill & Buffat, 2015, p. 325). This is also suggested to hold true for senior managers (Evans, 2016).

Hence, social work managers are not necessarily characterized by, and limited to, managerial levels and may take different positions (cf. Shanks, Lundström, & Wiklund, 2015). In the context of safety in the psychosocial work environment, these positions may allow them to manage safety corresponding to stability or adaptability, a compliance or participatory safety behavior, and more generally, the logics of regulation and security.

Aim and Research Questions

Although research has highlighted dualities in safety management and how social work managers may take different managerial positions, little research has combined the two research strands and addressed how social work managers approach the management of safety in the psychosocial work environment in organizations with potentially conflicting logics of regulation and security. One way to investigate this issue is to study social work managers' conceptions of safety. Depending on these conceptions, managers may differ in approaches to the formulation and implementation of safety management, and subsequently social workers' strategies (cf. Lambley, 2010). Exploring managers' conceptions is essential for elucidating what support is required in implementation (cf. Mosson, Hasson, Wallin, & von Thiele Schwarz, 2017).

The aim is to understand how social work managers deal with safety in the psychosocial work environment in social service organizations with potentially conflicting logics of regulation and security. This is done by interviewing 27 social work managers on different levels in the social services in a large Swedish municipality. The research questions are:

1. How do social work managers conceptualize safety in the psychosocial work environment?
2. What are the implications of different conceptions of safety in the psychosocial work environment for social work management?

Next, the research methods are described and the results are presented: social work managers' conceptions of safety in the psychosocial work environment. These three conceptions are then discussed in relation to the presented dualities and logics, followed by a conclusion.

Methods

In order to describe how managers conceptualize safety in the psychosocial work environment, a phenomenographic approach was used (Marton, 1981). It starts from the understanding of a problem or the situation to be dealt with in order to understand how people deal with it – in this case safety in the psychosocial work environment (Avby, Nilsen, & Abrandt Dahlgren, 2014). There are a limited number of qualitatively different ways to understand and make sense of a phenomenon. These *conceptions* signify the relationship

Table 1. Interviewees in relation to district, unit, level and experience as manager.

Manager characteristic	Number of interviewees				Sum
District	*1*	*2*			
	19	8			27
Unit	*Child welfare*	*Social assistance*			
	13	13 (+1 HoDi)			27
Management level	*Deputy head of unit (dHoU)*	*Head of unit (HoU)*	*Head of department (HoDe)*	*Head of district (HoDi)*	
	13	11	2	1	27
Years of experience as manager	*0-1*	*2-5*	*6-10*	*10-*	
	6	7	6	8	27

between what is conceived and how it is conceived (Sandberg, 2000). Phenomenography involves description rather than interpretation, aims to achieve conceptual rather than topical description, and investigates differences rather than similarities (Dahlgren & Fallsberg, 1991).

Sample

The study was carried out within the social services departments of two city districts in a Swedish municipality. In each district, managers from two different units of the departments were interviewed: child welfare and social assistance. In one of the districts, managers working with addiction and debts were also interviewed. The units are both people-processing and people-changing (Hasenfeld, 1983), meaning they both categorize clients for other parts of the social services and work with face-to-face social work to improve clients' situations. Particular to the Swedish context is the way in which the Social Services Act does not give much guidance in individual cases, giving managers and caseworkers extensive discretion in individual assessments. Similar to an American (Kim & Kao, 2014), European (Frost, Hojer, Campanini, Sicora, & Kullburg, 2018) and Swedish (Tham & Meagher, 2009) pattern for social workers, both areas have a high caseworker turnover, indicating potential work environment problems.

The districts were chosen due to their shown interest in the standardization of social work and managers' psychosocial work environment in an earlier research project. Department managers were contacted and an invitation to an interview was sent to 32 managers on all levels in the districts' units, of which 27 agreed. As other phenomenographic studies have shown, 20 interviews is an adequate target in order to find varying conceptions (e.g. Sandberg, 2000). Managers differed in level and years of experience (see Table 1). In general, the higher the managerial level, the longer the experience. All interviewees had a bachelor in social work and experience of social work practice. It should be noted that deputy heads of units do not formally have staff liability but supervise professionals and are a link to professional practice. Still, several managerial duties and tasks are delegated to them, which makes them both qualified and significant to contribute to the overall aim of the study. In all, the variation in units, districts, managerial levels and experience ensured a variety of social service managers.

Instrument

A semi-structured interview guide was used with the themes *background*, *management and leadership*, *psychosocial work environment* and *risks and safety*. For this phenomenographic article, three questions were essential:

- "What does safety in the psychosocial working environment mean for you in your workplace?"
- "How do you manage and influence safety in the psychosocial work environment?"
- "What are the challenges to managing safety in the psychosocial work environment?"

The questions were thus open-ended in order to leave it to the interviewee to define, reason and reflect, but structured in order to capture both definitions and action in relation to safety in the psychosocial work environment. The interview guide was pilot tested and adjusted in another municipality prior to the study.

Procedure

Managers were interviewed individually by the author during one month in early 2018. Interviews took place

Table 2. Social work managers' conceptions of safety in the psychosocial work environment.

Aspect / Category	A. Perception of basis for safety	B. Description of relationship between risks and safety in social work	C. Description of managing for safety in the psychosocial work environment	D. Description of when, where and how questions of safety are discussed in the workplace
1. *Holistic*	Support structures	Social work is unpredictable. Reflections on and ambivalence to conflict between relational and regulative ideals.	Create structures for the unexpected. Create sense of joint responsibility. Involve employees.	Create climate to encourage discussions on safety. Case meetings. Build capacity through education and professionalization.
2. *Interpersonal*	Relations	Social work is morally and emotionally stressing. Society is tougher and less solidary today. Relations with employees and clients are fundamental.	Be observant to identify stress. Dialog with employees. Be a role model. Collegial support.	Relieve and listen. Discussion ex post. Informal discussions.
3. *Instrumental*	Regulations	There is little knowledge about clients. Physical surroundings can handle external threats. Risks as threats and violence.	Emphasize rules and routines. Individual responsibility. Managers' responsibility ends with reminders.	Workplace meetings. Discuss plan against threats and violence. Introductions in technology and physical surroundings.

in interviewees' workplaces and lasted 40–90 minutes. They were recorded, transcribed verbatim, and organized in the Nvivo 11 software. The study has been subject to ethical review and approved by a Swedish regional ethical review board (ref 2017/816). Information about the study and interview was sent in advance, and informed consent was obtained from all interviewees.

Data analysis

The seven-step process of phenomenographic analysis by Dahlgren and Fallsberg (1991) was used (cf. Avby et al., 2014):

1. The transcripts were read in their entirety to get acquainted with them.
2. Significant statements by the interviewees relating to the research question were marked.
3. The statements were then compared in order to find similarities and differences. This resulted in four different aspects of safety in the psychosocial work environment, i.e. different individual ways to describe the phenomenon: basis for safety, the relationship between risks and safety in social work, managing safety, and when, where and how questions of safety are discussed.
4. Statements with similarities were then grouped in empirically based categories as conceptions of safety in the psychosocial work environment.
5. Similarity between statements was explored and described in tandem with the aspects from step three.
6. When sufficiently clear, the categories were labeled the instrumental, interpersonal and holistic category. They are delimited conceptions of managing safety in the psychosocial work environment and do not represent individual qualities among interviewees.
7. Categories were hierarchically ordered in an outcome space by searching for relationships between them. The hierarchy indicates that a higher category encompasses the lower categories. There is thus an increasing comprehensiveness from the instrumental to the interpersonal and finally the holistic conception, where the latter contains elements of the former two.

The seven-step process is not to be followed strictly but allows for an interplay between the steps (Dahlgren & Fallsberg, 1991).

Results

The phenomenographic analysis resulted in three categories, i.e. qualitatively different conceptions of safety in the psychosocial work environment (see Table 2). The three categories are described in the remainder of this section, starting from the lowest category, *the instrumental.*

The Instrumental Conception

The first category emphasizes rules and regulations to handle risks and ensure safety. Managers' primary concern is to make sure employees

follow routines for safety. The relationship between risks and safety in social work is the unpredictability and lack of knowledge of new clients, which demands safety measures in terms of physical surroundings and alarms. Managers' signatures on diverse documents make them more visible, and some managers have been physically hurt some time during their careers. A characteristic experience is that threats and violence have become more common and have come closer, not least through social media:

> It's easier to send threats today through emails and to post something on Facebook. [Clients] record conversations [with caseworkers] and post them on the web and make fun of them […] Caseworkers find it uncomfortable … this just didn't exist before. Or they post entire investigations that you've written. People read them online and say, "Oh, such worthless investigations, this caseworker is incompetent, she does bad assessments" and so on … it didn't happen before. (HoDe 1)

Managing safety is about giving employees information and reminding them about wearing personal alarms from the reception in client meetings and during home visits in child welfare. It is crucial for employees to be updated on policies, plans, rules and routines concerning threats and violence. The individual responsibility is emphasized:

> I can remind them constantly, but it is still the responsibility of every employee to know about those routines that apply in the workplace on safety, privacy, safety and risk assessment. Constantly checking that they're doing the right thing is impossible. I'm thinking they're adults working here who should be aware of these routines when it comes to these very serious things and follow them. I expect everyone to follow them. I might be stupid but I can't expect less because then I would have to walk around and be worried all the time, you understand what I mean? (dHoU 11)

One senior manager who takes this view is aware that not all employees follow the routines that make managers responsible for incidents:

> We've said that now people have to sign agreements so that we know they've read [the routines]. Should anything happen, us managers are responsible for everyone having read them and understood them. That's something you have to continue to work with, when you deal with threats and violence. (HoDe 1)

Joint discussions on questions of safety in this category take place at workplace meetings where updated routines or plans against threats and violence are presented. If something happens, routines are revised. Managers can use introductions to show how physical surroundings and technology work in practice to minimize risks:

> Today I had a new employee and we went down to the reception and into a visitor room and talked about how to act when you have a [client] visit. How should you be placed? "Your visitors should sit here and then you take that chair and then you have the door there and then you have the alarm there." On a very practical level. […] I'm clear with the fact that I give the conditions, but if you do not "wear your protective clothing" and something happens … my responsibility ends here, I can't control anything beyond this. (HoU 19)

In sum, safety in the *instrumental* category starts from the idea that there is little knowledge about new clients. They can get violent, so it is important to use routines and technology in the physical surroundings to carry out social work. Since safety is based on individual conformity to rules, managers need to remind employees and even obtain written agreements of the acknowledgement of rules and routines.

The Interpersonal Conception

In the second category, relationships between people are the basis for maintaining safety. Managers must be attentive of their employees' stress levels and have an open dialog in case something happens. As this category encompasses the previous category, rules and routines are not absent. But they are seen as a safety net and a structure to be toned down, since it in itself can contribute to threats and violence. For example, the relationship between risks and safety in social work is described as people becoming pressured and unpredictable due to social workers' interventions in their lives. One manager relates clients' unpredictability to a lack of communication between caseworkers and clients and between caseworkers and other public actors that could lead to denied applications:

> We've worked a lot with communicating to the client that if you don't go to the [municipal job center] and

you get a rejection, you need to call the client and ask how it happened. "We have talked about this, if you don't participate, you don't have the right to social assistance." To communicate more. It's safety thinking, the importance of the client's understanding so that a rejection does not come as a surprise. If you become fuzzy and afraid of being clear, then you will create unnecessary situations. (HoU 15)

Safety in the psychosocial work environment is thus not as much a question of prediction and regulation of external threats, but of good relations with clients. One manager in social assistance relates safety to a climate of trust where employees are not afraid to speak out but feel safe with each other and with their managers. Burnout is not only a result of high caseloads, but of relationships:

> If you have very good relationships in a workplace then the risk of burnout is reduced. Because there is somebody who catches you when you fall. The feeling of safety and comfort at work. And especially when working with vulnerable people as we do. It gets under your skin, people feeling bad, other actors who want us to do more and clients' parents who call because their children haven't received money. That's why you need to have a feeling of safety. I also think that in situations of threats and violence … the worst part is if you don't dare to talk about what you have experienced because it is expected of you to handle certain things. That you should be tough and so on. It is important to me that you do not end up in such a climate. (dHoU 13)

Managers should be attentive of the work environment and excessive demands among employees. The characteristic way to discuss questions of safety is in the dialog between manager and employee, mostly informally but also in staff appraisals. Some managers describe how they can sense that something is wrong and that it is important to be able to adjust the workload if caseworkers show signs of emotional stress:

> I try to keep track, and I tend to be pretty good at noticing… I can notice when someone seems to be down, and I try to say, "How's it going?" – "No, it's nothing." – "Okay" – "I just want to ask, because I get a feeling that maybe there's something, but if you say that it's nothing, it's completely okay." I have no problem backing off if someone says so. But it's important to try to read your employees. (HoU 16)

In sum, in the *interpersonal* category, social work intervenes in people's lives, and they can become pressured and unpredictable. Safety needs to be based on relations and dialog whether it concerns clients, employees or managers. Rules and regulations are not trivial but are a safety net rather than something that handles daily risks.

The Holistic Conception

In the third category, safety in the psychosocial work environment starts from the idea that some people are unpredictable, so social work organizations must be prepared and have support structures in place. Unpredictability is thus not something that can be regulated in detail as in the instrumental category, but has to be accepted and handled through support structures, joint responsibility and social work professionalization. The relational aspect of social work is complemented by unpredictability:

> There are things that I can't influence … which are risks. And it's because we are working with people. And they may be erratic and unpredictable in their behavior. And I think that we work with the most delicate issues that people absolutely don't want to share with anyone else. And it's our task to talk about those things. Or to be curious about them and how the client ended up in this situation. And that's where I think of safety. When people are cornered, it results in aggression or threats or such things. It's an interaction. Some get scared, others don't. (dHoU 3)

There is also an ambivalence to the conflict between relations and regulations, since the unpredictability still must be handled in some rule-based way:

> I think that social work, where you think good about people and want to see what's positive in everyone, can tone down threats. You might think "That wasn't that dangerous?", or that you are uncomfortable asking someone entering behind you at the staff entrance: "Can I see your card because I don't recognize you?", or: "It's just us two at this meeting, I don't need any alarm". And there I must, while endeavoring to see people's possibilities and have a positive attitude towards service users, not only be a realist but also a pessimist with regard to safety. If something happens then we should have done everything we could to prevent it. […] But it's partly in conflict with the social change work… not in conflict with the exercise of authority… but with being caring and having a relationship. (HoU 18)

Similarly, there are statements where managers reflect on the counterproductive effects of rules and regulations, such as whether caseworkers should show their personal alarm that they wear in client meetings or not, since showing them might provoke the client. A characteristic of this category is thus the presence of both regulations and relations that makes managers ambivalent to how safety should be approached. However, the holistic category is more than the sum of the previous two categories. The basis for safety is different support structures which permit managers to handle the unexpected. One is to create a sense of shared responsibility. Besides following rules and routines, they should actively participate in the improvement of safety. One manager speaks about anchoring issues among employees, "to discuss how things should be done and how work should be organized" (HoU 9). A frequently described way to do this is to address safety during "case meetings" where caseworkers discuss current cases, thus providing a stress-reducing structure to caseworkers. Another manager describes the importance of weekly group meetings where every caseworker has a chance to tell the group about case difficulties and other work-related problems. Even if caseworkers do not speak out about work environment problems, "I get a sense of how they cope" (dHoU 4). Safety must be kept alive not only through ad hoc dialog or updated regulative measures; as in the previous two categories, they presuppose and enhance each other:

> I think the question of safety is alive, but people must be reminded of it constantly. Just as with all obvious routines, one must keep talking about them. We often react in completely different ways than we thought we would, especially in the case of threats and violence. Something that has happened to you that didn't affect you much in the moment may return much later, and then it's important for me that I, as a manager, step in at the beginning and exaggerate the support, rather than let it drop. (HoU 16)

Support structures for social workers can also be seen as competency built through ongoing vocational training and introductions. This is especially important since half of one unit's caseworkers got their social work degree in the last year. Integrating issues of safety in introductions thus relates safety issues to social work professionalism.

In sum, the *holistic* conception starts from the idea that people are unpredictable, so employees must be anticipative through participation and support structures.

Discussion

The results have shown how social work managers conceptualize safety in the psychosocial work environment in three different ways. In this closing section, the three conceptions are discussed through the logics of *regulation* and *security*, as well as what their implications are for social work management.

Conceptions between the Logics of Regulation and Security

Where the instrumental category has ties to the logic of regulation, the interpersonal category lies closer to the logic of security. And at the top of the hierarchy, the holistic category may be understood as the interaction of the two logics. It is also here that tensions between logics are most visible.

In the instrumental category, the way to handle the unpredictability of social work is to calculate and plan the work environment in a way that minimizes risks, most often conceptualized as external threats and violence. Managers implement safety measures top-down and it is important to remind, and demand written approval from, individual employees to conform to the prescribed procedures. This resonates with features of the logic of regulation such as the proceduralism and standardization of social work where the aim is to promote uniformity and predictability of tasks and procedures (cf. Ponnert & Svensson, 2016; Timmermans & Epstein, 2010). The category represents the risk assessment side of safety management where there is an assumption of risks "out there" that can be estimated using standardized techniques (cf. Antonsen, 2009). A notable unintended consequence is that the discovery of risks, as the potential provocation of clients through visible alarms, may

highlights new aspects of the workplace that can be modified and regulated.

In contrast to the instrumental conception, the interpersonal conception is based on the reflection that the social services in themselves play a role in potential threats and violence from clients. Safety in the psychosocial work environment must therefore begin in the relationship and trust between manager and employee, and employee and client. Managing safety in this category is not primarily about calculating risks, but creating trust and safety in relationships with other people. This resonates with the logic of security where managers provide security for their employees through trust in social relations. However, risk prevention is related to interpersonal dialog and the managerial observation of employees, making the conception ad hoc rather than anticipating, ex post rather than ex ante. In a safety management perspective, there is thus a lack of prevention through formal structure, participation and bottom-up strategy in this category, making it vulnerable to the contingencies of social work. It is noticeable that even though the interpersonal conception encompasses the instrumental conception, there is little built-in conflict between the two in the interpersonal conception. While rules and routines are a safety net and taken for granted structure, they are not the decisive way for handling safety and should instead be toned down.

Conflict between logics is instead most noticeable in the holistic conception. As the highest category encompassing the former two categories, the holistic conception of safety relates to both logics. On the one hand, there is a focus on creating and regulating structures of different kinds to handle the unpredictability of social work. On the other hand, these structures operate through relations and trust. Although it may seem like the "best of both worlds", there is an ambivalence and discussion in this category relating to the fact that regulations and relations can be in conflict with each other. As Webb (2006) argues, regulation creates calculation and suspicion and hinders trust, which is difficult to reestablish when violated. The ambivalence in the holistic category points to a reflective yet fragmented stance that leaves a manager undecided on whether what you are doing as a manager is the right way to go in a social work context where the relationship with the client is often seen as fundamental to success. However, Webb also argues that the logics can reinforce each other, as when mechanisms of trust, such as discussions of safety among colleagues, are integrated in formal structures or social work professionalization such as introductions. If done in a participatory manner, this resonates with research on safety management and climate (e.g. Dollard & Bakker, 2010; Törner et al., 2013).

In sum, social work managers' conceptions of safety in the psychosocial work environment can be understood in the increasing comprehensiveness from a logic of regulation, to a logic of security, and finally in the interaction and potential conflict between them. The tension between logics is thus increasing with the comprehensiveness of social work managers' conceptions. This has implications for social work management.

Social Work Managers and Safety Management

The conclusion that the tension between logics increases with managers' comprehensiveness, implies that managers with a more holistic and reflective stance towards safety in the psychosocial work environment may be more aware of the potential contradictions involved in the implementation of safety management. As seen in the holistic conception, the question is not whether to regulate or not, but rather how different aspects of safety management can harmonize with actual social work practices. Safety management in social work thus requires a continuous discussion on social work professionalism. In light of the recent debate on standardization where social work is said to have become practiced in line with the goals and values of the organization rather than the professional (Evetts, 2010; Ponnert & Svensson, 2016), it could therefore be argued that questions of safety could also risk being dealt with in an organizational top-down perspective. From a safety research perspective, such a development would be unproductive. Since not all aspects of all possible situations can be regulated, and would be too complex to grasp, there is a need to give

professionals decision-making skills within their discretionary space in order to take on the unpredictability of everyday social work. This resonates with an adaptive perspective of safety management implementation where safety measures are developed and adapted to the actual practice performed by employees (Wikman & Rickfors, 2018).

Furthermore, a feature of the holistic conception is the way safety management is integrated with social work professionalization such as introductions. This raises the question whether risks and safety are a valid part of social work managers' and professionals' curricula. On the one hand, topics relevant to the everyday practice of employees in human service organizations are relevant for social work education to problematize and put in perspective. For example, in the leadership literature, it is acknowledged that developmental practices should be adapted to the needs and roles of managers (Day, Fleenor, Atwater, Sturm, & McKee, 2014). On the other hand, integrating questions of safety in social work management and professionalization implies the institutionalization of thinking in terms of risks and safety in social work practice. This may be a form of self-regulation of social work, an integral feature of the logic of regulation and the audit society, where actors eventually do not need externally imposed rules to conform to certain behaviors, but instead regulate themselves (cf. Power, 1997; Webb, 2006). In management practice, it takes the most comprehensive conception of safety to reflect on this dilemma. In this sense, critical ongoing reflection regarding the relationship between regulations and relations may be of benefit to social workers and managers when engaging with safety issues in the psychosocial work environment.

Limitations and Strengths

Another result is that safety in the psychosocial work environment is most often associated with avoidance of clients' threats and violence. Indeed, the social services has traditionally been exposed to threats and violence in the workplace due to the face-to-face work with vulnerable clients in exposed situations (e.g. Padyab & Ghazinour, 2015; Robson, Cossar, & Quayle, 2014). However, this may also be a limitation in the research design. Clients' threats and violence were often the first things interviewees associated with *safety*, possibly leaving other problems in a broader psychosocial work environment unmentioned. Another limitation is that the results do not depict an actual social work management practice. Although the phenomenographic approach starts from conceptions in order to understand how people deal with the problem being conceptualized, the research design sheds little light on how social work managers manage safety in practice. Nontheless, since *understanding* is a fundamental factor for successful implementation (Lundquist, 1987), the description of conceptions and how they relate to each other is an important part of analyzing conditions of implementation.

Conclusion

In conclusion, social work managers conceptualize safety in the psychosocial work environment as instrumental, interpersonal or holistic. As the conceptions increase in comprehensiveness, they also increase in terms of contradictions and potential conflicts between the logics of regulation and security. A holistic conception challenges manager to reflect on and critically assess the way in which regulations for safety may conflict with relations in a social work organization. This has two implications for social work management. First, safety management must be discussed in dialog with social work professionalism in order to harmonize with actual social work practices. Second, as safety management may become integrated with social work professionalization such as introductions and create self-regulative elements, it may take the most comprehensive conception of safety to critically reflect on the relationship between regulations and relations when engaging with safety issues in the psychosocial work environment.

These key findings resonate with research on safety and social work management. The duality and ambiguity of social work management (Schmid, 2010; Shanks, 2016; Shanks et al., 2015) may contribute to different conceptions that range from the top-down regulatory management

of risks to the more recent approach seen in research on safety climate (Törner et al., 2013; Wikman & Rickfors, 2018). Furthermore, the findings elucidate in-built tensions between perspectives on safety essential to the understanding of managerial conditions for implementation (cf. Mosson et al., 2017).

Future research could investigate how the conceptions relate to safety management in practice. As research has shown, an espoused psychosocial safety climate on a managerial level does not equal an enacted climate in a certain operational domain (Yulita et al., 2017). For example, will the tensions between the logics of regulation and security that stands out in the holistic conception be in conflict or reinforce each other, and how can managers handle or achieve such situations? And in what ways are managers relating safety management to a professional social work practice? Also, different areas of the human services may have their own safety issues to deal with. Future research could compare managers' conceptions of safety in different areas of the human services. Elderly care has a longer history of working with standardization and patient safety and could provide insights into how the logics of regulation and security have been handled by managers (cf. Törner et al., 2013).

Acknowledgments

The author would like to thank Sandra Jönsson for comments on this article.

Disclosure statement

No potential conflict of interest was reported by the author.

Funding

This work was supported by the Swedish Research Council for Health, Working Life and Welfare under Grant 2016-07220.

ORCID

Kettil Nordesjö https://orcid.org/0000-0003-3565-6563

References

Antonsen, S. (2009). *Safety culture theory, method and improvement*. Boca Raton, FL: CRC Press.

Avby, G., Nilsen, P., & Abrandt Dahlgren, M. (2014). Ways of understanding evidence-based practice in social work: A qualitative study. *British Journal of Social Work, 44*(6), 1366–1383. doi:10.1093/bjsw/bcs198

Broadhurst, K., Hall, C., Wastell, D., White, S., & Pithouse, A. (2010). Risk, instrumentalism and the humane project in social work: Identifying the informal logics of risk management in children's statutory services. *British Journal of Social Work, 40*(4), 1046–1064. doi:10.1093/bjsw/bcq011

Dahlgren, L.-O., & Fallsberg, M. (1991). Phenomenography as a qualitative approach in social pharmacy research. *Journal of Social and Administrative Pharmacy: JSAP, 8*(4), 150–156.

Day, D. V., Fleenor, J. W., Atwater, L. E., Sturm, R. E., & McKee, R. A. (2014). Advances in leader and leadership development: A review of 25 years of research and theory. *The Leadership Quarterly, 25*(1), 63–82. doi:10.1016/j.leaqua.2013.11.004

Dollard, M. F., & Bakker, A. B. (2010). Psychosocial safety climate as a precursor to conducive work environments, psychological health problems, and employee engagement. *Journal of Occupational and Organizational Psychology, 83*(3), 579–599. doi:10.1348/096317909X470690

Evans, T. (2011). Professionals, managers and discretion: Critiquing street-level bureaucracy. *British Journal of Social Work, 41*(2), 368–386. doi:10.1093/bjsw/bcq074

Evans, T. (2016). Street-level bureaucracy, management and the corrupted world of service. *European Journal of Social Work, 19*(5), 602–615. doi:10.1080/13691457.2015.1084274

Evetts, J. (2010). Reconnecting professional occupations with professional organizations: Risks and opportunities. In L. Svensson, & J. Evetts (Eds.), *Sociology of professions: Continental and Anglo-Saxon traditions* (pp. 123–144). Göteborg, Sweden: Daidalos.

Frost, L., Hojer, S., Campanini, A., Sicora, A., & Kullburg, K. (2018). Why do they stay? A study of resilient child protection workers in three European countries. *European Journal of Social Work, 21*(4), 485–497. doi:10.1080/13691457.2017.1291493

Garrow, E., & Hasenfeld, Y. (2010). Theoretical Approaches to Human Service Organizations. In Y. Hasenfeld (Ed.), *Human services as complex organizations*. Los Angeles: Sage.

Hafford-Letchfield, T., & Lawler, J. (2013). *Perspectives on management and leadership in social work*. London: Whiting & Birch.

Hasenfeld, Y. (1983). *Human Service Organizations*. Englewood Cliffs, NJ: Prentice-Hall.

Hollnagel, E. (2014). *Safety-I and safety-II: The past and future of safety management*. London: CRC Press.

Hupe, P. L., Hill, M., & Buffat, A. (Eds.) (2015). *Understanding street-level bureaucracy*. Bristol: Policy Press.

Jerak-Zuiderent, S. (2012). Certain uncertainties: Modes of patient safety in healthcare. *Social Studies of Science, 42*(5), 732–752. doi:10.1177/0306312712448122

Jones, R. (2010). Managing at a distance in social work and social care. *Social Work and Social Sciences Review, 14*(1), 59–75. doi:10.1921/095352210X662135

Kemshall, H. (2000). Conflicting knowledges on risk: The case of risk knowledge in the probation service. *Health, Risk & Society, 2*(2), 143–158. doi:10.1080/713670160

Kemshall, H. (2010). Risk rationalities in contemporary social work policy and practice. *British Journal of Social Work, 40*(4), 1247–1262. doi:10.1093/bjsw/bcp157

Kim, H., & Kao, D. (2014). A meta-analysis of turnover intention predictors among US child welfare workers. *Children and Youth Services Review, 47*, 214–223. doi:10.1016/j.childyouth.2014.09.015

Lambley, S. (2010). Managers: Are they really to blame for what's happening to social work? *Social Work and Social Sciences Review, 14*(2), 6–19. doi:10.1921/095352210X557592

Lundquist, L. (1987). *Implementation steering: An actor-structure approach*. Lund: Studentlitteratur AB.

Lawler, J. (2015). Motivation and meaning: The role of supervision. *Practice, 27*(4), 265–275. doi:10.1080/09503153.2015.1048056

Marton, F. (1981). Phenomenography – Describing conceptions of the world around us. *Instructional Science, 10*(2), 177–200. doi:10.1007/BF00132516

Mosson, R., Hasson, H., Wallin, L., & von Thiele Schwarz, U. (2017). Exploring the role of line managers in implementing evidence-based practice in social services and older people care. *The British Journal of Social Work, 47*(2), 542–560.

Neal, A., & Griffin, M. A. (2002). Safety climate and safety behaviour. *Australian Journal of Management, 27*(1_suppl), 67–75. doi:10.1177/031289620202701S08

Padyab, M., & Ghazinour, M. (2015). A comparative study of experiences of client violence and its impact among Iranian and Swedish social workers. *European Journal of Social Work, 18*(1), 129–139. doi:10.1080/13691457.2014.883367

Ponnert, L., & Svensson, K. (2016). Standardisation – the end of professional discretion? *European Journal of Social Work, 19*(3–4), 586–599. doi:10.1080/13691457.2015.1074551

Power, M. (1997). *The audit society: Rituals of verification*. Oxford: Oxford University Press.

Robson, A., Cossar, J., & Quayle, E. (2014). Critical commentary: The impact of work-related violence towards social workers in children and family services. *British Journal of Social Work, 44*(4), 924–936. doi:10.1093/bjsw/bcu015

Sandberg, J. (2000). Understanding human competence at work: An interpretative approach. *Academy of Management Journal, 43*(1), 9–25. doi:10.2307/1556383

Schmid, H. (2010). Leadership styles and leadership change in human and community service organizations. In Y. Hasenfeld (Ed.), *Human services as complex organizations*. Los Angeles: Sage.

Shanks, E. (2016). *Managing social work: Organisational conditions and everyday work for managers in the Swedish social services* (Doctoral dissertation). Department of social work, Stockholm University.

Shanks, E., Lundström, T., & Wiklund, S. (2015). Middle managers in social work: Professional identity and management in a marketised welfare state. *British Journal of Social Work, 45*(6), 1871–1887. doi:10.1093/bjsw/bcu061

Tham, P., & Meagher, G. (2009). Working in human services: How do experiences and working conditions in child welfare social work compare? *The British Journal of Social Work, 39*(5), 807–827. doi:10.1093/bjsw/bcm170

Tholén, S. L., Pousette, A., & Törner, M. (2013). Causal relations between psychosocial conditions, safety climate and safety behaviour–A multi-level investigation. *Safety Science, 55*, 62–69. doi:10.1016/j.ssci.2012.12.013

Timmermans, S., & Epstein, S. (2010). A world of standards but not a standard world: Toward a sociology of standards and standardization. *Annual Review of Sociology, 36*(1), 69–89. doi:10.1146/annurev.soc.012809.102629

Törner, M., Eklöf, M., Larsman, P., & Pousette, A. (2013). Säkerhetsklimat i vård och omsorg. Bakomliggande faktorer och betydelse för personalsäkerhet och patientsäkerhet [Safety climate in healthcare. Underlying factors and their importance for personnel safety and patient safety]. *Arbets- och miljömedicin*, Rapport 2013:1. Göteborgs universitet.

Webb, S. A. (2006). *Social work in a risk society: Social and political perspectives*. New York: Palgrave Macmillan.

Wikman, S., & Rickfors, U. (2018). Att förebygga hot och våld i statliga myndigheter-en jämförelse mellan två perspektiv på säkerhetsarbete [Preventing threats and violence in government agencies – a comparison between two perspectives on safety work]. *Arbetsliv i omvandling*, (2), 1–52.

Yulita, Dollard, M. F., & Idris, M. A. (2017). Climate congruence: How espoused psychosocial safety climate and enacted managerial support affect emotional exhaustion and work engagement. *Safety Science, 96*, 132–142.

THE ACADEMY FOR PROFESSIONAL EXCELLENCE

SAN DIEGO STATE UNIVERSITY SCHOOL OF SOCIAL WORK

LITERATURE REVIEW: WORKER SAFETY

PREPARED BY: EMILY SULPIZIO, MSW STUDENT (SAN DIEGO STATE UNIVERSITY)

FEBRUARY 2016

SACHS
Southern Area Consortium of Human Services

INSPIRING INNOVATIVE SOLUTIONS IN HEALTH AND HUMAN SERVICES

Table of Contents

Introduction 2
A. Safety in the Office 3
1. Arriving to Work 3
2. Preparing for Client Meetings in the Office 3
3. Creating Safe Interview Settings in the Office 3
4. Ensuring Security in the Office 4
B. Safety When Making Home Visits 6
1. Plan for Visit 6
2. Traveling to Site 8
3. During the Visit 9
4. Returning to the Office 12
C. Safety When Transporting Clients 14
D. Defusing Techniques 15
1. Preparing for a De-escalation Discussion 15
2. Body Language and a Worker's Physical Stance 16
3. Holding a Diffusing Conversation 17
E. Reporting Incidents and Employer Response 18
F. Establishing Committees 20
G. Worker Safety Trainings 21
H. Action by the Academy 25
References 27
Appendices 30
Appendix 1: Risk Factors for Violent Behavior 31
Appendix 2: Worker Safety Intervention Plan 32
Appendix 3: Guidelines for Utilizing Teamed Response (Buddy System) 34
Appendix 4: Social Worker Safety Competencies and Learning Objectives in a Child Welfare Context 36

Introduction

Social Workers are subject to risks of violence in various health and human service disciplines including child welfare, behavioral health, criminal justice, adult protective services, and domestic violence (NASW, 2013). Workplace violence is defined as harmful or disturbing acts or threats including physical assaults, badgering, or bullying at a place of occupation (OSHA, 2015). Violence in the workplace against social service workers may occur in a variety of settings including hospitals, nursing homes, residential treatment centers, group homes, community care settings, non-residential treatment centers, and field work settings (OSHA, 2015). Health and human service workers may face increased risk for workplace violence due to a number of reasons including the large number of individuals in need of social services, a decline in funding for services, limited staff, expanding caseloads, public disapproval of social services, fearful or angered clients, and a lack of understanding from the public of the purpose of social services (Newhill, 2012).

In addition to the physical pain from physical harm, subjects of workplace violence may also experience mental and emotional trauma, alterations in co-worker communications and connections, anxiety or concern to return back to work, worry of supervisor's judgement, or feelings of inadequacy, shame, depression and inferiority (Kim & Hopkins, 2015; OSHA, 2015). While employees experience trauma associated with workplace violence, incidents of violence in a workplace cause negative effects for health and human service agencies which may include a reduction of clients or consumers, heightened expenses regarding medical treatment for victims, diminished service capacity, and a risk for a poor or reduced reputation. It is essential for health and human service agencies to develop policies and procedures for preventing and responding to incidents of workplace violence to ensure the vital success of a workplace and limit the number and harmful effects associated with incidents of workplace violence (Kim & Hopkins, 2015).

Between the years of 2011 and 2013, approximately 74% of all workplace assaults in the United States took place in health and human service agencies (OSHA, 2015). Employee fear of workplace violence may lead an employee to experience both psychological and physiological disruptions which in turn may prevent positive functioning in the workplace (Kim & Hopkins, 2015). Currently, California, New Jersey, Washington and Kentucky are among many states who have established safety guidelines for health and human service workers (Kelly, 2010).

This report is designed to review protocols and procedures for ensuring workplace safety in health and human service agencies. The research included in this report consists of information from worker safety trainings and published worker safety guidelines with a few noted worker safety studies. The research will discuss tips for maintaining safety in an office setting, in public spaces, during home visits, and when transporting clients, as well as techniques for de-escalating potentially violent situations. Additionally, this report will discuss guidelines for reporting and responding to workplace incidents of violence and establishing safety committees, as well as current worker safety trainings provided to health and human service employees.

A. Safety in the Office

1. Arriving to Work

- Upon arriving to work, it is suggested for workers to remain in their vehicle and conduct a visual scan of the parking lot for unfamiliar or skeptical vehicles (Syracuse University School of Social Work, 2011).
  - If workers notice suspicious cars, they should take down the vehicle's license plate number (Syracuse University School of Social Work, 2011).
- When exiting the vehicle and approaching the office building, workers should scan the environment and check the outside of the building for safety before entering (Syracuse University School of Social Work, 2011).
- It is important for workers to have their identification badge and key in their hands before entering the building and to refrain from talking with unknown persons when walking into their workplace (Syracuse University School of Social Work, 2011).

2. Preparing for Client Meetings in the Office

- Prior to meeting with a client, workers should conduct a detailed clinical risk assessment while paying special attention to the client's past history of drug and alcohol use, violence, and use of weapons (Saturno, 2011)[1].
  - Analyzing a client's risk factors including any clinical, demographic, or biologic risk factors help to determine if a joint interview is required or necessary (Saturno, 2011; Taylor, 2011).
  - Workers should review any previous reports of documentation on the client and should assess for any notes of violence or threats towards the client's former therapists, if the client has been hospitalized in the past, if the client is currently taking medications, and if the client has access to weapons (Quinn & Mason, 2011).
- Late afternoon or evening interviews with clients should be reserved for well-known, nonviolent clients (Newhill & Hagan, 2010).
- Once appointments are made, workers should share their schedule with their co-workers and try their best to remain on schedule in order to prevent clients from having to wait (Newhill & Hagan, 2010).

3. Creating Safe Interview Settings in the Office

- All office settings should be safe for both the workers and the clients (NASW, 2013).
- Interview rooms and offices where meetings with clients take place should have protected entrances and be distanced from waiting rooms and public areas (NASW, 2013).
- Workers should examine their office for hazardous objects that could be used as a weapon (Newhill & Hagan, 2010; Taylor, 2011).
  - Objects commonly found in an office including a stapler, paper weight, office décor, fire extinguisher, letter opener, keys, pens, books, or scissors can readily be picked up, thrown, or used as a weapon by someone who is upset or angry (NASW, 2012; Nelson, n.d.).

[1] See Appendix 1: Risk Factors for Violent Behavior

        - These items must have restricted access and be hidden in a drawer during a client meeting to ensure safety (NASW, 2012; NASW, 2013; Syracuse University School of Social Work, 2011).
- Workers should arrange their office in a way that offers quick and easy access to the exit and is not confrontational to the client (Taylor, 2011).
- Positioning office furniture should be strategic in which it helps, not hinders, a worker during a hazardous situation. (NASW, 2012).
    - Workers should be sure there is a comfortable distance between themselves and their client and sit in a way that creates an equal balance of power to produce an environment that is non-threatening (Nelson, n.d.; Newhill & Hagan, 2010).
    - While many offices allow for the client to be nearest to the exit door, it is strongly suggested to place the worker's chair closest to the exit so the worker can leave in a hurry if necessary (NASW, 2012; NASW, 2013; Newhill & Hagan, 2010; Syracuse University School of Social Work, 2011).
    - It may also be helpful to place the worker's chair to face the exit to allow the worker a clear view of those who come in to the office (NASW, 2012). This strategy helps to avoid unsuspecting visitors who enter the office with no one aware (NASW, 2012).
- While many workers may shut their door during a client meeting to protect the privacy of their client, it is suggested for workers to allow the door to remain completely or partly open (NASW, 2012; Newhill & Hagan, 2010).
    - Keeping the door open when meeting with a client signals to the client that other workers in the office are on high alert, listening and watching (NASW, 2012).
    - It may also be helpful to have an additional exit out of the interview room if possible (Newhill & Hagan, 2010). All confidentially procedures must be ensured before following through with this safety protocol.

2.4

4. Ensuring Security in the Office

- In addition to hiding potentially dangerous objects and strategically positioning furniture in an office, it may be helpful to coordinate a safety buddy system and/or designate a 'safe-room' within the office for security purposes (NASW, 2012; NASW, 2013; Nelson, n.d.).
    - If meeting with a potentially violent client, workers may wish to request a colleague to accompany them during the interview to increase safety (NASW, 2013; Syracuse University School of Social Work, 2011).
        - A supervisor or colleague can serve as a safety buddy for their co-workers to ensure the safety of themselves and their partner while in the office (NASW, 2012).
    - When a worker is conducting a client meeting, their safety buddy may act as a look out for potential signs of an emergency and can call for help if needed (NASW, 2012).
    - All safety buddy partners should develop a clear plan of what to do in a harmful situation in the office and should have resources on hand at all times including proper phone numbers to call in an emergency and where to safely escape out of the office in an emergency (NASW, 2012).

- If a buddy system is not available, all colleagues and staff should be alerted before meeting with a client who may be dangerous (Newhill & Hagan, 2010).
- Offices used for client meetings should also contain safety technology including hidden panic buttons or internal alert systems to call for help to supervisors and law enforcement and should contain telephones programmed for 911 emergency calls (NASW, 2013; Newhill & Hagan, 2010; Taylor, 2011).
- Before meeting with clients, workers should familiarize themselves with the safety policies and procedures of the agency or organization with which they are employed including any safety plans, emergency exit paths, policies regarding harassment, and rights of employees (Nelson, n.d.; Syracuse University School of Social Work, 2011).
- Workers should review with their supervisors or colleagues the best ways to respond during specific emergency situations in the office according to their employer protocols and be familiar with where safety aids are stationed in the office and how to call for help or contact police (Nelson, n.d.; Syracuse University School of Social Work, 2011).
- When meeting with clients, workers should remain on high alert, strive to keep calm at all times, and always speak to their clients in a polite, respectful manner (Nelson, n.d.).
    - Before beginning an interview, workers should assess their client's mood, behaviors, and body language for indications of anxiousness, unrest, or anger (Quinn & Mason, n.d.).
    - If a worker is concerned about the mood and actions their client is displaying, they should inform their coworkers or supervisor, request a colleague to sit in on the interview, or keep the door ajar to allow others to provide assistance if needed (Quinn & Mason, n.d.).
- Workers should only conduct business and meet with clients during regularly scheduled business hours and must alert a co-worker if they will be staying in the office to work after hours (Syracuse University School of Social Work, 2011).
- Workers should always trust their instincts, pay attention to their feelings, and seek support when needed (Nelson, n.d.).

The following are office safety procedures enforced by the Massachusetts Department of Children and Families[2]:

- Agency reception rooms must contain fortified glass to protect workers.
- All employees must wear their ID badges when on duty, both in the office and in the field.

[2] The legislation for the Massachusetts Executive Office of Health and Human Services (EOHHS) was passed under Section 30 of House Bill 57 (HB57) as part of Massachusetts' 2013 fiscal year's supplemental budget. This law requires programs that provide direct services to clients that are operated by, licensed, certified, or funded by a department or division of the Massachusetts EOHHS have a workplace violence prevention and crisis response plan.

- All employees must follow the "violent client" protocol and are to schedule meetings with potentially violent or high risk clients on the day of the week when a police officer is present in the office.
- All office interview rooms must allow for a wide range of view.
- All office entrances must be equipped with coded entry.

(3) **B. Safety When Making Home Visits**

1. Plan for Visit

planning is key!

(3.1)

- In order to prepare for a client home visit, it is important to conduct a client risk assessment to review the client's past history of mental illness, violent incidents, or criminal behavior (Newhill & Hagan, 2010; NJDCF, 2015; Pope & Hadden, 2011; Taylor, 2011)[3].
- If client interviews can be made in the office, workers may wish to schedule an office visit as opposed to a home visit (Victor, 2014).
  - If a visit outside of the office is required, and the potential for danger exists, workers may work to schedule their client meeting in a public place (Victor, 2014).
- If a home visit is necessary, it is advised for workers to drive by their client's home before their first meeting takes place to assess the neighborhood and surroundings and analyze the safest place to park their car in order to reduce the risk for violence to occur (NASW, 2013; Nelson, n.d.; Pope & Hadden, 2011; Victor, 2014)[4].
  - Conducting an initial drive by and scan of the property allows for the worker to check certain risks that may exist around the client's home including uncontrolled animals, closed off entrances and exits, or loitering individuals (Pope & Hadden, 2011).
- In preparation for an unexpected event to occur, it is recommended for workers to design a safety plan detailing what to do in certain situations before embarking on a visit (Newhill 2012; Newhill & Hagan, 2010; Victor, 2014).
  - Conducting client risk assessments and formulating a plan of action helps workers to actively think of safety and lessen their fear of working with potentially violent clients (Quinn & Mason, n.d.).
  - When creating a safety plan, it may be helpful to examine previous experiences with similar types of client visits (Newhill, 2012).
- When visiting a high risk client, or a potentially violent client, it is crucial for workers to utilize a buddy system in order to be accompanied by a colleague during the visit (Newhill & Hagan, 2010; Victor, 2014)[5].
  - Workers may wish to conduct home visits in teams if possible, especially when conducting initial visits with unknown clients (NAIA, 2012).

[3] See Appendix 1: Risk Factors for Violent Behavior
[4] See Appendix 2: Worker Safety Intervention Plan
[5] See Appendix 3: Guidelines for Utilizing Teamed Response (Buddy System)

  - When a colleague is not available or the risk for violence is high, the worker should be escorted and accompanied on their visit by a police officer (Nelson, n.d.; Newhill & Hagan, 2010; Victor, 2014).
    - If a worker is unsure if a police officer should be contacted to request accompaniment on a client visit, they should consult with their supervisor (Pope & Hadden, 2011).
- Workers should also share their schedule with their coworkers or supervisor so others are aware of their whereabouts at all times (Newhill 2012; Newhill & Hagan, 2010; Victor, 2014).
  - Along with a schedule, workers should provide their supervisor or colleague with the address of where their client visit will take place, the reason for the visit, when they are expected to return, and information regarding the vehicle they will be taking on the visit (Cuadrado & Smith, n.d.; NAIA, 2012; NASW, 2013; Newhill, 2012; NJDCF, 2015; Pope & Hadden, 2011; Syracuse University School of Social Work, 2011).
  - Workers should also provide their supervisor or colleague with their vehicle information including their license plate number, and make, model, and color of the vehicle they are driving (Cuadrado & Smith, n.d.).
  - A sign-in/sign-out system may be helpful to track worker client visits and expected departure and arrival times (Pope & Hadden, 2011).
    - Notifying a supervisor or colleague of arrival and departure times helps to ensure someone will check-in and follow-up if a worker does not return when expected (Taylor, 2011).
  - Any changes in appointment field visits should be reported to the worker's supervisor or agency representative (NASW, 2013).
- Client home visits should always be made as early in the day as possible, when the sun is still out, and when supervisors and colleagues can be contacted in case of an emergency (Newhill & Hagan 2010; Quinn & Mason, n.d.).
- Workers should refrain from scheduling too many visits in one day (Newhill, 2012).
- Workers may wish to notify their client by phone that they will be visiting their home and advise them of the purpose of the visit, if possible (Nelson, n.d.; Syracuse University School of Social Work, 2011; Victor 2014).
  - Speaking to the client prior to the visit helps the worker retrieve important information regarding their client's situation, any safety precautions that may need to be taken according to the client, or whether or not their client's situation has changed (Nelson, n.d.; Victor, 2014).
  - If the interview is not an unannounced visit, clients should be advised as to what time the meeting will take place (Quinn & Mason, n.d.).
    - Additionally, clients should be notified by the worker when they are on their way to the client's home (Quinn & Mason, n.d.).
- Before leaving the office, workers should retrieve precise directions to their client's home and carry a guide or map in their car in case they become lost (NAIA, 2012; Syracuse University School of Social Work, 2011; Victor, 2014).

- It is also important for workers to have their cell phone with them, charged and ready to use (NAIA, 2012; NJDCF, 2015; Syracuse University School of Social Work, 2011; Victor, 2014).
  - All cell phones should be set to call 911 immediately in case of an emergency (Nelson, n.d.; Quinn & Mason, n.d.).
  - Agencies may wish to provide their workers with a cell phone equipped with a GPS tracker and audio or video recording features, and/or a personal safety device including a silent panic button or an identification badge holder with audio recording abilities (NASW, 2013).
- Before departing on a field visit, workers should ensure they are wearing appropriate clothes and shoes that allow for prolonged walking, standing, or climbing stairs and ample movement (Nelson, n.d.; Syracuse University School of Social Work, 2011; Taylor, 2011; Victor, 2014).
  - Workers should not wear accessories that can be easily pulled including a tie, necklace, or earrings and should pull back long hair (Nelson, n.d.; Taylor, 2011).
  - Additionally, workers should not wear expensive clothing items including watches, jewelry, or purses (NAIA, 2012; Quinn & Mason, n.d.).
  - Workers should refrain from carrying large amounts of cash on them when conducting home visits (NAIA, 2012).

4. Transport + travelling.

3.2

2. Traveling to Site

- When traveling to a client's home, workers must make sure their vehicle is in proper working condition (Newhill, 2012; NJDCF, 2015; Quinn & Mason, n.d.).
  - Workers should ensure their vehicle is serviced regularly (Taylor, 2011).
  - All vehicles should contain a full tank of gas, water, a functioning horn, a flashlight, first aid kit, and jumper cables (Nelson, n.d.).
  - If an agency car is being used, workers should take time to orient themselves with how to operate the car including how to turn on the high beams and emergency flashers (Newhill, 2012).
- Once a worker arrives at their client's home, they should park in an area that is well-lit and immediately lock their car doors upon exiting the vehicle (NAIA, 2012; Syracuse University School of Social Work, 2011).
  - It is suggested for workers to park their vehicle in spot that allows for easy access and departure and prevents the vehicle from being trapped or blocked in by other vehicles (Nelson, n.d.; Newhill, 2012; Newhill & Hagan, 2010; Taylor, 2011).
    - Workers may wish to park their car facing the direction they will be departing (NJDCF, 2015).
  - To ensure safety and respect for a client, workers should not park in the client's driveway or in front of their client's home (Newhill & Hagan, 2010; NJDCF, 2015; Syracuse University School of Social Work, 2011).
  - Special precaution should be taken when parking in a covered garage both when exiting and entering the garage (Nelson, n.d.).
- All personal items should be left in the car or trunk of the car and hidden from public eye sight (Cuadrado & Smith, n.d.; NJDCF, 2015).

  - Only items required to conduct the interview should be taken with the worker into the client's home (NJDCF, 2015).
- Before exiting the vehicle and approaching the client's home, workers should examine the neighborhood and their surroundings for skeptical activity (Newhill, 2012).
- Workers should be extra cautious and on high alert if visiting first time clients they have never met before, if the home is isolated or is in an area with a high crime rate, and when domestic violence is indicated (Newhill, 2012).
- If the situation feels unsafe, workers should remain in their vehicle and leave the premises (Cuadrado & Smith, n.d.).
- While walking up to the client's residence, workers should continue to scan the environment for possible dangers or risk factors and be actively listening for concerning or threatening noises throughout the neighborhood (Newhill, 2012; Newhill & Hagan, 2010; Syracuse University School of Social Work, 2011).
- It is suggested for workers to hide their money or valuable items from other's eye sight at all times and to walk as close to the sidewalk as possible (Taylor, 2011).
- Workers should refrain from providing those that are unfamiliar with their name, street address, and information regarding where they work. If they encounter anyone as they walk to their client's home, workers should keep normal, confident eye contact (Taylor, 2011).
- If a worker feels someone is following them, they should cross the street and go to the closest police station, residence, or open business (NAIA, 2012; Taylor, 2011).
  - Once workers arrive in a safe, public place, they are to immediately call the police (Taylor, 2011).
- If a worker is being threatened as they approach a home, they should shout as loud as possible for help (Taylor, 2011).
- Workers are advised to call their office before entering a home or to follow the protocols established by their agency before visiting clients in their home (Syracuse University School of Social Work, 2011).
- Before knocking on the client's door, workers should pay attention to hear if the parents are home and assess if it is safe for them to walk in to the home (NAIA, 2012; Quinn & Mason, n.d.).
  - Workers should listen critically for any indications of commotion occurring in the home (NJDCF, 2015).
- After knocking on the front door, workers should not stand in close proximity to the door and may wish to move off to the side when waiting for their clients to open the door (Nelson, n.d.; Victor, 2014).
- If going in to a client's home feels unsafe, workers should leave the residence promptly (NAIA, 2012).

3.3

3. During the Visit

- During the interview, workers are encouraged to keep their car keys and a functioning cell phone on them in a place that allows those items to be reached easily (Newhill, 2012; Newhill & Hagan, 2010; Pope & Hadden, 2011).

- When introducing themselves, workers should clearly state who they are, and why they are there (Newhill, 2012; Quinn & Mason, n.d.).
    - Workers should identify the reason for the visit and their need to meet with the client (Cuadrado & Smith, n.d.).
    - If the individual the worker is meant to meet with is not home, workers should leave the client's home (Quinn & Mason, n.d.).
- When entering the client's home, workers should enter through a door that is visible from the street (NJDCF, 2015).
- Workers must refrain from entering the home until they are welcomed to come inside (NJDCF, 2015).
- Once the worker is welcomed in to the home, it is important to scan the home for exits and to stay as close to the front door or exit as possible to allow for rapid departure if necessary (Nelson, n.d.; Newhill, 2012; Newhill & Hagan, 2010; Pope & Hadden, 2011; Taylor, 2011; Victor, 2014).
    - While scanning for exits, workers should identify if any exits in the home are blocked (NJDCF, 2015).
- Workers should determine where the meeting will take place in the home to avoid meeting in areas of the house that present dangers (Quinn & Mason, n.d.).
    - Meeting in the kitchen of a client's home presents dangers such as access to knives and boiling water or other kitchen utensils that can be used as weapons (Newhill & Hagan, 2010; Syracuse University School of Social Work, 2011; Taylor, 2011).
    - Workers should never meet in the client's kitchen if violence is a concern or if they do not know their client (Newhill, 2012).
- Workers should avoid helping themselves to a seat until they are invited by their client to sit down (Syracuse University School of Social Work, 2011).
    - It is advised for workers to position themselves in the middle of their client and the closest exit (NJDCF, 2015).
    - When choosing a place to sit, if possible, workers should sit in a chair with a straight back to allow for the ability to rise out of the chair in a rush if needed (Syracuse University School of Social Work, 2011).
    - Workers should be sure to sit in seat that is inviting and not confrontational (Taylor, 2011).
    - The safest position to sit is closest to an exit (NAIA, 2012).
        - If it is not possible to sit near a door or exit, workers should always keep their eye on the closest exit (Pope & Hadden, 2011; Syracuse University School of Social Work, 2011).
    - Additionally, it is important for workers to keep in mind when sitting down to begin an interview to keep an appropriate, safe distance in respect of the client's personal space (Syracuse University School of Social Work, 2011).
- Workers should refrain from going in to isolated, unlit rooms in the home and avoid entering basements of the home (NAIA, 2012).

    - Workers should not enter their client's bedrooms during the visit (Cuadrado & Smith, n.d.).
- Workers should scan the home for others present, and keep their eye on the front door if anyone enters the home (NAIA, 2012).
    - If anyone comes in to the home throughout the visit, the worker may obtain the names of those individuals by asking their client (Pope & Hadden, 2011).
    - If there are too many people in the home or if weapons or drugs are presented, workers should terminate the interview and exit the client's home (NAIA, 2012; Quinn & Mason, n.d.).
- Workers should avoid giving out personal information such as their home address or phone number to their clients (Syracuse University School of Social Work, 2011).
- If a client has pets in their home, workers are permitted to ask for the pets to be restrained while the visit takes place (Pope & Hadden, 2011).
    - Workers may request the animal be put outside or in another room (Syracuse University School of Social Work, 2011; Victor, 2014).
    - If a worker has allergies to domestic animals, precautions and arrangements should be made before the home visit takes place if a client has pets in their home (Victor, 2014).
    - Workers should also be cautious of clients opening their front door and allowing their dog to jump on them (Victor, 2014).
        - If a dog approaches a worker, they should remove their sunglasses, stand tall and refrain from moving, allow the dog to walk towards them, refrain from facing their back to the dog, speak using loud, firm commands, protect their neck and face, and try to give the dog something to chew or bite instead of their arm or hand (Cuadrado & Smith, n.d.).
- If the client has a television on during the visit, it is appropriate for workers to ask for the TV to be turned down or shut off due to the worker's trouble hearing (Syracuse University School of Social Work, 2011).
- It may be necessary for workers to connect their clients with information on how to get rid of or prevent bedbugs and refer them to a professional pest service (Victor, 2014).
    - It is common for bedbugs to hide in living room furniture and bedrooms causing potential risk for workers when visiting clients in a home (Victor, 2014).
- Before starting the interview, workers should asses their client's mood and adjust their mood to match their client's (Turner, 2015).
- While conducting the interview, it is helpful for the worker to keep in mind that while they may see the visit as a general concern and an approach to help, clients may view the visit as a threat (Newhill, 2012).
- Workers should assess their body language to ensure they are not promoting confrontational body language (NJDCF, 2015).
- Throughout the visit, workers should be on the lookout for dangers and trust their gut feelings (NAIA, 2012).

- It is best for workers to take a collaborative approach when conducting their interview and to give their clients an opportunity to let off steam or aggression before an emotion intensifies and a situation becomes violent (Newhill, 2012).
    - If a client becomes agitated, it is important for the worker to validate their feelings (Cuadrado & Smith, n.d.).
    - Workers should not force their clients to answer any questions (Cuadrado & Smith, n.d.).
- Clients should be treated with dignity and respect at all times (Newhill & Hagan, 2010).
- Clients should not feel cornered or be physically cornered by the worker (Cuadrado & Smith, n.d.).
- Workers should remain calm and collected throughout the visit and not show any signs of fear (NJDCF, 2015; Victor, 2014).
- It is important for workers to be on constant alert and be prepared to end the visit and leave the client's home if a situation becomes heated or escalated or the risk for violence is high (NJDCF, 2015; Pope & Hadden, 2011; Syracuse University School of Social Work, 2011).

4. Returning to the Office

- Once the visit has concluded, workers should prepare to approach their vehicle by having their keys ready to unlock their vehicle (Nelson, n.d.; NJDCF, 2015; Taylor, 2011).
- Prior to entering the car, workers should scan the floors and front and back seats of their vehicle (Nelson, n.d.; NJDCF, 2015; Syracuse University School of Social Work, 2011; Taylor, 2011).
- It is important for workers to be aware of their surroundings at all times. If workers know or perceive they are being followed, they should immediately drive to the closest police or fire station. If a police or fire station is not in a close distance, workers should drive to an open gas station or business and call the police when safe (Syracuse University School of Social Work, 2011).
    - Upon arrival at a safe place, workers should remain in their car, lock the doors, and sound the horn or flash their lights to attract attention (Taylor, 2011).
    - Workers should request to see personal identification of all personnel offering to help them (Nelson, n.d.).
- If a worker is approached by someone trying to force entry into their car, they should sound their horn and drive away as soon and as quickly as possible (Nelson, n.d.).
- When returning to their office, workers should not exit their vehicle unless they can walk without harm into the building (Syracuse University School of Social Work, 2011).
- Overall, workers must trust their instincts and never conduct an interview if they feel at risk (Newhill & Hagan, 2010).
- It is important to be aware of body language and to either call for additional help, or leave a situation immediately if a worker feels unsafe at any time (Nelson, n.d.; Syracuse University School of Social Work, 2011; Victor, 2014).
- Workers should refrain from letting their eagerness to help take precedence over caution (Newhill, 2012).

- If a client visit starts to escalate and the situation shows signs of impending violence, workers should leave right away (Newhill, 2012).
- If a worker gets caught in a heated situation and is not able to escape, the use of a verbal ploy, for example asking for a glass of water, may help to halt an escalated situation and allow the client or parties involved to cool off (Newhill, 2012).

The following are a few summaries regarding workplace safety initiatives utilized in the United States and results of a safety program piloted in Canada:

Massachusetts Department of Children and Families (DCF) Safety Initiatives

The Massachusetts Department of Children and Families (DCF) provides every staff with safety handbooks detailing their worker safety protocols and provides worker safety training to staff on what to do in a violent situation with a client (Sioco, 2010). The Massachusetts DCF instills a 'buddy system' in the event of an emergency which requires at least two workers to conduct the client visit together (Sioco, 2010). If a risk of violence is present, DCF will contact law enforcement and request a police escort to accompany a worker on their client visit. In addition to the buddy system, the Massachusetts DCF provides all social workers with a cell phone to allow for constant communication with their supervisors and the ability to call law enforcement in the case of an emergency (Sioco, 2010).

Child Welfare League of America (CWLA) Safety Initiatives

Members of the Child Welfare League of America (CWLA) in Miami, Florida have created a program titled 'OK Connect" to further ensure the safety of its workers in the field (Sioco, 2010). Through the OK Connect program, workers are provided with either a Samsung Blackjack cell phone or a Panasonic laptop. Each cell phone or laptop is connected to a GPS system online alerting management of their worker's whereabouts at all times (Sioco, 2010). In the event of an emergency situation, workers have access to an alarm by pressing a button on their phone or laptop which will send a notification to their supervisors and management that an emergency is occurring (Sioco, 2010). The Philadelphia Department of Human Services also piloted a program where 25 caseworkers working with children under the age of five will be equipped with a mobile device to be used in case of an emergency (Sioco, 2010).

Results of 2011 Piloted Safety Program

A study published in 2011analyzed the effects of a piloted staff safety program for employees providing social services in both institutional and community-based settings at the Western Health agency in Newfoundland, Canada. The safety program consisted of a Risk Assessment Screening Tool (WHRAST), a sign-in/sign-out system, a buddy system, and education and training sessions for staff. Staff were surveyed prior to and after the implementation of the safety program to determine if the program was effective in increasing staff safety during home visits with their clients. Information regarding client safety was gathered via surveys, focus groups, and key informant interviews.

Included in the risk assessment screening tool was safety protocols to be followed according to each noted risk (Hutchings, Lundrigan, Mathews, Lynch, & Goosney, 2011). For all situations identified as high risk, workers met with their supervisor to create a detailed safety plan.

Whether or not a client visit posed a risk, all staff were required to utilize the sign-in/sign-out system to monitor staff visits. Prior to embarking on a home visit, every worker completed a sign-out form noting their estimated time of return. Once completed, the form was given to the appropriate administrative support person. If an employee did not arrive back at the office when expected, the administrative support person contacted the manager who would then call the employee to confirm their safety. For all situations assessed as being high risk, the buddy system was enforced. While developing the safety plan, the worker's manager would provide the employee with a buddy to support the worker when making a home visit via a phone call or as a physical accompaniment with a colleague of the program, a family member of client present at time of the home visit, or escort by a law enforcement officer.

With responses from 42 pre-implementation surveys and 54 post-implementation, results showed the preferred elements of a staff safety program include a risk assessment tool, a buddy system for high-risk situations, and a sign-in/sign-out system for home visits. Respondents felt an increased awareness of safety due to use of the risk assessment tool, however, if there were many unknowns about a client, the assessment tool was not helpful. The post-implementation survey results found 72.7% of the respondents stated they were not always warned about a client's history of abuse or violence. Additionally, many staff mentioned appreciating having a colleague check-in with them if they did not return back to the office on time. With the sign-in/sign-out system came a sense of security knowing someone was tracking their whereabouts when on a home visit. Participants of the survey noted clerical support was not always available resulting in no support staff around to track employee's whereabouts, making the sign-in/sign-out system ineffective. Additionally, workers felt the buddy system was helpful, however, staff shortages often caused challenges preventing the buddy system to function as planned. Overall, most staff felt the safety program was successful in gaining safety awareness amongst staff.

– **C. Safety When Transporting Clients** (4) – no code specifically for this?

- When it is necessary to transport clients, workers must follow guidelines to keep both themselves and their client safe (NASW, 2013).
- Any worries or fear regarding transporting a client should be discussed with a supervisor before the trip occurs (Quinn & Mason, n.d.).
- Before a worker welcomes the client into the vehicle, the worker should conduct a client risk assessment to assess the client's current state of being (Quinn & Mason, n.d.).
- Workers should evaluate if the client is displaying signs of aggression, if the client is under the influence of drugs, and if the client is in possession of a weapon (NASW, 2013).
    - If a client shows signs of agitation or anger, workers should refrain from personally transporting the client (NASW, 2013; Quinn & Mason, n.d.).
    - If it is unsafe for the client to be transported by the worker, law enforcement should be called to transport the client (Quinn & Mason, n.d.).
- In addition to assessing for safety of the client, workers should also determine if the vehicle is safe to use (NASW, 2013).
    - Workers should check to see if there are any items in the car that could be used as a weapon and if so, should remove them immediately (NASW, 2013).

        - Items that could be used as a weapon include pens, pencils, books, or hot beverages (NASW, 2013).
    - Additionally, workers should determine if the car is in proper functioning order (NASW, 2013).
        - The vehicle should contain a full tank of gas, have working headlights and taillights, and should contain emergency safety equipment including jumper cables, road flares, and a spare tire (NASW, 2013).
- While the vehicle is in operation, clients should be seated behind the passenger seat of the car (Quinn & Mason, n.d.).
- Assessments of the client's state of being should be conducted on an on-going basis throughout the trip (Quinn & Mason, n.d.).
- Workers should drive in the far right hand lane of the road (Quinn & Mason, n.d.).
- When the vehicle is unoccupied, workers should keep the keys to the vehicle on them and ensure the doors are securely locked (Quinn & Mason, n.d).
- If a worker is transporting a young child, the worker should ensure all child safety locks are turned on in the car and must provide the child with an appropriate car seat according to the child's size and age (NASW, 2013).
- If a colleague is available to accompany the worker, a buddy system should be utilized to ensure safety when transporting clients (NASW, 2013).

(1) **D. Defusing Techniques**

(1.5)

While it is uncommon for clients to break out in violence without warning, it is important for workers to understand warning signs of potential violent situations before they occur including verbal threats, abusive or offensive language, and a client's previous history (Nelson, n.d.). If a worker finds themselves in a situation with a heated, unarmed client, and the potential for violence to occur, steps can be taken to de-escalate the situation. The primary goal when working to defuse a situation is to lessen the emotional anger and frustration felt by a client in order to reach the ability to hold a conversation (NASW-MA, 2011). In order to increase success while using verbal de-escalation techniques, workers must be in control of their emotions and minds, hold a strategic physical stance, and engage in a de-escalation discussion with the client (NASW-MA, 2011). Verbal de-escalation is an unnatural approach that defies the innate flight or fight response often triggered when frightened or in a state of panic (NASW-MA, 2011). Therefore, when employing de-escalation methods, it is critical for workers to come across as calm and collected although they may be in fear (NASW-MA, 2011).

1. Preparing for a De-escalation Discussion

- When preparing to hold a de-escalation discussion, workers first must work to disguise their feelings of stress with feelings of confidence, and relaxation (NASW-MA, 2011).
- Workers must remain calm when a client begins to become agitated or violent (Quinn & Mason, n.d.).
    - It may help workers to calm down by stopping to take a deep breath (Nelson, n.d.).
- Additionally, workers must keep in mind the importance of exemplifying self-confidence and not portraying themselves as a victim (Taylor, 2011).

- Workers should relax their facial expression to hide any signs of anxiety that may trigger the client to become further escalated and use a balanced, soft, low tone of voice when speaking with their client (NASW-MA, 2011; Nelson, n.d.; Newhill & Hagan, 2010; Syracuse University School of Social Work, 2011; Taylor, 2011).
- It is important for workers to prevent themselves from taking offense to any words or comments directed at them and to convey dignity and respect for their client at all times (Newhill & Hagan, 2010; Turner, 2015).
    - Agitated or hostile clients have the potential to become even more aggressive if they feel they are being judged and/or disrespected (Newhill & Hagan, 2010).

non-verbal.

2. Body Language and a Worker's Physical Stance

1.6

- Workers should evaluate the placement of surrounding unsecure objects that could be used as a weapon and position themselves between their client and those objects (Tuner, 2015).
- Additionally, workers must place themselves in way that does not leave their client cornered (Turner, 2015).
- When engaging in a conversation to defuse a situation, workers should be sure they are at a common eye level with their client (Elliot, 2012; NASW-MA, 2011; Taylor, 2011).
- It is suggested for workers to ask their client to take a seat (Taylor, 2011).
    - However, if the client wishes to stand, the worker must stand as well to remain at a balanced level of power (NASW-MA, 2011, Quinn & Mason, n.d.).
- During this time, workers must practice steady breathing, relax, and model nonaggressive body language for the client to follow (Turner, 2015).
- Workers should create an exaggerated distance between themselves and their clients, and stand at an angle as opposed to face on to their client while being sure to never turn their back on their client (NASW-MA, 2011; Syracuse University School of Social Work, 2011).
- Workers should not approach someone who is angry or scared until they have determined they are not the reason the person is in fear or until they are requested by their client to console them (Elliot, 2012).
- While working to defuse a situation, workers should not grin at their client, point or wave their finger at their client, or touch their client during this time (Taylor, 2011).
- It is important for workers to avoid sharing eye contact with their client for long periods of time and to look away to allow for their client to break their stare and glance elsewhere (NASW-MA, 2011; Turner, 2015).
- Additionally, workers should assess their client's focus of vision and take note of where they are looking. People experiencing frustration are likely to direct their visual attention towards what is causing them to feel angry (Elliot, 2012).
- Workers should keep their hands free from their pockets, and visible to allow for protection and to show the client the worker is not armed (NASW-MA, 2011).

3. Holding a Diffusing Conversation

- In order to hold a conversation to diffuse anger and aggression, workers must remember to keep a calm, steady voice, abstain from yelling or talking loudly over the client, and listen intently (NASW-MA, 2011; Syracuse University School of Social Work, 2011).
- If the name of the client is known, workers should refer to their client by name when speaking to them (Turner, 2015).
- It is critical for workers to move slowly and to remain in control of the conversation and situation (Syracuse University School of Social Work, 2011).
- Workers should refrain from verbally disputing with their client or trying to persuade their client in any way (NASW-MA, 2011; Syracuse University School of Social Work, 2011).
- Workers should maintain a supportive approach and must not try to defend themselves, or reflect judgement onto the client (NASW-MA, 2011; Quinn & Mason, n.d.).
- Workers should work to prevent themselves from challenging or accusing their clients (Elliot, 2012).
- Workers should project their tone of voice just below their client's and gradually lower their energy and tone as their client becomes more calm (Taylor, 2011; Turner, 2015).
- Workers should guide the conversation with the intent to reduce arousal in their client. One technique is to offer the client choices which result in positive solutions to help to suggest different, safer behaviors (NASW-MA, 2011).
- It is important for workers to focus on their client's emotions first, and aim to control the conversation (Elliot, 2012).
- When speaking, workers must be sure to use clear, succinct sentences and refrain from swearing or using foul language (Turner, 2015).
    - Workers should be firm when speaking and relay to their client their want to help (Quinn & Mason, n.d.).
- It may also be helpful to actively listen to the client's words, express empathy with the client's situation and feelings without empathizing with their behavior, and to ask them questions about their thoughts as opposed to their emotions (NASW-MA, 2011; Taylor, 2011).
- Workers should refrain from ignoring any questions asked by a client and try to reframe all responses to questions (Turner, 2015).
- It is also important for workers to verbally recognize their client's anger and allow time for their client to talk (Elliot, 2012; Quinn & Mason, n.d.; Taylor, 2011; Turner, 2015).
- In an effort to divert the client's attention, workers may wish to offer a piece of candy or gum to the client (Quinn & Mason, n.d.).
- Workers must remember to trust their instincts and either remove themselves from the situation, alert a colleague for help if possible, tell their client to depart, or call 911 if the situation continues to escalate (NASW-MA, 2011; Syracuse University School of Social Work, 2011; Quinn & Mason, n.d.).
    - If a worker is assaulted by a client, they should speak loud and verbally call for help (Quinn & Mason, n.d.).

- If a client begins to de-escalate and shows signs of calming down, workers should praise their client for their actions regardless of how small of a step toward a more safe direction was made (Turner, 2015).

Defusing situations requires a strategic plan of action making practice and planning critical to limit indecisiveness (Turner, 2015). While these techniques do not come firsthand, workers must continually practice defusing methods in order to be able to use them quickly to prevent an emergency situation with an angered client (NASW-MA, 2011).

E. Reporting Incidents and Employer Response

- To reduce risk and ensure safety for all staff and personnel, agencies should have data management and reporting systems in place to keep track of reports of threats, physical acts of violence, and any destruction of property (NASW, 2013; Newhill & Hagan, 2010).
- Investigations and incident reports should be made with the intention of discovering the cause of the incident (OSHA, 2015).
- While it is required to report all incidents involving death, injury, or illness, any close call incidents that may have resulted in those instances should also be investigated (OSHA, 2015).
- Proper and thorough investigations of workplace violence should be conducted to help prevent workplace injuries and deaths in the future (OSHA, 2015).
- When investigating an incident, police reports should be obtained and assessed to determine injuries or harm to certain departments or units; employee work spaces, job positions, employee tasks, or times of day (OSHA, 2015).
- When responding to incidents, employers should provide immediate aid and support to any injured employees and conduct the measures needed to prohibit any other individuals from being injured (OSHA, 2015).
- Responding employers should evaluate the situation to determine if professional medical assistance is needed, ensure injured staff are provided with immediate treatment, and provide all staff, clients, and witnesses involved in the situation a psychological evaluation and opportunity to debrief the situation (NASW, 2013; OSHA, 2015).
- As soon as the urgent needs of the situation are handled, an incident report should be conducted. Employers must determine the proper authorities to be notified both within the organization and outside of the organization (OSHA, 2015).
  - Incident reports should contain specifics of the situation including what occurred during the incident, where and when the event took place, a list of persons present, an explanation of any injuries, all factors leading up to the incident and a note stating whether or not medical personnel were called (NASW, 2013).
  - Workers present during the situation should be included in the investigation and incident report due to the critical information they may hold as to what may have caused the incident (OSHA, 2015).

  - It may also be necessary for management to collect additional information including training records, history of building repairs and inspections, audits, or past incident reports (OSHA,2015)
  - After the event occurs, management should meet to create a detailed plan of safety in reaction to the incident (NASW, 2013).
- Agencies should produce and manage an organizational culture that promotes safety and security for their staff (NASW, 2013).
  - To ensure an organizational climate of safety in every office, management should conduct regular safety discussions during both staff trainings and meetings (Newhill & Hagan, 2010).
  - Management should notify all employees of its commitment to promote the safety of all staff members both verbally and in writing (NASW, 2013).
- Social service agencies should support an environment of safety that promotes a proactive approach to prevention of violence in the workplace (NASW, 2013).
- Management should provide supportive post-incident resources for workers subject to workplace violence (OSHA, 2015)
  - Employee assistance programs are necessary to assist workers with the effects associated with experiencing workplace violence and to prepare them to resist or avoid potential harmful situations in the future (OSHA, 2015).
- All workers, including supervisors and management, should be encouraged to discuss safety concerns with their staff and supervisors should offer time for their employees to hold conversations regarding safety (Elliot, 2012).
- Agency supervisors or managers should collect, analyze, and input feedback from frontline staff regarding field and office safety when creating worker safety agency policies and procedures (Newhill & Hagan, 2010).
- Office safety checks should be conducted by management routinely (Newhill & Hagan, 2010).

The following are Violence Prevention and Crisis Response Plan Guidelines for human service workers enforced by the Massachusetts's Executive Office of Health and Human Services. These guidelines went in to effect on February 15, 2015:

- All programs must assess any previous workplace violence incidents and create a safety plan of action consistent with its situations, services offered, and personnel served.
- The development of safety committees are encouraged for all health and human service agencies with the purpose of preventing workplace violence, creating emergency incident response methods, and tracking completion of required employee worker safety trainings.
- All programs are to generate a report of their safety plans and provide them to any employees, if requested. All safety plans must be published for public view and easily accessible to workers.
- Every program is required to establish and support a workplace violence prevention and incident response plan that includes specific strategies to prevent incidents of workplace violence and protocols for responding to emergency situations.

- Every program must have established prevention of violence and emergency response plans.
- Violence prevention plans must include:
  - Safety strategies and protocols set in place to limit the risk of violence in the workplace.
    - Examples include modern security features in buildings, panic buttons, safety training, and policies regarding staffing and programs.
  - The requirement of all staff to complete eLearning or in-person safety trainings that meet the guidelines set by the Executive Office of Health and Human Services (EOHHS).
    - All new hires must complete safety training within the first three months of beginning employment.
    - All training completion must be tracked and kept on record.
    - Employees must take refresher safety trainings every two years.
  - Yearly workplace assessments to review incidents of workplace violence and what action was taken, and the effectiveness of building security systems in place.
- Violence prevention plans may include:
  - The ability to alarm others of an emergency or need for emergency assistance through the use of technology.
  - Assessment of agency staffing, security systems, and escape routes.
  - Workplace safety teams or committees to oversee that safety policies and procedures are adhered to.
- Every program must develop emergency response protocols for any occasion of workplace violence towards an employee.
- Emergency response protocols must include:
  - Set guidelines for reporting incidents of workplace violence available for all employees.
  - A system or database in place to track all incidents of workplace violence.
    - All reports must include names of the individuals involved, date, time and location of the incident, a description of the situation, and the extent of all injuries.
  - Assistance and allocation of resources for all individuals involved in an incident of workplace violence.
  - A policy stating any act of workplace violence is prohibited and is cause for discipline and end of employment.
  - A policy prohibiting the retaliation against individuals who report an incident.
- Emergency response plans may include:
  - Workplace safety teams or committees to support and offer help to employee subject to workplace violence.

1A

F. Establishing Committees

In order to keep employees and workplaces safe and supported, health and human service agencies are advised to develop safety policies and procedures involving the creation of safety teams or committees (NASW, 2013). Safety committees should be developed to continually analyze the effectiveness of current safety policies and procedures, make changes or add to existing policies, provide support for employees in the event that an incident occurs, and prepare

for safety before an emergency situation takes place (Elliot, 2012; NASW, 2013). Safety committees should keep record of all communication and decisions made during meetings to ensure all safety issues are addressed (NASW, 2012).

The Massachusetts Department of Children and Families have created a policy of forming safety committees both within agencies and amongst agencies in the state of Massachusetts (Sioco, 2010). The safety committees are in place to ensure the safety of its staff members and to notify all employees of possible dangers (Sioco, 2010). The safety committees are responsible for tracking all known and perceived threats to employees and keeping record of them in an incident reporting system (Sioco, 2010). All agency safety committees gather on a monthly basis (Sioco, 2010).

The Massachusetts National Association of Social Workers has also created a statewide safety committee. The statewide safety committee convenes quarterly to review and analyze the effectiveness of current worker safety policies and procedures (Sioco, 2010). Once a report of a threat is made, the safety committee works to create a safety plan for the worker prior to their meeting with a client (Sioco, 2010). All workers are encouraged to report all known or perceived threats.

1 1.5

G. Worker Safety Trainings

Client Violence and Social Worker Safety[6] is a 60 minute webinar training taught by Dr. Christina Newhill, PhD, ACSW, as part of the NASW Lunchtime Series Webinar. This training is available for all NASW members, free of charge. Included in this training is information regarding possible causes of client violence, the importance of assessing client risk factors for violent behavior, and what risk factors to assess for when evaluating clients.

Risk Assessment of Violent Clients and Social Worker Safety[7] is a webinar training provided by the National Association of Social Workers. This training, taught by Christina Newhill, PhD, ACSW, presents information on both the prevalence and types of workplace violence in health and human service fields, and offers tips on how to support a safe environment in the office and when conducting interviews in the field.

The *Social Worker Safety and Situational Awareness*[8] training is a 90 minute worker safety training recommended by the National Association of Social Workers which aims to provide personal safety tips for social service professionals when working both in the office and in the field. The training, which is offered in person or as a live webinar, covers topics including ways to improve awareness of one's environment, procedures when visiting high risk clients, trusting intuition, de-escalation techniques, safety when traveling on the job, and safety in elevators,

[6] Newhill, C. E. (2011). Client violence and social worker safety. NASW Lunchtime Series Webinar. Retrieved from http://c.ymcdn.com/sites/www.naswoh.org/resource/resmgr/imported/Client%20Violence%20and%20Social%20Worker%20Safety.pdf.

[7] Newhill, C. E. (2012). Risk assessment of violent clients and social worker safety. *University of Pittsburgh School of Social Work*. Retrieved from http://www.socialwork.pitt.edu/sites/default/files/Pdf_Files/Session%2012%20HANDOUT.pdf.

[8] The Personal Safety Training Group. (n.d.). Social worker safety and situational awareness training. Retrieved from http://www.personalsafetygroup.com/training/social-work-safety/.

parking lots and secluded areas. Additionally, the training promotes the need for sharing client interview schedules, determining personnel to contact in case of an emergency, designating check-in times, and communicating safety plans. This training is intended for social workers, Licensed Marriage and Family Therapists, and Licensed Mental Health Counselors.

The Federal Emergency Management Agency (FEMA) released an online active shooter training titled *Active Shooter: What You Can Do*[9] to prepare all employees for potential active shooter situations. Training participants are guided on how to take action when in an active shooter situation, how to identify clues of potential violence in the workplace, ways to prevent violent incidents, and how to control the effects of an active shooter situation (FEMA, 2013).

Home Visiting with Families Affected by Substance Abuse and/or HIV[10] is a 57 minute online tutorial provided by the National Abandoned Infants Assistance Resource Center at the University of California, Berkeley. This training discusses the main procedures associated with home visits with a focus on how to remain safe when conducting client visits in the home.

The *Everyday Self Defense for Social Workers*[11] training was created in Kansas by Janet Nelson, ACSW, LCSW. The state of Kansas mandates all licensed social workers to take self-defense training. The self-defense training combines personal safety awareness skills with conflict avoidance skills, positive communication, stress reduction, and practice with physical skills. In addition, trainees learn techniques to using words, body language, and body positioning in order to avert and escape dangerous situations. The training helps participants increase their alertness, balance, and control. The self-defense training is taught both in person and online. The class qualifies for the safety awareness CE for both Kansas state social workers and social workers in other states, and has received authorization by the National Association of Social Workers.

The California Social Work Education Center (CALSWEC) has established competencies and learning objectives regarding *Introduction to Social Worker Safety in a Child Welfare Context*[12]. The worker safety training learning objectives, based on California Common Core curricula for child welfare workers, are divided into knowledge, skills, and values. After participation in the training, social workers should possess the knowledge to identify potential signs of harm, determine means of escaping danger, list factors that may cause a crisis, identify when and how to end an interview safely in an emergency, and utilize de-escalation techniques (CALSWEC, 2012). Through case examples and role play, trainees will develop skills to prepare for client home visits and will possess knowledge of various techniques to de-escalate heated situations with clients. Additionally, the training introduces values of managing personal safety through the use of commutation skills, both verbally and nonverbally, observing and reacting to possible

[9] Federal Emergency Management Agency. (2013). Active shooter: What you can do. Retrieved from https://training.fema.gov/is/courseoverview.aspx?code=IS-907.
[10] National AIA Resource Center. (2012). Home Visiting with Families Affected by Substance Abuse and/or HIV. *U.C. Berkeley*. Retrieved from http://aia.berkeley.edu/training/online/tutorials/.
[11] Nelson, J. (n.d.). Everyday self-defense for social workers. Retrieved from http://www.everydayselfdefense.com/online-classes.html.
[12] California Social Work Education Center. (2012). Introduction to social worker safety in a child welfare context. Retrieved from http://calswec.berkeley.edu/files/uploads/lo_swsafety_v2.0_final.pdf.

signs of danger, and the acknowledgement of cultural varieties when conducting client assessments (CALSWEC, 2012)[13].

The New Jersey Child Welfare Training Partnership in association with the New Jersey Department of Children and Families held a worker safety training in 2015 titled *Safety Awareness for the Child Welfare Professional*[14]. This two-day training presented information through the use of lectures and activities regarding techniques to maintain safety when working with children and families. Topics of the training include the 4 A's of safety, warning signs of potentially unsafe situations, de-escalation techniques, and strategies to remain safe and promote both positive well-being and self-care.

The Connecticut Department of Social Services provides a training entitled *Worker Safety*[15] taught by Orlando Cuadrado, MSW and Michael Smith. This training provides information regarding identifying unsafe conditions, skills for developing awareness of self, the environment, and of clients, and how to manage in crisis situations.

Personal Safety in Clinical Practice[16] is taught by Phil Quinn, Ph.D., Director of South Shore Mental Health (SSMH), EAP Program, and Ray Mason, Director of SSMH, Metro-Suburban Outreach. This training provides participants with tips on how to exercise caution and maintain safety when interviewing clients in the office or in the client's home. The information provided includes tips for reviewing client records, conducting evaluations and observations prior to meeting with a client, traveling to a client's home, and what to do when a client becomes aggressive.

The Idaho Department of Health and Welfare Family and Community Services conducted a *Home Visitor Safety*[17] training in collaboration with their Social Worker Academy. This training discusses safety tips when preparing to conduct client home visits, when traveling to a client's home, and when conducting an interview in a client's home.

Run, Hide, Fight[18] is an online video produced by the Department of Homeland Security which asserts what to do in the case of an active shooter situation. After viewing this video, workers will learn to first make an effort to escape or exit the premises safely, hide if it is not possible to leave the area, or fight the individual if the worker's life is in jeopardy and the potential for harm

[13] See Appendix 4: Social Worker Safety Competencies and Learning Objectives in a Child Welfare Context
[14] New Jersey Department of Children and Families. (2015). Safety awareness for the child welfare professional. *New Jersey Child Welfare Training Partnership*. Retrieved from file:///C:/Users/Academy/Downloads/Safety%20Awareness%20-PARTICIPANT%20GUIDE%20Rev%205%2013%2015%20Includes%20Appendices[2]%20(1).pdf.
[15] Cuadrado, O., & Smith, M. (n.d.) Worker safety. Retrieved from http://ncwwi.org/files/Incentives__Work_Conditions/CT_DSS_WorkerSafetyTraining.pdf.
[16] Quinn, P., & Mason, R. (n.d.). Personal safety in clinical practice. Retrieved from https://c.ymcdn.com/sites/www.naswma.org/resource/resmgr/imported/Safety_SouthShoreMentalHealthSafetyTraining-4.pdf.
[17] Idaho Department of Health and Welfare Family and Community Services. (n.d.) Home visitor safety. *Social Worker Academy*. Retrieved from http://healthandwelfare.idaho.gov/Portals/0/Children/HomeVisiting/Home%20Visitor%20Safety%20Training.pdf.
[18] Department of Homeland Security. (2012). Run, hide, fight. Retrieved from https://www.youtube.com/watch?v=5VcSwejU2D0.

is high (DHS, 2012).

1.5

Guidelines for Establishing Worker Safety Trainings

- All agencies should provide worker safety training to all staff and new hires and provide workers with risk management training when conducting interviews in the home and in the office (Newhill & Hagan, 2010).
  - All worker safety trainings should describe current agency policies and procedures in place in addition to de-escalation and self-defense skills (OSHA, 2015).
  - Worker safety trainings should be offered annually in order to refresh and improve on worker's safety practices (NASW, 2013).
  - Worker Safety trainings may involve:
    - Prevention of workplace violence (OSHA, 2015)
    - Techniques to determine, avoid or diffuse violent situations or dangerous behavior (OSHA, 2015)
    - Possible risk factors that may lead to harmful situations (OSHA, 2015)
    - Protocols for reporting varying client behavior (OSHA, 2015)
    - Use of safety devices including panic buttons and alarm systems (NASW, 2013; OSHA, 2015)
    - Warning signals of potentially dangerous situations (OSHA, 2015)
    - Effective use of safe rooms or employee shelter areas (OSHA, 2015)
    - Emergency response plan procedures (OSHA, 2015)
    - Use of a buddy system (OSHA, 2015)
    - Proper procedures for reporting incidents (OSHA, 2015)
    - Protocols for staff assistance programs and agency incident response
    - Risk management and reduction (NASW, 2013)
    - Emergency evacuation procedures (NASW, 2013)
    - Defusing techniques (NASW, 2013)
    - Effective strategies for clinical interventions with violent or potentially violent clients (NASW, 2013)
    - Nonviolent self-defense (NASW, 2013)
    - The impact of and how to manage secondary trauma (NASW, 2013)
- All supervisors and management should be trained on determining potentially dangerous situations to assist in creating a safe workplace and to ensure the safety of their employees (OSHA, 2015).
  - Management must receive training which notes the importance of employees reporting incidents and obtaining necessary care after a harmful or violent situation occurs (OSHA, 2015).
  - All supervisors and managers should be provided with skills to reduce and prevent safety hazards in the workplace and should enforce that all workers take the appropriate and required worker safety trainings for their agency (OSHA, 2015).
- All trainings provided to employees should be reviewed and analyzed at least once a year (OSHA, 2015).

  - Training evaluations should include a review of the curriculum, the means of delivery, and an assessment of how often the training is provided (OSHA, 2015).
- All social work students should be taught best-practice safety procedures in the workplace during their student years before they enter the field (NASW, 2013).
  - All practicing social work students should be supervised in safe environments when working in the field (NASW, 2013).

H. Action by the Academy

? Unclear data.

The Academy for Professional Excellence[19], a project of San Diego State University School of Social Work, was established in 1996 to support the health and human service community by providing training, technical assistance, organizational development, research, and evaluation. Serving over 10,000 people annually, the Academy continues to grow with new programs and a diversity of training focused on serving the health and human services community in Southern California and beyond.

The Academy provides basic worker safety training through multiple programs[20]. The Public Child Welfare Training Academy (PCWTA) offers Lineworker Core training. The Behavioral Health Education and Training Academy (BHETA) provides training on violent or criminal behavior risk factors, and de-escalating violent situations. The Multi-disciplinary Adult Services Training and Evaluation for Results (MASTER) program offers an online worker safety course as part of their new worker core for Adult Protective Services workers. Additionally, the Academy has established a cross-program committee and is in the process of developing an advanced multi-disciplinary worker safety training. The worker safety training involves the development of an eLearning, simulation site training, and a mobile app in alignment with the eLearning. This training is intended for health and human service workers including behavioral health service providers, adult protective workers, and child welfare workers.

PCWTA offers a Lineworker Core training series for new child welfare workers based on the competencies developed by the California Social Work Education Center (CalSWEC). PCWTA's Core Line Worker Training is facilitated over a period of several weeks via half or multi-day trainings, eLearnings and field activities and is available across Southern California counties and throughout California via other Regional Training Academies. The classes in this course are taught by various experts in the field of child welfare services. Included in the Lineworker Core Training is *Self-Care for New Child Welfare Workers: Time Management, Stress Management and Worker Safety*. The topics of this training include signs of danger and methods of avoiding or mitigating danger while working as a child welfare worker, dynamics of a crises and effective crises intervention techniques used to assist families in crises, and effective communication skills that can be used to defuse or prevent violence, including when to terminate an interview due to safety concerns and how to leave a dangerous situation swiftly. Line Worker Core Training is in the process of a statewide curriculum revision entitled Common Core 3.0. All

[19] Academy for Professional Excellence. (n.d.). About the Academy. Retrieved from http://theacademy.sdsu.edu/about-the-academy/.
[20] The courses described in this section are Academy trainings that are offered under contract to child welfare, behavioral health, and adult protective service workers via a Learning Management System.

Line Worker Core trainings will encompass parts of the updated curriculum as of January 1, 2016.

The training *8 Major Criminogenic Risk Factors for Violent and Criminal Behaviors*[21] is an eLearning provided by the Behavioral Health Education & Training Academy within the Academy for Professional Excellence. This eLearning course provides information on risk factors for violent and criminal behaviors, discusses prevention and intervention techniques, and describes treatment methods to reduce risk.

Introduction to Working with People Who Are at Risk for Violent Behavior or Who Are Criminally Involved[22] is a recorded webinar provided by the Behavioral Health Education & Training Academy within the Academy for Professional Excellence. This eLearning is an introductory course on working with people who are at risk for violent behavior. After completing the training, participants will be able to identify the causes behind criminal and violent behavior, determine potential violent situations, and identify trauma-informed care techniques to ensure safety in both the office and in the field.

APS Worker Safety[23] is an eLearning provided by the MASTER program, Multi-disciplinary Adult Services Training and Evaluation for Results, within the Academy for Professional Excellence. This training teaches safety guidelines to follow when conducting home visits.

[21] Behavioral Health Education and Training Academy. (2014). 8 Major criminogenic risk factors for violent and criminal behaviors. *Academy for Professional Excellence.*
[22] Behavioral Health Education and Training Academy. (2014). Introduction to working with people who are at risk for violent behavior or who are criminally involved. *Academy for Professional Excellence.*
[23] Multi-disciplinary Adult Services Training and Evaluation for Results. (2014). APS worker safety. *Academy for Professional Excellence.*

References

Academy for Professional Excellence. (n.d.). About the Academy. Retrieved from http://theacademy.sdsu.edu/about-the-academy/.

Behavioral Health Education and Training Academy. (2014). Introduction to working with people who are at risk for violent behavior or who are criminally involved. *Academy for Professional Excellence.*

Behavioral Health Education and Training Academy. (2014). 8 Major criminogenic risk factors for violent and criminal behaviors. *Academy for Professional Excellence.*

California Social Work Education Center. (2012). Introduction to social worker safety in a child welfare context. Retrieved from http://calswec.berkeley.edu/files/uploads/lo_swsafety_v2.0_final.pdf.

Cuadrado, O., & Smith, M. (n.d.) Worker safety. Retrieved from http://ncwwi.org/files/Incentives__Work_Conditions/CT_DSS_WorkerSafetyTraining.pdf.

Department of Homeland Security. (2012). Run, hide, fight. Retrieved from https://www.youtube.com/watch?v=5VcSwejU2D0.

Elliot, D. (2012). Worker Safety for the APS Supervisor. *National Adult Protective Services Association.* Retrieved from http://www.napsa-now.org/wp-content/uploads/2012/06/Safety-APS-webinar.pdf.

Executive Office of Health and Human Services. (2013). Human service workers violence prevention and crisis response plan guidelines. Mass.gov. Retrieved from http://www.mass.gov/eohhs/gov/commissions-and-initiatives/hsw-safety-regs/guidelines-for-implementation.html.

Federal Emergency Management Agency. (2013). Active shooter: What you can do. Retrieved from https://training.fema.gov/is/courseoverview.aspx?code=IS-907.

HaeJung, K. (2012). Public child welfare workers' safety experiences: Predictors and impact on job withdrawal using mixed-methods approach. *University of Maryland, School of Social Work.* Retrieved from https://kb.osu.edu/dspace/bitstream/handle/1811/54751/NSDRSW_25_1_kim_paper.pdf?sequence=2.

Hutchings, D., Lundrigan, E., Mathews, M., Lynch, A., & Goosney, J. (2011). Keeping community health care workers safe. *Home Health Care Management & Practice*, 23(1), 27-35. doi:http://dx.doi.org/10.1177/1084822309360383.

Idaho Department of Health and Welfare Family and Community Services. (n.d.) Home visitor safety. *Social Worker Academy.* Retrieved from

http://healthandwelfare.idaho.gov/Portals/0/Children/HomeVisiting/Home%20Visitor%20Safety%20Training.pdf.

Kelly, J. J. (2010). The urgency of social worker safety. *National Association of Social Workers*. Retrieved from http://www.socialworkers.org/pubs/news/2010/10/social-worker-safety.asp.

Kim, H., & Hopkins, K. M. (2015). Child welfare workers' personal safety concerns and organizational commitment: The moderating role of social support. Human Service Organizations: Management, Leadership & Governance, 39(2), 101-115.

Multi-disciplinary Adult Services Training and Evaluation for Results. (2014). APS worker safety. *Academy for Professional Excellence*.

NASW. (2012). Organizing for office safety. *NASW Center for Workforce Studies*. Retrieved from http://careers.socialworkers.org/documents/Organizing%20for%20Safety.pdf.

NASW. (2013). Guidelines for social worker safety in the workplace. Retrieved from https://www.socialworkers.org/practice/naswstandards/safetystandards2013.pdf.

National AIA Resource Center. (2012). Home Visiting with Families Affected by Substance Abuse and/or HIV. *U.C. Berkeley*. Retrieved from http://aia.berkeley.edu/training/online/tutorials/.

NASW Massachusetts. (2011). Verbal de-escalation techniques. Retrieved from http://www.naswma.org/?page=520.

Nelson, J. (n.d.). Essential ideas for personal safety. *NASW North Carolina*. Retrieved from http://c.ymcdn.com/sites/www.naswnc.org/resource/resmgr/imported/Everyday%20Self%20Defense%20Tips%20Handout.pdf.

Nelson, J. (n.d.). Everyday self-defense for social workers. Retrieved from http://www.everydayselfdefense.com/online-classes.html.

Newhill, C. E. (2011). Client violence and social worker safety. NASW Lunchtime Series Webinar. Retrieved from http://c.ymcdn.com/sites/www.naswoh.org/resource/resmgr/imported/Client%20Violence%20and%20Social%20Worker%20Safety.pdf.

Newhill, C. E. (2012). Risk assessment of violent clients and social worker safety. *University of Pittsburgh School of Social Work*. Retrieved from http://www.socialwork.pitt.edu/sites/default/files/Pdf_Files/Session%2012%20HANDOUT.pdf.

Newhill, C. E., & Hagan, L. P. (2010). Violence in social work practice. *NASW Mental Health Section Connection*. Retrieved from http://www.socialworkers.org/assets/secured/documents/sections/private_practice/newsletters/2013%20Private%20Practice%20Newsletter%20-%20Summer%20Issue.pdf.

New Jersey Department of Children and Families. (2015). Safety Awareness for the child welfare professional. *New Jersey Child Welfare Training Partnership*. Retrieved from file:///C:/Users/Academy/Downloads/Safety%20Awareness%20-PARTICIPANT%20GUIDE%20Rev%205%2013%2015%20Includes%20Appendices[2]%20(1).pdf.

Occupational Safety and Health Administration. (2015). Guidelines for preventing workplace violence for healthcare and social service workers. *United States Department of Labor*. Retrieved from https://www.osha.gov/Publications/osha3148.pdf.

Occupational Safety & Health Administration. (2015). Safety and health topics. *United States Department of Labor*. Retrieved from https://www.osha.gov/SLTC/workplaceviolence/.

Pope, N. D., & Hadden, J. B. (2011). Tips for making home visits in child welfare. *The New Social Worker*, 18(3), 12-13. Retrieved from http://www.socialworker.com/featurearticles/practice/Tips_for_Making_Home_Visits_in_Child_Welfare/.

Quinn, P., & Mason, R. (n.d.). Personal safety in clinical practice. Retrieved from https://c.ymcdn.com/sites/www.naswma.org/resource/resmgr/imported/Safety_SouthShoreMentalHealthSafetyTraining-4.pdf.

Saturno, S. (2011). Violent crime and social worker safety. *Social Work Today*. Retrieved from http://www.socialworktoday.com/archive/exc_032511.shtml.

Sioco, M. C. (2010). Safety on the job: How managers can help workers. *Children's Voice*. Retrieved from https://mariacarmelasioco.carbonmade.com/projects/3259343#1.

Syracuse University School of Social Work. (2011). Social worker safety tips. Retrieved from http://falk.syr.edu/socialWork/documents/2011/BSW/Safety Tips 2011.pdf.

Taylor, B. J. (2011). Avoiding assault and defusing aggression. Transforming social work practice. *Southernhay East Exeter: Learning Matters*.

The Personal Safety Training Group. (n.d.). Social worker safety and situational awareness training. Retrieved from http://www.personalsafetygroup.com/training/social-work-safety/.

Turner, J. T. (2015). Working with people who are at risk for violent or criminal behavior. *Alliant International University*. Retrieved from file:///C:/Users/Academy/Downloads/People%20at%20Risk%20PPT%20slides.pdf.

Victor, P. (2014) 20 Safety tips for home visits. *NASW New York City Chapter*. Retrieved from http://www.naswnyc.org/?489.

Appendices

Appendix 1: Risk Factors for Violent Behavior[24]

- Demographic Risk Factors
  - Young Age
  - Male Gender
- Clinical Risk Factors
  - High Risk Psychiatric Symptoms (delusions, hallucinations, violent fantasies)
  - Personality Features (anger, emotion dysregulation, impulsivity)
  - Personality Disorder (antisocial, borderline)
  - Substance Abuse (especially alcohol)
- Biological Risk Factors
  - Low Intelligence Quotient (IQ)
  - Neurological Impairment
- Historical Risk Factors
  - History of violence (recency and frequency of self reports of violence toward others, arrests, incarcerations, and reports of violence toward self)
  - Social and family history (early exposure to violence);
    - Experiencing severe abuse by a parent or other caretaker or being a witness to domestic violence;
    - Being severely neglected or rejected by parent/caretaker;
    - Parental psychiatric illness and/or drug or alcohol abuse;
    - Tacit parental approval of cruelty toward other people or animals.
  - Work history (economic instability, unemployment);
  - History of psychiatric treatment and/or hospitalization, especially if involuntary;
- Environmental/ Contextual Risk Factors
  - Level and quality of social support
  - Peer pressure from peers who endorse violence
  - Influence of popular culture
  - Means for violence
  - Accessibility of the potential victim

① Awareness. + info. (1.1)

[24] Sources: Newhill, C. E. (2011). Client violence and social worker safety. NASW Lunchtime Series Webinar. Retrieved from http://c.ymcdn.com/sites/www.naswoh.org/resource/resmgr/imported/Client%20Violence%20and%20Social%20Worker%20Safety.pdf.
Newhill, C. E. (2012). Risk assessment of violent clients and social worker safety. *University of Pittsburgh School of Social Work*. Retrieved from http://www.socialwork.pitt.edu/sites/default/files/Pdf_Files/Session%2012%20HANDOUT.pdf.

Appendix 2: Worker Safety Intervention Plan[25]

"A worker safety intervention plan is formalized by the assigned worker and supervisor during case conferencing when a particular family member—an adult or a child—or family or environment has been identified as being potentially dangerous to staff or presenting a risk to the personal safety of staff and/or authority figures. A worker safety intervention plan can be a simple written outline or paragraph describing specific precautions to be taken upon intervention. Approval by the Casework Supervisor may be necessary, depending on the nature of the plan. Copies of the plan are kept by the worker and supervisor, and a copy is attached to the Resource Sheet filed in the case record."

Worker safety concerns are also documented in NJ SPIRIT applications, including the electronic NJS Worker Safety Alert currently being tested. "If a WORKER ALERT label is on the record, the worker must refer to the dated contact notes. REFER TO THAT PLAN BEFORE INITIATING CONTACT WITH THE FAMILY. The plan is dated and reviewed periodically during case conferencing and whenever the WORKER ALERT label is reviewed."

According to policy, worker safety intervention plans could include, but are not limited to, the following:

- Where and when it is best to meet with the family member (e.g., meet with family member at a neutral setting, not at the family home);
- Who, if anyone, should be present when CP&P field staff meet with the family member (e.g., another CP&P staff member should be present during home visits; the Human Services Police should accompany the assigned worker when new allegations are under investigation; the person's therapist may be willing to provide assistance/prevent a crisis);
- Whether or not the child should be present when CP&P meets with the parent(s); and
- Specific actions to be taken under certain circumstances which have been in evidence in the past (e.g., immediately leave the family home—with or without the children—if the person appears to be under the influence of drugs or alcohol or actively psychotic).

"It may be necessary to seek relief from the Family Court to ensure the worker's safety. Such an option should be discussed with the DAG. The worker safety intervention plan could specify 5 that relief from court will be pursued if a specific behavior persists or in the event of a new incident."

"If an after-hours crisis is anticipated, the assigned worker or supervisor contacts SCR and leaves special instructions for case handling precautions. If a case presents ongoing serious concerns

[25] Source: New Jersey Department of Children and Families. (2015). Safety awareness for the child welfare professional. *New Jersey Child Welfare Training Partnership*. Retrieved from file:///C:/Users/Academy/Downloads/Safety%20Awareness%20-PARTICIPANT%20GUIDE%20Rev%205%2013%2015%20Includes%20Appendices[2]%20(1).pdf.

regarding risk to staff safety upon intervention, the supervisor advises the County SPRU Coordinator, who forewarns the SPRU staff of risks associated with the case. In addition, the LO Manager consults with the SCR Administrator/designee to determine whether special arrangements are to be implemented to accommodate the case. Special arrangements may include requesting after hours assistance from the Human Service Police, in accordance with the Protocol for Requesting HSP Services, found in (CP&P-II-C- 4-300)."

Appendix 3: Guidelines for Utilizing Teamed Response (Buddy System)[26]

(1) (1.2)

"Absent compelling reason to do otherwise, deference should be given to the expressed safety concerns of the requesting worker. Supervisors shall not, under any circumstances, unreasonably deny or discourage the use of teamed field response as a means of ensuring worker safety."

Supervisors are required to approve a teamed response in the following circumstances:

1) Division records indicate a history involving:

a) assaults or threats of violence; or

b) a conviction involving the use of a weapon.
In these situations, the buddy should be the Human Services Police or other law enforcement officials.

2) All active cases that involve ongoing domestic violence situations where the alleged batterer resides in or frequents the home. For additional safety precautions, see the Domestic Violence Protocol.

3) All initial responses to allegations of abuse in unknown (no prior CP&P history) cases if requested by the assigned worker.

4) All responses to known high crime and initial responses to known drug-use locations.

a) High crime and drug-use areas are established by mutual agreement between Local Office Management and field staff on an office-by-office basis.

b) Areas designated as high crime or drug-use areas must be communicated by Local Office Management to the Area Director as soon as such determination is made.

c) Area Office SPRU Coordinators share this information with SPRU Supervisors and SPRU Workers.
The decision whether or not a SPRU Worker needs a teamed response is a decision that will be made by the Local Office or Area Director.

5) All out-of-home placements into a resource family home to help the child by lessening trauma associated with separation from the family of origin. The buddy permits the assigned worker to focus solely on the child's needs.

6) All cases where transporting a child with known behavioral problems (e.g., a history of sexual acting out, making false accusations, or running away). When transporting a group of children, a teamed response is necessary in order to assist with supervision of the children while driving to the destination. Requests for assistance by Human Services Police may be appropriate.

[26] Source: New Jersey Department of Children and Families. (2015). Safety Awareness for the child welfare professional. *New Jersey Child Welfare Training Partnership*. Retrieved from file:///C:/Users/Academy/Downloads/Safety%20Awareness%20-PARTICIPANT%20GUIDE%20Rev%205%2013%2015%20Includes%20Appendices[2]%20(1).pdf.

7) Previously assaulted field staff are entitled to a buddy until such time as the worker and his or her supervisor jointly decide that a buddy is no longer routinely needed. The input of a crisis counselor or a treating professional may be sought, if agreed to by the worker, to resolve questions or dispute about the issue of the worker's readiness to respond to routine Division field assignments alone.

There may be other circumstances not spelled out in this policy or not REQUIRED by policy. However, supervisors may determine it is necessary anyway based on the full circumstances. **If that happens, workers are NOT permitted to decline.**

The makeup of a team can vary, and a diverse group of professionals are listed in the policy section, including another CP&P staff member; a supervisor; FPS or YAP workers; the homemaker; school staff; or others.

In any circumstance where policy or prudence calls for a joint law enforcement response, law enforcement shall be used in lieu of any other type of buddy.

Appendix 4: Social Worker Safety Competencies and Learning Objectives in a Child Welfare Context

CALIFORNIA COMMON CORE CURRICULA

FOR CHILD WELFARE WORKERS

INTRODUCTION TO SOCIAL WORKER SAFETY IN A CHILD WELFARE CONTEXT

COMPETENCIES and LEARNING OBJECTIVES

RELEVANT CHILD WELFARE OUTCOMES

Well-being 1
Families have enhanced capacity to provide for their children's needs

Well-being 3
Children receive services adequate to their physical, emotional, and mental health needs

LEARNING OBJECTIVES

Knowledge:

K1. The trainee will be able to identify at least three possible signs of danger, taking signals from the family, the environment, and from themselves, while working as a child welfare social worker.

K2. The trainee will be able to identify at least three methods of avoiding or mitigating danger as a means of maintaining personal safety and increasing capacity to focus on the family.

K3. The trainee will be able to describe common family dynamics that can contribute to a crisis.

K4. The trainee will be able to explain when and how to terminate an interview due to safety concerns.

K5. The trainee will be able to describe effective communication skills that can be used to defuse or successfully prevent violent outbursts, including:

a. Acknowledging the power differential that exists;
b. Using neutral language and avoiding passing judgment;
c. Expressing empathy with family members' situations and feelings;
d. Describing family members in ways that let them know they are valued partners.

K6. The trainee will be able to describe the role of the supervisor in maintaining social worker safety, including:

a. Consulting with the social worker about his or her safety;

b. Providing information about county agency policies concerning child welfare social worker safety.

K7. The trainee will be able to describe how to leave a dangerous situation swiftly.

K8. The trainee will be able to recognize the difference between 'awareness' and 'hyper-vigilance.'

Skills:

S1. Given a case example or scenario, the trainee will be able to demonstrate the steps necessary for preparation for a home visit with personal safety in mind, including determining risk factors, being well informed about case information, and knowing the geographic area.

S2. Given a case scenario or role play, the trainee will be able to demonstrate communication skills for identifying and defusing potentially dangerous situations. (Optional: Depends on length of training day/module.)

Values:

V1. The trainee will value maintaining his or her own safety and seeking to avoid knowingly putting him- or herself in danger.

V2. The trainee will value the use of verbal and nonverbal communication skills which reduce the risk of hostility and increase child welfare social worker safety.

V3. The trainee will value recognizing and acknowledging internal reactions as a possible signal that a threat is present.

V4. The trainee will value consideration of cultural differences when making assessments related to social worker safety.

RELATED TITLE IV-E CURRICULUM COMPETENCIES

The Title IV-E MSW competencies were developed for the M.S.W. specialization in public child welfare in California, a two-year full time graduate program. The MSW competencies may overlap with the common core competencies, but are primarily designed for a full MSW program. Learning objectives and competencies in the common core support the MSW Title IV-E competencies, but not all of the IV-E material can be delivered during an in-service training session. MSW Title IV-E competencies may therefore be linked to multiple topic areas of the common core.

CF 1.f. Student utilizes supervision/consultation effectively, including the need to augment knowledge or to mediate conflict arising from personal values and emotions related to practice and professional contexts.

CF 10(a).b. Student demonstrates the capacity to exercise empathy and use of self in engagement and service delivery.
