# Supplementary material for: International guidelines to inform policy development to address client violence in South Africa: an ATA-document analysis
Source: BMC Health Serv Res. 2022 Aug 12;22:1025. doi: 10.1186/s12913-022-08196-8 (PMC9373364; doi:10.1186/s12913-022-08196-8)
Supplement: Supplementary file 2 — Additional file 2. Data extraction and analysis. [file 12913_2022_8196_MOESM2_ESM.pdf]

DATA EXTRACTION: DOCUMENT REVIEW AND SECONDARY ANALYSIS

ID	Quotation Content	Sub-code	Ref	Theme
31:60	Following each visit, the social workers should report should report back to their supervisor or designated agency representative when the meeting is concluded or as soon as it is safe to do so.	AH	23 - 23	Interaction between the organization and the social worker
31:33	keep emergency contacts on speed dial	AP, DH, TC	17 - 17	Interaction between the organization and the social worker
31:35	agree on and use "code" words or phrases to help social workers convey the nature of threats to their managers or colleagues	AP, DH, TC	17 - 17	Interaction between the organization and the social worker
31:33	keep emergency contacts on speed dial	AP, DH, TC	17 - 17	Interaction between the organization and the social worker
31:35	Agree on and use "code" words or phrases to help social workers convey the nature of threats to their managers or colleagues	AP, DH, TC	17 - 17	Interaction between the organization and the social worker
24:3	Before you leave the office you should fill out a field safety form: • Name and address of clients you are visiting • The specific directions you are taking • Rural setting • Estimated time of arrival and departure • The nature of your visit • Your cell number	PV	13 - 13	Interaction between the organization and the social worker
29:8	it is advisable to know where he or she is going, and to look at a map before driving to unfamiliar areas. In general, remember to be alert, and to lock doors and close windows. The student should tell someone where he or she is going and the expected amount of time she/he will be away from the office. The agency should have your cell phone number or other information on how to contact you in the event of an emergency.	PV	3 - 4	Interaction between the organization and the social worker
30:6	Be sure to inform your supervisor and another colleague of your whereabouts.	PV	2 - 2	Interaction between the organization and the social worker
31:57	Provide addresses of visit and appointment times in the order they are scheduled ■ provide information about the clients being visited ■ indicate the length of each visit (estimated arrival and departure times) ■ provide information about the vehicle they will use (license number, make, model, color) ■ report change of plans to their supervisor or designated agency representative	PV	22 - 22	Interaction between the organization and the social worker
31:58	Example, if a visit or appointment is canceled or delayed) ■ provide information on how to reach them (for example, cell phone)	PV	23 - 23	Interaction between the organization and the social worker
32:2	Social workers should inform supervisors before meeting with clients. It may be necessary to alert security or law enforcement professionals for clients known for their unpredictability and violent history.	PV	3 - 3	Interaction between the organization and the social worker
32:5	Provide clear information to your employer about your whereabouts. Share details about who you'll be visiting, specifying the time and the planned length of your visit.	PV	4 - 4	Interaction between the organization and the social worker
35:1	Notify office of destination with the name, address, phone number, time of visit, and reason for the visit	PV	1 - 1	Interaction between the organization and the social worker

46:46	Workers should also share their schedule with their coworkers or supervisor so others are aware of their whereabouts at all times (Newhill 2012; Newhill & Hagan, 2010; Victor, 2014).	PV	8 - 8	Interaction between the organization and the social worker
46:47	Along with a schedule, workers should provide their supervisor or colleague with the address of where their client visit will take place, the reason for the visit, when they are expected to return, and information regarding the vehicle they will be taking on the visit (Cuadrado & Smith, n.d.; NAIA, 2012; NASW, 2013; Newhill, 2012; NJDCF, 2015; Pope & Hadden, 2011; Syracuse University School of Social Work, 2011).	PV	8 - 8	Interaction between the organization and the social worker
46:48	Workers should also provide their supervisor or colleague with their vehicle information including their license plate number, and make, model, and color of the vehicle they are driving (Cuadrado & Smith, n.d.).	PV	8 - 8	Interaction between the organization and the social worker
46:49	A sign-in/sign-out system may be helpful to track worker client visits and expected departure and arrival times (Pope & Hadden, 2011).<br>Notifying a supervisor or colleague of arrival and departure times helps to ensure someone will check-in and follow-up if a worker does not return when expected (Taylor, 2011).	PV	8 - 8	Interaction between the organization and the social worker
46:50	Any changes in appointment field visits should be reported to the worker's supervisor or agency representative (NASW, 2013).	PV	8 - 8	Interaction between the organization and the social worker
29:7	A student should never see a potentially dangerous client alone without someone else in the agency knowing about the client, the appointment time and the location of the appointment	SS	3 - 3	Interaction between the organization and the social worker
46:9	Once appointments are made, workers should share their schedule with their co-workers and try their best to remain on schedule in order to prevent clients from having to wait (Newhill & Hagan, 2010).	SS	4 - 4	Interaction between the organization and the social worker
32:6	Be prepared with code words or phrases that alert your employer and colleagues to an emergency or a dangerous situation.	SO	4 - 4	Interaction between the organization and the social worker
35:51	Signal a co-worker or supervisor that you need help (try not to let angry client see this, as it may escalate situation)	SO	2 - 2	Interaction between the organization and the social worker
46:134	Any worries or fear regarding transporting a client should be discussed with a supervisor before the trip occurs (Quinn & Mason, n.d.).	TC	15 - 15	Interaction between the organization and the social worker
35:6	Call office before entering home	TS	1 - 1	Interaction between the organization and the social worker
38:7	I was encouraged to sign out the company car rather than using my own, so the agency would know my whereabouts.	TS	1 - 1	Interaction between the organization and the social worker
46:81	Workers are advised to call their office before entering a home or to follow the protocols established by their agency before visiting clients in their home (Syracuse University School of Social Work, 2011).	TS	10 - 10	Interaction between the organization and the social worker
35:68	Do not accept unsolicited offers of assistance	AW	1 - 1	Reflect on your own vulnerabilities
35:41	Avoid giving out personal information, such as address or phone number	AP, DH, TC	2 - 2	Reflect on your own vulnerabilities

35:41	Avoid giving out personal information, such as address or phone number	AP, DH, TC	2 - 2	Reflect on your own vulnerabilities
35:41	Avoid giving out personal information, such as address or phone number	AP, DH, TC	2 - 2	Reflect on your own vulnerabilities
46:101	Workers should avoid giving out personal information such as their home address or phone number to their clients (Syracuse University School of Social Work, 2011).	DH	12 - 12	Reflect on your own vulnerabilities
3:3	Wear comfortable shoes and clothes that allow you to move easily.	PV	1 - 1	Reflect on your own vulnerabilities
16:9	Make first appointments for early in day. Day-light hours are more safe until you know the area and the client	PV	11 - 11	Reflect on your own vulnerabilities
16:13	Dress appropriately-no jewelry.	PV	12 - 12	Reflect on your own vulnerabilities
24:1	Should you wear jewelry?<br>• Should you wear high heels?<br>• Should you carry a purse?	PV	10 - 10	Reflect on your own vulnerabilities
29:5	And what to wear.	PV	3 - 3	Reflect on your own vulnerabilities
29:9	It is helpful to dress in comfortable clothes that are loose fitting, and to wear sturdy, flat walking shoes.	PV	4 - 4	Reflect on your own vulnerabilities
30:15	Always wear comfortable clothes and shoes while working in the field since you will be regularly walking, standing and climbing stairs.	PV	3 - 3	Reflect on your own vulnerabilities
30:16	If you are highly allergic to certain domestic animals then you should take that into account before conducting home visits. Many clients live with cats, dogs and other pets.	PV	3 - 3	Reflect on your own vulnerabilities
31:41	Does the client have a communicable disease?<br>■ Assessment of worker vulnerability ◎ Working alone ◎ Visible physical conditions that may increase vulnerability (pregnancy, disabilities, use of cane or walking aid)	PV	19 - 19	Reflect on your own vulnerabilities
31:42	Lack of experience ◎ Appearing timid, vulnerable, lost, or confused ◎ Lax attitude and/or overconfidence ◎ Worker bias or stereotyping that causes over- or underreaction to safety threats ◎ Attire (wearing jewelry and other valuables, high-heeled shoes, and so forth) that adds to vulnerability ◎ Accessories (political buttons, religious jewelry) that may trigger reactions ◎ Appearance (for example, tattoos, body piercings) that cannot be covered and that might attract/increase attention ◎ Lack of a safety plan	PV	20 - 20	Reflect on your own vulnerabilities
32:22	Wearing proper attire facilitates ease of movement, and minimal jewelry makes social workers less of a potential target.	PV	6 - 6	Reflect on your own vulnerabilities
32:24	Social workers should call ahead of an appointment to check if a family has pets.	PV	7 - 7	Reflect on your own vulnerabilities
35:8	Wear clothes and shoes that provide freedom of movement	PV	1 - 1	Reflect on your own vulnerabilities
38:5	I was taught to “fit in” with my surroundings in the way I dressed.	PV	1 - 1	Reflect on your own vulnerabilities
38:11	Another tip was to avoid wearing a lanyard keychain around my neck, as someone could easily grab it and cut off my breathing.	PV	1 - 1	Reflect on your own vulnerabilities

46:60	Before departing on a field visit, workers should ensure they are wearing appropriate clothes and shoes that allow for prolonged walking, standing, or climbing stairs and ample movement (Nelson, n.d.; Syracuse University School of Social Work, 2011; Taylor, 2011; Victor, 2014).	PV	9 - 9	Reflect on your own vulnerabilities
46:61	Workers should not wear accessories that can be easily pulled including a tie, necklace, or earrings and should pull back long hair (Nelson, n.d.; Taylor, 2011).	PV	9 - 9	Reflect on your own vulnerabilities
46:62	Additionally, workers should not wear expensive clothing items including watches, jewelry, or purses (NAIA, 2012; Quinn & Mason, n.d.).	PV	9 - 9	Reflect on your own vulnerabilities
35:53	Avoid giving personal information during interview	PC	2 - 2	Reflect on your own vulnerabilities
46:78	Workers should refrain from providing those that are unfamiliar with their name, street address, and information regarding where they work. If they encounter anyone as they walk to their client's home, workers should keep normal, confident eye contact (Taylor, 2011).	TS	10 - 10	Reflect on your own vulnerabilities
31:10	regularly gather and disseminate information about all safety risks including assaults, threats, and abuse and develop strategies for managing them via case consultations, training and education, and policy development.	DM, PP, ST	13 - 13	Continious training on social work safety
31:14	orientation and training needs of staff for risk reduction and safety promotion	DM, ST	14 - 14	Continious training on social work safety
31:14	orientation and training needs of staff for risk reduction and safety promotion	DM, ST	14 - 14	Continious training on social work safety
46:133	a sign-in/sign-out system, a buddy system, and education and training sessions for staff.	DH, PV, ST	14 - 14	Continious training on social work safety
46:163	Agencies should produce and manage an organizational culture that promotes safety and security for their staff (NASW, 2013).<br>o To ensure an organizational climate of safety in every office, management should conduct regular safety discussions during both staff trainings and meetings (Newhill & Hagan, 2010).<br>o Management should notify all employees of its commitment to promote the safety of all staff members both verbally and in writing (NASW, 2013).	PP, ST	20 - 20	Continious training on social work safety
26:1	This bill provides states with critical resources designed to alleviate workplace violence threats by allowing grant money to be used to purchase<br>safety equipment, make facility improvements, facilitate safety training programs, provide support services for social workers who have been victims of violence, or track incident data to mitigate future offenses against social workers, among other important uses.	PI, SI, SO	1 - 2	Continious training on social work safety
31:6	provides ongoing proactive risk assessment that identifies line staff at risk for violence, precarious settings and working conditions, as well as orientation and in-service training on practices that can reduce or minimize or eliminate factors associated with elevated risk.	SC, ST	12 - 12	Continious training on social work safety
1:5	safety courses,	ST	2 - 2	Continious training on social work safety
1:8	adequate training or hazard pay	ST	2 - 2	Continious training on social work safety

1:9	seek out that training – which may be in a workshop or through the literature.	ST	4 - 4	Continious training on social work safety
3:9	Attend training on conflict resolution, personal safety, teamwork and stress reduction.	ST	1 - 1	Continious training on social work safety
25:2	provide safety training to staff	ST	1 - 1	Continious training on social work safety
26:2	adequate funds to properly train and protect public workers are not prioritized. The Social Worker Safety Act aims to provide states with these much-needed resources.	ST	3 - 3	Continious training on social work safety
26:7	and annual training.	ST	7 - 7	Continious training on social work safety
29:14	Students should be alerted to the existence of biohazards. They should receive training and information about how to protect themselves from infectious diseases.	ST	4 - 4	Continious training on social work safety
31:45	Social workers should be well trained in the use of their agency's risk assessment instrument and supervised to ensure consistency in practice.	ST	20 - 20	Continious training on social work safety
31:74	Safety training can include skill building in risk assessment, risk management, risk reduction, a SafetyStandards_NASWCulturalStandards2003.Q4.11 3/14/13 10:44 AM Page 22 previously constructed Safety Plan of Action that includes exit strategies, verbal de-escalation techniques, effective strategies for clinical interventions with violent or potentially violent clients, and nonviolent self-defense and the impact of secondary trauma	ST	24 - 25	Continious training on social work safety
32:12	Personal safety training for social workers can help social workers prevent and address the challenges.	ST	4 - 5	Continious training on social work safety
32:13	education programs can help social workers manage stress more effectively using these coping skills.	ST	5 - 5	Continious training on social work safety
32:18	workplace safety training	ST	5 - 5	Continious training on social work safety
32:19	Personal safety training helps social workers understand and prepare for dangerous scenarios.	ST	5 - 5	Continious training on social work safety
32:20	Training programs can cover communication-related concepts, such as speaking calmly with a clear and direct approach.	ST	5 - 5	Continious training on social work safety
32:21	Risk assessment is another critical part of safety training. For example, situational awareness can include assessing a client's history, including with previous social workers. Other areas covered in training may consist of engaging in crisis communication, identifying rage and triggers, and treating violent clients.	ST	5 - 5	Continious training on social work safety
33:3	mplement safety training programs	ST	1 - 1	Continious training on social work safety
41:17	Support structures for social workers can also be seen as competency built through ongoing vocational training and introductions.	ST	9 - 9	Continious training on social work safety
