# Supplementary material for: International guidelines to inform policy development to address client violence in South Africa: an ATA-document analysis
Source: BMC Health Serv Res. 2022 Aug 12;22:1025. doi: 10.1186/s12913-022-08196-8 (PMC9373364; doi:10.1186/s12913-022-08196-8)
Supplement: Supplementary file 3 — Additional file 3. Finelized codebook. [file 12913_2022_8196_MOESM3_ESM.pdf]

CODEBOOK: ATA DOCUMENT ANALYSIS

PHASE 1 CODING (The initial step in data classification is to get a comprehensive picture of the various data types)				
	Code label	Definition	Example(s)	Exclusions
1	**Prevention and Management of Client Violence**	Client violence is clinically managed to prevent or lessen the perceived level of threat. This intervention is frequently on an administrative or organizational level.	"...establish safety teams or safety committees to ensure adherence to policy and procedure." "...create a mechanism for reporting and collecting data on an ongoing basis on incidents of assaults, threats, and abuse that can be analysed to inform the agency about the incidence and prevalence of violence to guide the development of safety protocols and allocation of resources."	Physical measures that are built into structures are not included.
2	**Office Safety**	The process of ensuring that employees and visitors are safe while at work.	"Internal alert systems that can be activated from panic buttons in offices". "Assess possible weapons (books, scissors, clip board, keys, pen, umbrella) you may have at your disposal, or that could be used against you."	Anything that occurs in the field or that can be classified as administrative in nature.
3	**Home Visits**	Keeping yourself safe while visiting a client's residence. Everything from the time the social worker leaves the office until they safely return is covered.	Take a “buddy” or law enforcement with you on potentially dangerous home visits. "Always be vigilant and assess the surroundings—both inside and outside a client’s home." "As you enter the home, notice the layout, exits and phones. Position yourself for an easy exit should you need to leave quickly."	Traveling with or transporting clients is not permitted.
4	**Transporting of Clients**	While transporting a client between two points, safety risks must be considered. The conditions of the vehicle is also important when considering these risks.	"If client is agitated or aggressive- do not transport- use police or ambulance". "Make certain your car has gas, water, a spare tire with jack, a working horn, spare change, a flashlight, jumper cables and a first aid kit."	This category excludes any elements connected to home visits, office visits, or administrative protocols.

5	**Post-Incident Protocols**	Anything that happens following a client violence incident is included in post-incident protocols, which includes, but is not limited to, investigations and support.	"When investigating an incident, police reports should be obtained and assessed to determine injuries or harm to certain departments or units; employee work spaces, job positions, employee tasks, or times of day". "Providing financial compensation for damage to property". "Offering voluntary referrals to Employee Assistance program services"	This category does not include administrative processes.
**PHASE 2 CODING (Detailed categorisation of codes)**				
	**Code label**	**Definition**	**Example(s)**	**Exclusions / Comments**
**CODE 1: Prevention and Management of Client Violence**				
1.1	**Data management**	Collecting, storing, and using current and historical data about client violence in a secure, efficient, and cost-effective manner.	"Create a mechanism for reporting and collecting data on an ongoing basis on incidents of assaults, threats, and abuse that can be analysed to inform the agency about the incidence and prevalence of violence to guide the development of safety protocols and allocation of resources."	N/A
1.2	**Policies**	An organization's course of action or philosophy of action for dealing with client violence. This is primarily preventative or administrative in nature, and it includes suggestions for what should be included in the policy.	Safety Policies that provide an oral and written commitment by agency leadership to promote the safety of all staff, including support, paraprofessional, and professional staff , govern the management of dangerous (or hostile and violent) behaviour in the workplace (including clients, co-workers, and supervisors)	Physical measures that are built into structures are not included.
1.3	**Reporting**	Reporting is the act of disclosing details about a client violence occurrence. The motivation or practices supplied by organizations to improve this process fit into this category.	"Set guidelines for reporting incidents of workplace violence available for all employees"	N/A

1.4	**Safety Committees**	Literature highlighting the necessity, role, or need for a safety committee in a social work organization to address client violence.	"A Safety Committee that oversees the conscientious adoption, use, and ongoing review of the agency's policies that underscore the commitment to safety for staff, clients, administration, and governing boards or ensures that safety protocols that are instituted, updated, and practiced regularly.	The word "committee" may also be substituted with the word "team".
1.5	**Safety Training**	Literature that emphasizes the necessity of social worker training and specifies the types of training that should be considered.	Safety training can include skill building in risk assessment, risk management, risk reduction, a previously constructed Safety Plan of Action that includes exit strategies, verbal de-escalation techniques, effective strategies for clinical interventions with violent or potentially violent clients, and nonviolent self-defence and the impact of secondary trauma.	All information on defusing techniques are included in this section.
	**Code label**	**Definition**	**Example(s)**	**Exclusions / Comments**
**CODE 2: OFFICE SAFETY**				
2.1	**Arriving at work**	This category includes processes for ensuring safety from the moment the social worker enters the employment building's premises.	"Upon arriving to work, it is suggested for workers to remain in their vehicle and conduct a visual scan of the parking lot for unfamiliar or sceptical vehicles"	This section does not include physical measures within the office.
2.2	**Preparation for clients**	When preparing for client meetings, the social worker should have procedures in place to ensure safety.	"Comprehensive risk assessments of both clients and work settings"	This section only encompasses what the social worker does before meeting with the client.
2.3	**Creating safe interview settings**	Several procedures and approaches can be used to ensure client safety during office interviews.	"Leave door open during session, if there are others around who could come to your aid. "Restricted access to objects that may be used as weapons." " Avoid seeing clients alone after hours."	This section is related to strategies in office interviews.

2.4	**Security in the office**	This category includes physical methods that can help to improve overall office safety.	"Use of safety technology (e.g. mobile panic buttons, security cameras). "Secure entry and access (for example, monitored, restricted access security guard, metal detector screening, bulletproof glass)". "Well-lit hallways that lead to employees' workspaces."	Only physical measures included.
	**Code label**	**Definition**	**Example(s)**	**Exclusions / Comments**
		**CODE 3: HOME VISITS**		
3.1	**Planning a visit**	The tasks conducted by the social worker prior to visiting the client's home are referred to as "planning a home visit." Exploring environmental elements, the client's living space, the client's condition, the state of emergency equipment, and recognizing aspects that may contribute to the worker's own vulnerability are just a few examples.	"Dress appropriately-no jewellery. "If there are any safety concerns with the neighbourhood plan early a.m. appointments". "If you are highly allergic to certain domestic animals then you should take that into account before conducting home visits". "Get to know the neighbourhood of the home you plan to visit. Before arriving at your appointment, use Google Maps to familiarize yourself with the area, including locating the nearest police station".	Exclude measures that is taken when travelling to the site, during or after the home visits.
3.2	**Travelling to site**	This section outlines all of the precautions that the social worker should take while travelling to the client's home.	Travel with a cell phone. Keep it on and programmed to call 911 for help in any emergency or threatening situation. "Park in well-lit, visible area and lock the car doors (do not park in the driveway or directly in front of the house)".	N/A
3.3	**During a Home visit**	This section discusses the safety considerations that social workers should keep in mind from the time they enter a client's home until they leave.	"If person or persons you made visit with are not available, LEAVE. "Control where on the home the meeting takes place".	N/A

3.4	**After a Home visit**	Safety precautions once the social worker leaves the client's house and returns to the office.	Following each visit, the social workers should report should report back to their supervisor or designated agency representative when the meeting is concluded or as soon as it is safe to do so. "If you are being followed, do not drive home; drive to nearest police or fire station and honk your horn or drive to open gas station or business where you can safely call the police".	N/A
	**Code label**	**Definition**	**Example(s)**	**Exclusions / Comments**
			**CODE 4: TRANSPORTING OF CLIENTS**	
4.1	**Assessment at pickup**	Consider these factors when a social worker picks up a client for transportation. There are also some safety advice included to ensure that the transportation process is as safe	"Continually remain aware of mental status of client". "The client's level of agitation (if any), use of intoxicants, and the meaning of the appointment to the client" . "The possibility that the client has a weapon".	Aspects of the vehicle's condition, as well as conditions that are directly related to children, are not included.
4.2	**Transporting children**	Requirements of general safety when transporting clients, with a special focus on conditions relating to children.	"Engage the child safety locks in the vehicle. "Seating behind passenger seat."	Conditions of the vehicle are excluded.
4.3	**Vehicle condition**	The general safety conditions of the transportation vehicle are specified.	"Make certain your car has gas, water, a spare tire with jack, a working horn, spare change, a flashlight, jumper cables and a first aid kit."	N/A
	**Code label**	**Definition**	**Example(s)**	**Exclusions / Comments**
			**CODE 5: POST-INCIDENT PROTOCOLS**	
5.1	**Post-Incident Protocols**	Anything that happens following a client violence incident is included in post-incident protocols, which includes, but is not limited to, investigations and support.	"When investigating an incident, police reports should be obtained and assessed to determine injuries or harm to certain departments or units; employee work spaces, job positions, employee tasks, or times of day". "Providing financial compensation for damage to property". "Offering voluntary referrals to Employee Assistance program services"	This category does not include administrative processes.
