# Supplementary material for: International guidelines to inform policy development to address client violence in South Africa: an ATA-document analysis
Source: BMC Health Serv Res. 2022 Aug 12;22:1025. doi: 10.1186/s12913-022-08196-8 (PMC9373364; doi:10.1186/s12913-022-08196-8)
Supplement: Supplementary file 4 — Additional file 4. List of included literature. [file 12913_2022_8196_MOESM4_ESM.pdf]

ANNEXURE B – INCLUDED DOCUMENTS

Cuadrado, O. & Smith, H. (n.d). Worker safety. Retrieved from https://cdn.ymaws.com/www.naswma.org/resource/resmgr/imported/Safety_CT_DSS_WorkerSafetyTrainingPowerPoint-5.pdf

Hardy, V. (2016). *Safety Awareness for Social Workers: Tips Learned Along the Way*. 18–20.

Ne, F. S. (2020). *Protecting Social Workers and Health Professionals from Workplace Violence Act of 2019 (S . 2880 / H . R . 5138)*. (January), 20002.

Newhill, C. (n.d). Client violence and Social Work Safety: Lunchtime Webinar Series. Retrieved from https://cdn.ymaws.com/www.naswoh.org/resource/resmgr/imported/Client%20Violence%20and%20Social%20Worker%20Safety.pdf

Nordesjö, K. (2020). Instrumental, Interpersonal or Holistic: Social Work Managers' Conceptions of Safety in the Psychosocial Work Environment. *Journal of Social Service Research*, *46*(6), 789–800. https://doi.org/10.1080/01488376.2019.1658690

Ohio University. (2021). *Social Worker Safety: Tips and Training for Social Workers*. Retrieved from https://onlinemasters.ohio.edu/blog/social-workers-safety/

Ork, C., & Ide, P. G. (2021). *Casework Practice Guide.* Retrieved from https://www.msf.gov.sg/ODGSW/documents/Casework%20Practice%20Guide%20-%20Final.pdf

Pascale, V. (2014). Safety Tips for Home Visits From a Veteran NYC Social Worker. Retrieved June 17, 2021, from NASW New York City Chapter website: https://www.naswnyc.org/page/489

Personal Safety Tip Sheet for Human Service Workers. (2016). 2016.

Quinn, P., & Ph, D. (n.d.). *Personal Safety in Clinical Practice We must remain continually aware of safety issues in our daily clinical interactions*.

Simmons School of Social Work Field Education Department. (2021). *Safety Policy and Procedures*. Retrieved from https://internal.simmons.edu/students/academics/ssw/msw-students/field-education/safety-policy-and-procedures

Stowe, S. (2015). Literature review: Worker Safety. *Southern Area Consortiumof Human Services 19* (November), 33–37.

Syracuse University School of Social Work. (2011). Social worker safety tips. Retrieved from http://falk.syr.edu/socialWork/documents/2011/BSW/Safety Tips 2011.pdf.

The National Association of Social Workers (NASW). (2013). *Guidelines for Social Work Safety in the Workplace.* Retrieved from https://www.socialworkers.org/LinkClick.aspx?fileticket=6OEdoMjcNC0%3D&portalid=0

The National Association of Social Workers (NASW). (2019a). *Lawmakers introduce bipartisan bill to improve social worker safety*. Retrieved from https://www.socialworkers.org/News/News-Releases/ID/1999/Lawmakers-introduce-bipartisan-bill-to-improve-social-worker-safety

The National Association of Social Workers (NASW). (2019b). *NASW CEO Angelo McClain urges Congress pass "Workplace Violence Prevention for Health Care and Social Service Workers Act."* Retrieved from https://www.socialworkers.org/News/News-Releases/ArticleType/ArticleView/ArticleID/1949

To, S., Career, A. G., & Social, I. N. (2012). *Leadership adders : organizing for office safety Communicate Concerns Ahead*. (April)
