# Supplementary material for: International guidelines to inform policy development to address client violence in South Africa: an ATA-document analysis
Source: BMC Health Serv Res. 2022 Aug 12;22:1025. doi: 10.1186/s12913-022-08196-8 (PMC9373364; doi:10.1186/s12913-022-08196-8)
Supplement: Supplementary file 5 — Additional file 5. Research plan and ATA process followed 1. [file 12913_2022_8196_MOESM5_ESM.pdf]

DOCUMENT ANALYSIS (PHASE 3)
ANALYSIS PLAN

Applied Thematic Analysis of various qualitative and quantitative documents and secondary resources is intended for a single study.

Persons involved in the analysis

Researcher

Research supervisor

Co-coder

Research question informing analysis:

What international policies, frameworks, protocol and guidelines can inform the development of a policy framework to enhance the protection of South African social workers against client violence?

Data to be analysed:

- International policies, frameworks, protocol, guidelines and legislation as provided by the NASW;
- International policies, frameworks, protocol, guidelines and legislation identified by the NWU's academic search database when using predetermined search protocol: *"social work*" AND "client violence*" OR "client aggression*" OR "service user violence*" AND "policy*" OR "framework*" OR "protocol*" OR "guideline*" OR "legislation*";*

Primary analytic purpose

The Applied Thematic Analysis will be inductive, exploratory as well as explanatory in nature.

Proposed Applied Thematic Analysis Process to be followed:

- Planning and Preparation;
- Data Gathering;
- First-level analysis;
    - Reading and preliminary interpretation of the contents of the entire dataset;
    - Start with Key-word-in-context (KWIC) search as locus for concepts in the text;
    - Start developing codebook by incorporating elements of text directly into initial code definitions to illuminate the emerging themes;
    - Modify the codebook as new information and insight is gained;
    - Flag poor data with a generic code "Unclear_Data".
- Second-level analysis;
    - Multiple iteration of the coding development, coding and checking;
    - Intercoder agreement checks and adaption of codebook;
    - Refine codes and thematic analyses;
- Third-level analysis;
    - Applying counting rules, counting the occurrences of codes applied to the data sets and interpreting the results, thus quantifying qualitative data by describing code frequencies and thematic content.

Plan to enhance validity and reliability

- **Using multiple methods and data sources**: collecting data from a variety of sources provides the opportunity to compare findings in analysis for convergence or divergence;
- **Develop and use a precise codebook:** This will ensure better intercoder reliability and facilitate data comparison if using the same codes in a different study;
- **Use multi-coders and intercoder agreement checks:** Use of multiple coders facilitates coding reliability by providing checks on individual biases and variance in interpretation of code definitions. The iterative revision of the codebook as a result of the coding checks improves the precision of the codebook;
- **Creating an audit trail**: Documentation of analysis steps and codebook revisions makes the analysis process more transparent for other researchers to review and facilitates the ability to accurately replicate procedures if desired;
- **Triangulate findings:** Convergent data from different sources validate findings.

ACTUAL ATA - PROCESS FOLLOWED

1. Planning and preparation;

2. Data gathering:
   - 2.1. Identify documents as proposed by NASW, as well as documents found by using the proposed search criteria;
   - 2.2. Upload all documents to ATLAS T.I data-management program;
   - 2.3. Delete documents that are older than 10 years and therefore no longer relevant;
   - 2.4. Finalize documents that will be included in this study – list available as **Annexure B.**

3. Reading and preliminary interpretation of the contents of the entire dataset.

4. Start with Key-word-in-context (KWIC) search as locus for concepts in the text.

5. Commence PHASE 1 coding:
   - 5.1. Start developing codebook by incorporating elements of text directly into initial code definitions;
   - 5.2. Assigning basic codes to data: **Prevention and Management of Client Violence**, **Office Safety**, **Home Visits**, **Transporting of Clients** and **Post-Incident Protocols**;
   - 5.3. Modify the codebook as new information and insight is gained.

6. Commence PHASE 2 coding:
   - 6.1. Explore the existing codes and relevant data by grouping relevant aspects;
   - 6.2. Modify the codebook as new information and insight is gained;
   - 6.3. Extent basic codes to include:
     - **Management of Client Violence*:*** Data management, Policies, Reporting, Safety Committees, Training
     - **Office Safety:** Arriving at work, Safe interview settings, Security in the office, Prep for clients, Defusing techniques
     - **Home Visits:** Planning a visit, Travelling to site, During a home visit, After a home visit
     - **Transporting of Clients:** Assessment at pickup, Transporting children, Vehicle condition
     - **Post-Incident Protocols**

7. Modify and develop the codebook in accordance

8. Consider intercoder agreement – Atlas.Ti's function will be utilized for this purpose.

9. Finalize documents and refer to co-coder.
