# Supplementary material for: International guidelines to inform policy development to address client violence in South Africa: an ATA-document analysis
Source: BMC Health Serv Res. 2022 Aug 12;22:1025. doi: 10.1186/s12913-022-08196-8 (PMC9373364; doi:10.1186/s12913-022-08196-8)
Supplement: Supplementary file 6 — Additional file 6. Research plan and ATA process followed 2. [file 12913_2022_8196_MOESM6_ESM.pdf]

**CO-CODER PROCESS**

**Process description**

1. Planning and preparation:

- Familiarisation with background, research question, research aim etc.,
- Familiarisation with methodology

2. First level analysis – applying pre-identified codes to the literature – 17 articles, guidelines, protocols in total

I first went through all the literature and assigned the 5 identified codes

1. Prevention and Management of Client Violence
2. Office Safety
3. Home Visits
4. Transporting of Clients
5. Post-Incident Protocols

During this step I also made notes about possible sub-themes that I saw emerging and highlighted phrases that could serve as definitions.

3. Second level analysis – refining codes and making suggestions for codes that don't appear in the codebook that was shared. At first glance, the sections in the literature seemed like they could be categorised into a broader code (1, 2, 3, 4, & 5) but on the second level analysis and having more specific codes, these changed to different categories.

4. Finally, I modified the codebook as needed: definitions, examples and sub-themes. See my comments / suggestions / changes in orange.
